# Comparative Reduction of Pharmaceutical Hazards in Wastewater among Pilot-Scale Quaternary Treatment Technologies across Seasons

**DOI:** 10.1021/acsenvironau.6c00068

**Published:** 2026-08-01

**Authors:** Michal Bittner, Lucie Báborská, Kateřina Bočková, Anna Ireinová, Marek Holba, Jaroslav Lev, Daniel Polášek, Lucie Bláhová, Bryan W. Brooks, Luděk Bláha

**Affiliations:** † 37748 Masaryk University, Faculty of Science, RECETOX, Kamenice 5 625 00, Brno, Czechia; ‡ ASIO TECH, spol. s r.o., Kšírova 552/45 619 00, Brno, Czechia; § AQUA PROCON, s.r.o., Palackého tř.12 612 00, Brno, Czechia; ∥ Department of Environmental Science, Center for Reservoir and Aquatic Systems Research, 14643 Baylor University, Waco, Texas 76798, United States

**Keywords:** wastewater, quaternary treatment, pilot-scale, pharmaceuticals, recycling, novel entities

## Abstract

For wastewater to provide freshwater resources, reclaimed water should not contain pharmaceutical active compounds (PhACs) or other contaminants that pose significant ecological or health risks. This consideration addresses a recent European Union (EU) wastewater regulation mandating an 80% removal efficiency (RE) for indicator PhACs. We investigated REs of six different quaternary treatment technologies (QTTs) over a year-long study to identify seasonal variability and potential environmental risks of PhACs in recycled water. Wastewater from both a WWTP effluent and subsequent QTT effluents was sampled approximately every two to six weeks and processed by solid-phase extraction, and 12 indicator PhACs were analyzed by LC–MS/MS. Though no RE was observed for ultrafiltration (UF) and limited RE for granular activated carbon (GAC) and GAC + UV, advanced treatment with ozonation (UF + O_3_) and reverse osmosis (UF + GAC + UV + RO) achieved over 99% RE for most PhACs during both cold and warm seasons. Probabilistic environmental hazard assessments demonstrated that even after QTT, some PhACs, particularly diclofenac, remained at concentrations that exceeded predicted no-effect concentrations, indicating the need for continued improvement in wastewater treatment and water reclamation strategies.

## Graphical abstract

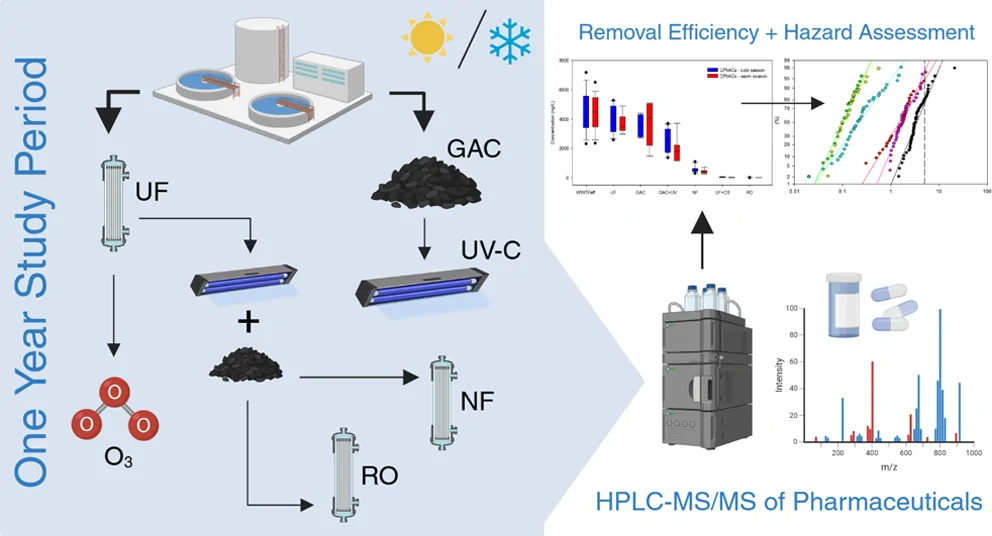

## 1 Introduction

Climate change, urbanization, and water pollution have significantly affected water supplies worldwide. According to the United Nations, water scarcity is one of the most urgent challenges billions of people have been facing. To address this challenge, a comprehensive approach including circular economy principles, advanced wastewater treatment, and reuse strategies is necessary.^1^ Water reuse has been supported at the EU level by the recent Regulation 2020/741 “on minimum requirements for water reuse” for agricultural and environmental applications.^2^ This reflects the reality in many European countries, particularly in Mediterranean regions that suffer most from water scarcity and frequent droughts. Conversely, waste reuse strategies in Central and Eastern European countries, which have not yet experienced severe water stress, are less developed, especially at the legislative level.^3^ Another EU-wide milestone in regulating wastewater quality regarding contaminants of emerging concern is the recent Revision of the EU Urban Wastewater Treatment Directive – UWWTD.^4^ According to the UWWTD, urban wastewater must undergo secondary treatment in all communities with >1000 population equivalent (PE) by 2035; tertiary treatment (i.e., removal of N and P) must occur in all wastewater treatment plants (WWTPs) serving >150,000 PE by 2039 and >10,000 PE by 2045. Further, quaternary treatment removing a broad spectrum of micropollutants, an important and diverse group of novel entities, will be mandatory for all WWTPs servicing >150,000 PE by 2045.^4^ The UWWTD, among others, requires quaternary treatment of WWTP discharges, which includes at least 80% removal of selected pharmaceutical active compounds (PhACs) in relation to influent sewage loading.

This new directive reflects the reality of the increasing load of PhACs into the environment, where a requirement of at least 80% PhACs removal efficiency should decrease this environmental risk. To identify whether PhACs in reclaimed wastewater potentially present adverse effects in aquatic ecosystems, aquatic predicted no-effect concentration (PNEC) values with appropriate assessment factors for ecotoxicity data^5^ and development of antibiotic resistance^6^ can be used during the performance of probabilistic environmental hazard assessments (PEHA) of PhACs.^7–11^ PEHAs can be particularly useful to examine wastewater treatment efficiency.^12,13^

Here, we considered common PhACs, including four of the 12 PhACs listed in the UWWTD (carbamazepine, diclofenac, hydrochlorothiazide, clarithromycin). Carbamazepine, diclofenac, and hydrochlorothiazide were included in a previous study of PhAC REs across ten conventional WWTPs, with mean REs of 27.5%.^14^ Beyond traditional secondary WWTP treatment, additional quaternary treatment technologies (QTTs) will be needed to reach the goal of a PhAC RE of 80% during the WWTP process. According to recent data, QTTs employing ozonation and activated carbon treatment (both well-established for the removal of PhACs) are currently implemented in 11 EU member states, though the majority are located in Germany and Italy. However, only a fraction of these installations are operational in WWTPs with a capacity of >150 000 PE.^15^

In the current study, we performed a unique pilot-scale study over one year to examine REs of QTT connected to a conventional WWTP with secondary treatment. REs were evaluated by measuring 12 PhACs in the WWTP effluent (WWTPeff.), and corresponding samples were treated with six different QTTs. Sampling took place from March 2021 to March 2022 to cover both the warm and cold seasons of the year. We employed traditional grab sampling with subsequent solid-phase extraction (SPE) and LC–MS determination of PhACs, which were selected concerning their significance in the European freshwater and legislation (Table 1). We then performed PEHAs to determine whether differential hazard reductions for PhACs were achieved across the six different QTTs.

**Table 1:** List and Properties of Pharmaceuticals Analyzed in Both WWTP Effluent and Quaternary Treated Water Samples^a^

acronym	compound	drug class	chemical class	Log K_OW_^a^	solubility^a^ (mg/L)	charge^b^	significance	LOQ (ng/L)	PNEC^c^ (ng/L)	PNEC-AR^d^ (ng/L)
ACET	acetaminophen	analgesics	aminophenols	0.46	14,000	0	1	1	500	
ATEN	atenolol	b-blockers	ethanolamines	0.16	13,300	+1	1	1	100,000	
CARB	carbamazepine	anticonvulsants	tricyclic antidepres	2.45	152	0	1, 2, 3	1	10	
CLAR	clarithromycin	antibiotics	macrolides	3.16	0.33	+1	1, 2, 3, 4	1	20	250
DICL	diclofenac	NSAID	phenylacetic acids	4.51	2.37	–1	1, 2, 3, 4	1	1	
ERYT	erythromycin	antibiotics	macrolides	3.06	2000	+1	2, 4	1	200	1000
HYDR	hydrochlorothiazide	diuretics	benzothiadiazines	–0.07	722	0	1, 3	1	56,200	
IBUP	ibuprofen	NSAID	propionic acids	3.97	21	–1	1, 2, 4	5	10	
KETO	ketoprofen	NSAID	propionic acids	3.12	51	–1	1	1	1240	
NAPR	naproxen	NSAID	propionic acids	3.18	15.9	–1	1	1	36,700	
SULF	sulfamethoxazole	antibiotics	sulfonamides	0.89	610	–1	1, 4	1	2400	16,000
TRIM	trimethoprim	antibiotics	trimethoxybenzyl-pyrimidines	0.91	400	+1	1, 4	1	15,700	500

a NSAID—non-steroidal anti-inflammatory drug, LOQ —limit of quantitation, PNEC—predicted no-effect concentration, and PNEC-AR—predicted no-effect concentration for antibiotic resistance. Significance explanation: (1) PhACs from the “List of substances to reduce pharmaceutical pollution of watercourses in the Czech Republic”;^17^ PhACs prioritized in the European Demonstration Program based on NORMAN risk score;^18^ (3) PhACs from the UWWTD;^4^ (4) PhACs from the Watch Lists of the EU Water Framework Directive;^19–21^ a—Log K_OW_ and solubility in water at 25 °C from PubChem (https://pubchem.ncbi.nlm.nih.gov/), b—charge at pH 7 from Drugbank (https://go.drugbank.com/drugs/), c—PNEC from Zhou et al. (2019),^5^ and d—PNEC-AR from Bengtsson–Palme & Larsson (2016).^6^

## 2 Methods

### 2.1 Literature Review

We conducted a literature review of the efficiency of pilot-scale QTTs in Scopus using the keywords wastewater, treatment, pharmaceuticals, pilot, and scale. We found 359 documents of which 19 were relevant to the review; these are summarized in Tables 2 and S13.

**Table 2:** Summary of the Effectiveness of Removal of Pharmaceuticals (PhACs) by Quaternary Treatment Technologies (QTTs) in both Cold and Warm Seasons of the Year, and Corresponding Data from the Literature (liter—the Third Column for Each QTT; Literature Is Listed in Table S13)­^a^

compound	UF			GAC			GAC+UV			UF+GAC+UV+NF			UF+O3			UF+GAC+UV+RO		
	cold	warm	liter	cold	warm	liter	cold	warm	liter	cold	warm	liter	cold	warm	liter	cold	warm	liter
ACET	-	-	20–50	-	-	ND	–50.0	–29.5	ND	61.6	-	>80	92.2	92.2	>80	92.9	78.3	>80
ATEN	-	-	<20	56.0	16.9	>80	45.5	53.5	ND	96.1	96.2	50–80	99.5	99.4	>80	99.4	99.2	>80
CARB	-	7.0	<20	36.7	9.2	50–80	29.7	31.6	>80	83.3	83.4	>80	99.9	99.9	>80	99.9	99.9	>80
CLAR	-	-	ND	30.7	-	>80	11.2	8.9	>80	99.1	99.5	>80	99.6	99.2	>80	99.6	99.8	>80
DICL	-	-	20–50	-	-	>80	54.3	63.7	>80	97.1	97.0	>80	99.7	99.9	>80	99.8	99.9	>80
ERYT	-	-	ND	31.1	-	>80	9.8	5.4	ND	98.0	99.4	>80	99.1	98.6	>80	99.6	99.5	>80
HYDR	-	-	<20	78.7	24.1	>80	58.4	48.2	ND	61.8	66.2	ND	99.9	99.9	>80	99.8	99.7	>80
IBUP	-	-	<20	-	-	>80	67.8	N/A	ND	91.2	N/A	>80	81.4	81.6	50–80	93.8	N/A	>80
KETO	-	-	20–50	N/A	-	ND	95.7	52.5	ND	98.6	96.2	ND	91.6	94.5	50–80	98.4	98.2	>80
NAPR	-	-	<20	56.1	17.0	ND	32.1	43.3	ND	91.1	92.5	>80	99.5	99.8	>80	99.7	99.7	>80
SULF	-	-	20–50	-	-	>80	29.8	17.6	>80	82.9	78.9	>80	99.6	99.6	>80	99.9	99.9	>80
TRIM	-	-	20–50	65.6	13.8	>80	46.3	42.5	>80	93.6	93.7	>80	99.6	98.6	>80	99.6	98.6	>80
ΣPhACs	-	-	ND	36.4	19.5	-	41.7	47.3	-	86.6	87.6	-	98.9	99.6	-	99.7	99.7	-

a Values show a median percentage decrease in individual PhAC (and ΣPhACs) concentrations compared to the concentrations of PhACs (and ΣPhACs) in corresponding WWTPeff. samples (paired comparison). The table depicts only statistically significant REs (statistically non-significant are marked with -). ND—not detected, N/A—insufficient data for statistical analysis (less than four samples). The acronyms for pharmaceuticals are defined in Table 1.

### 2.2 Wastewater for Quaternary Treatment

Pilot technologies for quaternary wastewater treatment were installed at the WWTP Brno-Modřice (Czechia). We thus examined QTTs using secondary-treated effluent from this WWTP, which is commonly discharged into the Svratka River in accordance with EU and Czech legislation. The WWTP is a mechanical–biological treatment plant with front-end denitrification and phosphorus removal by simultaneous precipitation, with a maximum capacity of 640,000 PE. Within the studied period, mean and median discharges were 91,000 m^3^/day and 88,000 m^3^/day (excluding rain events), respectively, with a hydraulic retention time of about 35 h. The flow rate of the WWTP effluent in the studied period is shown in Figure S1. Following secondary treatment, conventional WWTP performance demonstrated a total removal efficiency of 99.0% for BOD_5_, 96.6% for COD, 86.3% for N-total, 98.3% for N–NH_4_, and 93.2% for P-total in 2022.^16^

### 2.3 Setup of Pilot Quaternary Treatment Technologies

The WWTP effluent was pumped into a 5 m^3^ water storage tank. In this tank, wastewater was collected and then directed to separate containers with installed technologies for further quaternary treatment (Figure S2). Detailed setup of technologies for every container is included in Figure S3. Pilot technologies were used either individually (GAC, UF) or in various combinations to achieve the required REs of pollutants. In the present study, we investigated REs for the following six combinations of technologies:

(1) GAC (granular activated carbon), outlet 800 L/h.
(2) GAC + UV radiation, outlet 800 L/h.
(3) UF (ultrafiltration), outlet 2000 L/h.
(4) UF + O_3_ (ozonation), outlet 2000 L/h.
(5) UF + GAC + UV + NF (nanofiltration), outlet 500 L/h;
(6) UF + GAC + UV + RO (reverse osmosis), outlet 500 L/h.

GAC and GAC + UV technologies were installed in container 1, where the WWTP effluent was initially filtered through a 130-μm disc filter (Azud). The next step included a treatment by GAC (Jacobi-TDS-AQUASORB-5000, particle size of 0.5–2.5 mm, iodine adsorption 1050 mg/g, surface area 1200 m^2^/g, GAC volume of 50 L, empty bed contact time of ∼5.4 min) followed by a UV low-pressure Hg lamp (Ultraviolet C-800, Aquaterm, output 65W, flow rate 2800 L/h).

UF technology was installed in container 3, where the WWTP effluent was initially treated with inline Fe_2_(SO_4_)_3_ coagulation to mitigate membrane fouling, followed by filtration through a 130-μm disc filter (Azud). UF technology consisted of hollow fiber membranes (Inge, Lenntech B.V., 1.5 mm in diameter, 0.02 μm pore size, 40 m^2^ surface area), operated in a dead-end, in-to-out mode with a constant flux of 2000 L/h. The membrane was backwashed with permeate every 30 min, and chemically enhanced backwash (with NaOH at pH 12, then with H_2_SO_4_ at pH 2.5) was applied on a schedule every 50 operational hours. UF + O_3_ samples were obtained by ozonation in container 5 of the UF-treated WWTP effluent (from container 3). UF-treated samples were ozonated using the LifeTech OZAST 100 ozone generator. In this generator, O_3_ was generated from pure oxygen through the dielectric discharge and dosed into water at 5 g/m^3^.

UF + GAC + UV + NF, and UF + GAC + UV + RO technologies were installed in container 2, where UF-treated water from container 3 flowed through GAC (Jacobi-TDS-AQUASORB-5000) and UV module (Ultraviolet C-800) to diminish the fouling potential during final nanofiltration (NF, CSM-NE4040-40 spiral wound membrane with area 7.9 m^2^, divalent ion rejection 99%) or reverse osmosis (RO, CSM RE4040-BLN, spiral wound membrane with area of 7.9 m^2^, nominal salt rejection 99.2%, low pressure). Both NF and RO membranes were operated at a constant pressure of 8 bar, cleaned with forward flushing every hour, and thoroughly Cleaned-In-Place (CIP) according to the manufacturer’s recommendations every four months. A constant flux decline has been observed between CIP procedures, but without any observable seasonal influence.

This study was initiated on 16 March 2021 and continued until 29 March 2022. To examine seasonal variability in PhACs occurrence and potential temperature influences, we partitioned data from the sampling year into a cold season (16 March 2021 – 25 May 2021, and 30 October 2021 – 29 March 2022) with WWTP effluent temperatures up to 17.6 °C (mean temperature 15.0 °C) and a warm season (26 May 2021 – 29 October 2021) with effluent temperatures above 17.6 °C (mean temperature 20.5 °C). This boundary temperature was selected as a half temperature between the low plateau temperature of colder periods (∼13.7 °C) and the high plateau temperature of a warmer period (∼21.5 °C, Figure S4). The number of samples collected in both seasons is shown in Table S1.

### 2.4 Water Sampling and Extraction

We selected 12 PhACs for study based on environmental occurrence information for European freshwater systems (Table 1). Water samples were collected, transferred to the freezer within 2 h of sampling, and stored at −20 °C for up to 3 days before processing by solid-phase extraction (SPE). Samples were collected and stored in 1 L PET bottles, which were experimentally confirmed not to influence PhAC content (Table S3). After thawing at room temperature, internal standards in MeOH (10 μL) were added to 250 mL of samples, which were subsequently extracted with MeOH-activated Oasis HLB (500 mg, 12 cc) cartridges (Waters, Milford, MA, USA). Analytes were eluted with 6 mL of MeOH/acetone (75:25) and dried up with a stream of nitrogen, and the residues were resuspended in 250 μL of 20% MeOH. The resuspended samples were filtered through centrifugal cellulose acetate filters (0.22 μm) at 1000 g for 3 min. Filtered extracts were stored at −20 °C until HPLC-MS/MS analysis.

### 2.5 Chemical Analysis

Analytical standards were purchased from Sigma-Aldrich (St. Louis, MO, USA), LGC (Teddington, UK), Absolute Standards Inc. (Hamden, CT, USA), and TRC-Canada (Toronto, Canada). Working solutions were prepared by appropriate dilution in methanol. LC-MS-grade methanol was purchased from Biosolve b. v. (Valkenswaard, The Netherlands). Water was purified using a Milli-Q Water System (Millipore Corp., Bedford, MA, USA). Ammonium acetate and formic acid (≥98.0%) were obtained from Fisher Scientific, UK.

For separation and detection of pharmaceuticals, ultra-performance liquid chromatography (UPLC Acquity, Waters, Milford, MA, USA) coupled to a triple quadrupole mass spectrometer Xevo TQS (Waters, Milford, MA, USA) with electrospray in both negative and positive ionization modes was used.^22^ Detailed information on analytical procedures is shown in Table S3.

Quantification of target analytes used external calibration with a range of 0.5–500 ng/mL with correction on mass-labeled internal standards (sulfamethoxazole d4, acetaminophen d4, atenolol d7, carbamazepine 13C6, and Ibuprofen d3, Table S4). Two different chromatographic methods were used for pharmaceutical analysis. Analytes were separated on an ACQUITY BEH C18 column (100 × 2.1 mm, 1.7 μm) with mobile phases containing 0.01% formic acid and 1 mM ammonium acetate in both methanol (B) and water (A). The flow rate for the first method was 0.35 mL/min (60 °C), and gradient elution increased from 10% to 100% B in 5 min for an ESI + - operated mass spectrometer. For the second method, the flow rate was 0.2 mL/min (40 °C), and gradient elution increased from 40% to 100% of B in 7 min for ESI- operated MS. The method limit of quantification (LOQ) was calculated as 10 times the standard deviation of the procedural blank concentrations (*n* = 8; Table S3). Recovery of PhACs was in the acceptable range of 73–115% (Table S4).

### 2.6 Quality Assurance and Quality Control (QA/QC)

QA/QC measures were implemented throughout the analysis of water samples, incorporating repeated extractions of the blanks and spiked samples. To assess the potential contamination introduced during sample storage, extraction, and analysis, distilled water blanks (*N* = 8) in plastic storage bottles were employed. The median concentration of positive procedural blanks was subtracted from the results for the analytes erythromycin and ketoprofen (Table S4). QC samples prepared at concentrations of 5 and 50 ng/mL were run after every 20 extracts, confirming acceptable repeatability with relative standard deviations (RSDs) not exceeding 15%.

### 2.7 Analysis of Difference

Analytical results were examined statistically using SigmaPlot for Windows 15 (Grafiti LLC, Palo Alto, CA, USA) or GraphPad Prism 7 for Windows (GraphPad Software, Boston, MA, USA). Data were tested for normality of variance before testing for potential differences (α = 0.05). Student’s *t* test was used to compare PhAC concentrations between the cold and warm seasons and between GAC and GAC + UV. A paired *t* test was used to compare PhAC concentrations in the WWTP effluent and subsequent QTTs, where samples used for the paired *t* test were sampled on the same day. A number of samples taken from particular technologies are shown in Table S 1.

### 2.8 Probabilistic Environmental Hazard Assessment

PhAC concentrations in water samples were fitted to a log-normal distribution to create environmental exposure distributions (EEDs) for subsequent probabilistic environmental hazard assessments (PEHAs) by PhAC and QTT following previously reported methods.^7–12,23–27^ All graphs were prepared with SigmaPlot for Windows 15. Concentration data were ranked in ascending order, and a percent rank for each concentration was calculated using the Weibull formula: *j* = (*i* * 100)/(*n* + 1), where *i* is the numerical rank assigned for each concentration, and *n* is the number of sampling events. The EEDs were constructed by plotting concentrations (log-normal scale) of PhAC measured in WWTPeff. or QTT-treated water samples against their respective percent rank (probability scale); EEDs thus indicate the probability (axis y) of a given PhAC concentration (axis x) to be met or exceeded (e.g., PNEC or PNEC-AR values). Linear regression analysis was performed, and centile values were calculated following the equation: Centile value = NORMDIST­((*a* * log_10_(*x*)) + *y*_0_), where NORMDIST returns the probability of getting less than or equal to a particular value in a normal distribution, and *a* and *y*_0_ represent the slope and intercept, respectively. Regression analysis was performed for technologies for which at least 6 samples were available, and at least 80% of the samples were above LOQ. PEHAs were then performed using EEDs, and PNEC values for ecotoxicology data, and PNEC-AR values for antibiotic resistance (Table 1). This comparison is expressed as a predicted percent exceedance (PPE).

## 3 Results and Discussion

### 3.1 Seasonal Variability of PhACs in WWTP Effluent Discharge

Here, we analyzed the quality of WWTP effluent discharge for the presence of PhACs in surface waters, particularly because effluent-dominated and dependent receiving systems may represent worst-case scenarios for exposure to PhACs and other down-the-drain chemicals.^28,29^ In fact, such effluent-dominated and dependent systems may present ideal locations^30^ to examine potential exceedances of earth system boundaries for novel entities.^31^ In the current study, the WWTP effluent discharge we examined here is usually diluted by the receiving system to represent 33% of the downstream river flow.^32^ As discussed below, even after such dilution, we identified several PhACs that pose environmental risks, indicating that the use of QTTs will be necessary to meet the European 80% indicator PhAC reduction goal.^4^

All analyzed WWTP effluent samples (19 and 18 in the cold and warm seasons, respectively) contained 10 of the targeted PhACs above the LOQ. The remaining two compounds (acetaminophen and ibuprofen) were detected in most but not all of the analyzed samples (Figure 2). The sum concentration of all determined PhACs (ΣPhACs) ranged from 2298 to 7159 ng/L with a mean value of 4373 ng/L during the cold season and ranged from 2341 to 6500 ng/L with a mean value of 4367 ng/L in the warm season. Effluent concentrations of these PhACs were similar to previous results by other authors who studied this WWTP.^33^ Abundance of individual PhACs differed over the studied period (Figures 1 and S7); however, there was no significant difference in ΣPhACs between the cold and warm seasons (*p* = 0.963; Figure 2) and no correlation with the temperature of the WWTP effluent (Figure S6). Three individual PhACs, trimethoprim, atenolol, and ibuprofen, were significantly lower (*p* ≤ 0.01) in the warm season (Figure 2B–D, and correlation analysis in Figure S6). Diclofenac and ketoprofen were significantly higher (*p* ≤ 0.05) in the warm season (Figure 2E,F); however, they exhibited weak correlations of 0.263 and 0.382, respectively (Figure S6).

**Figure 1:**
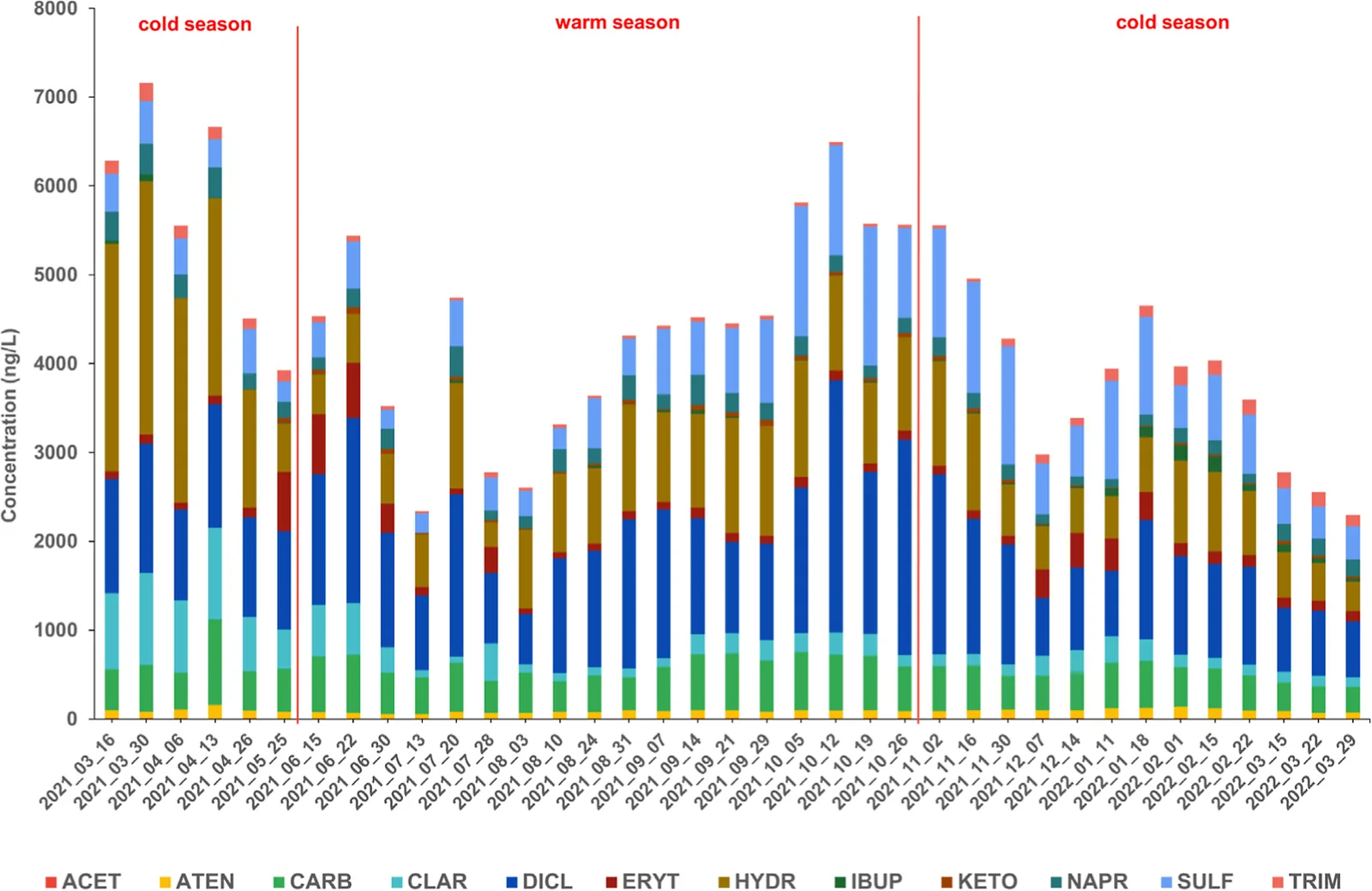
Sum (ng/L) of 12 pharmaceutically active compounds (PhACs) and their distribution in the wastewater treatment plant effluent during cold and warm seasons over a one-year study period. Detailed occurrence patterns of all PhACs in effluent samples are provided in Figure S7. The acronyms for pharmaceuticals are defined in Table 1.

**Figure 2:**
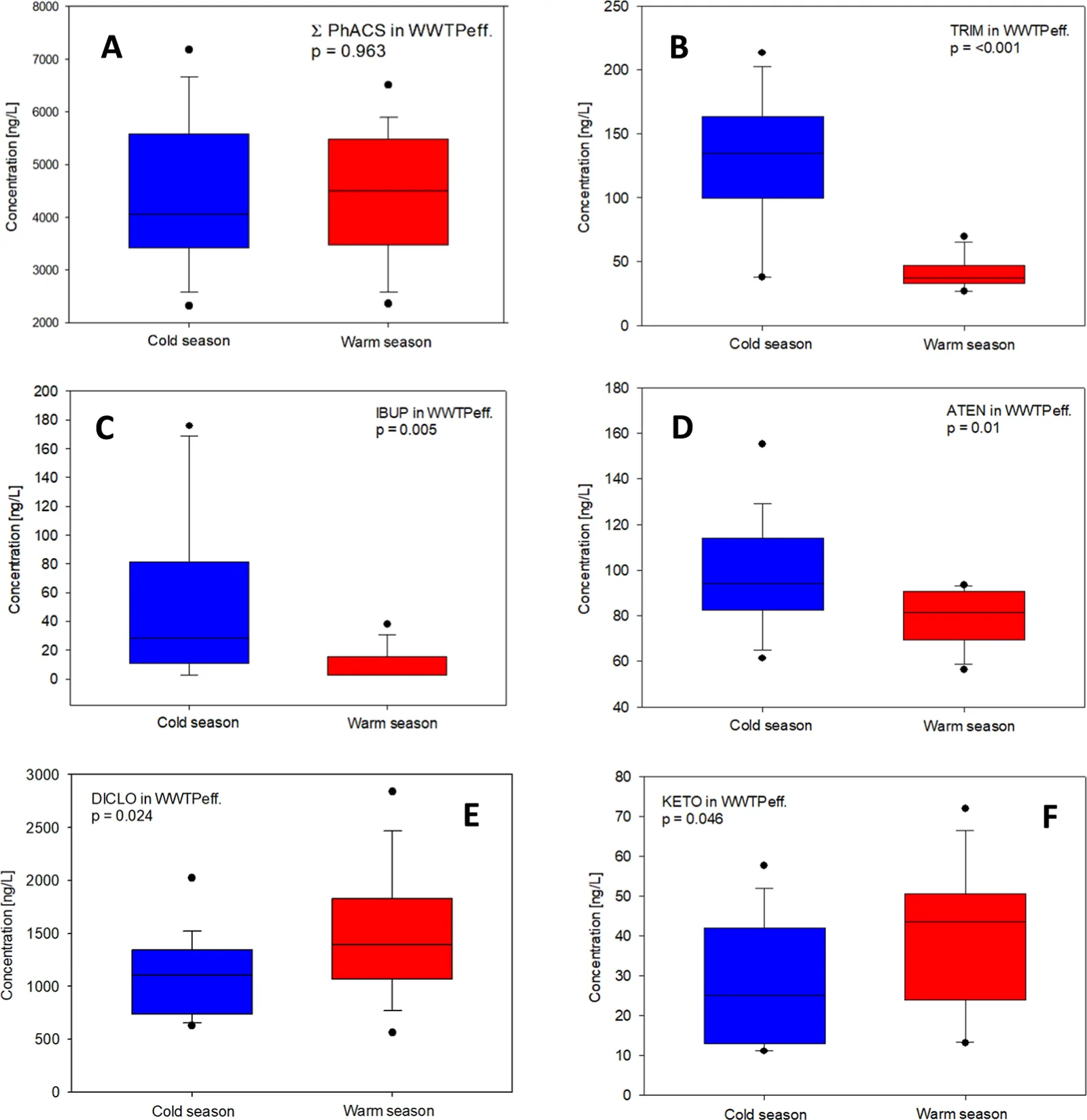
Sum (ng/L) of pharmaceutical active compounds (PhACs, A), and five different pharmaceuticals with significantly higher (*p* < 0.05) concentrations in cold seasons (trimethoprim, ibuprofen, and atenolol; B–D), or significantly higher (*p* < 0.05) concentrations in warm season (diclofenac and ketoprofen; E and F), measured in wastewater treatment plant effluent during a one-year study period. Box plots summarize results from 19 and 18 samples from the cold and warm seasons, respectively. The boxes define the 25th and 75th percentiles, with a line at the median; error bars indicate the 10th and 90th percentiles, and outlying data points are shown. Results for remaining PhACs without a significant difference in concentrations between seasons (*p* > 0.05) are in Figure S7.

Though this study of QTTs was not designed to identify factors influencing PhAC concentrations in the WWTP effluent, several factors should be considered. First, there could be significant differences in RE of many PhACs between cold and warm seasons under the Central European climate.^34,35^ In the present study, the mean daily high temperature during the cold season was 15.0 °C, and in the warm season, it was 20.5 °C, which may have affected the efficiency of biological treatment processes.^36,37^ Degradation of PhACs in the aquatic environment (including primary and secondary wastewater treatment) can be influenced by temperature and photoperiod, so greater elimination at least for some PhAC may be expected to occur during warmer periods.^38^

Consumption of some pharmaceuticals varies among seasons,^36,39,40^ which could have influenced observations reported in the current study. For example, Švecová et al. (2021) observed elevated levels of selected PhACs and surface water quality hazards during cold months, despite higher instream dilution of effluent discharges, when colds and flu were elevated in Czechia.^41^ In our study, significantly higher (*p* < 0.05) concentrations of the antibiotic trimethoprim, antihypertensive atenolol, and NSAID ibuprofen were observed in the study during the cold season, while NSAIDs diclofenac and ketoprofen (*p* < 0.05) and also anticonvulsant carbamazepine (*p* = 0.056) were detected at elevated levels during the warm season (Figures 2 and S7). It is also important to note that our study was initiated during the global COVID-19 pandemic, when a complete lockdown of Czechia occurred in March and April 2021, which also could have influenced PhAC consumption patterns^42,43^ and subsequent loading in influent sewage to the WWTP examined here.

Further, the invariability of inflows to the WWTP, associated with rain events and infiltration, could have diluted PhACs in influent sewage. Over the course of this one-year study, the inflow rate ranged from 59,370 to 169,520 m^3^/day on our sampling dates, for which the median flow rate was higher in the cold seasons (Figure S8), and there were greater extremes of the flow rate during the warm season (Figure S1). However, the inflow rate was not statistically related to the concentration of ΣPhACs in the WWTP effluent (*p* = 0.27, Figure S9).^44^

### 3.2 Seasonal Variability of PhACs Removal by QTTs

QTT REs of PhACs in both cold and warm seasons of the year are shown in Figures 3 and 4 and Table 1. UF was the least efficient QTT, as it did not significantly (*p* > 0.05) decrease ΣPhACs in the WWTP effluent. GAC treatment significantly (*p* < 0.05) decreased ΣPhACs in the WWTP effluent by 36.4 and 19.5% in cold and warm seasons, respectively. GAC + UV significantly (*p* < 0.05) removed ΣPhACs of 41.7 and 47.3% in cold and warm seasons, respectively, which was caused mainly by UV degradation of diclofenac, ketoprofen, and sulfamethoxazole (Figure 4). Since both diclofenac and sulfamethoxazole were among the most abundant PhACs in the WWTP effluent (Table S5), UV resulted in a significant (*p* < 0.05) decrease of ΣPhACs by the combined GAC + UV technology to ∼50% (i.e., 2334 ng/L) of the original effluent levels (Figure 3).

**Figure 3:**
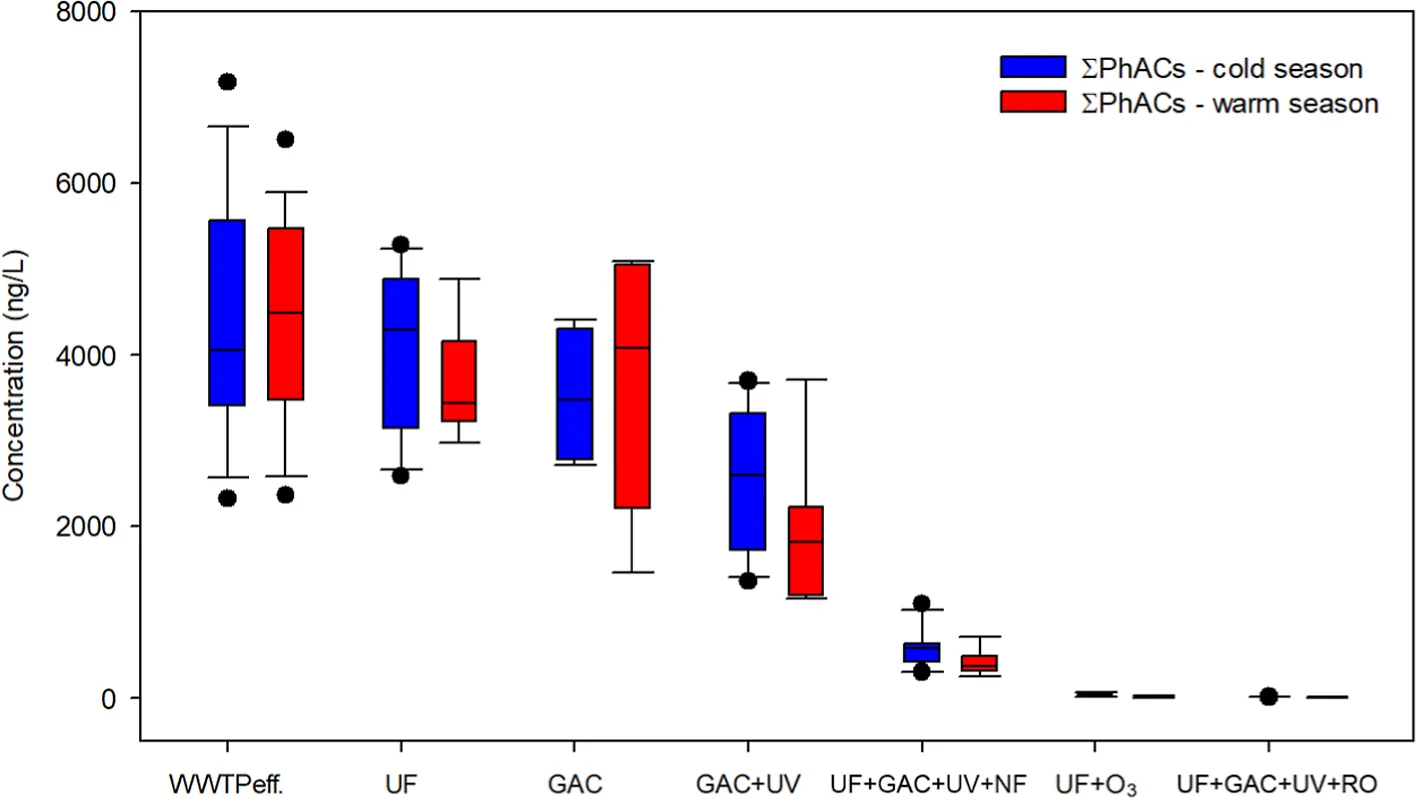
Sum (ng/L) of pharmaceutically active compounds (ΣPhACs) in the wastewater treatment plant effluent (WWTPeff.), and ΣPhACs after treatment by six different quaternary treatment technologies (QTTs). Box plots summarize results from all samples from the cold and warm seasons of the studied period, respectively. Analysis of differences between QTTs and WWTPeff. was not performed because the number of samples varied across technologies (Table S1), which makes a paired *t* test inappropriate. The boxes define the 25th and 75th percentiles, with a line at the median; error bars are the 10th and 90th percentiles and outlying points.

**Figure 4:**
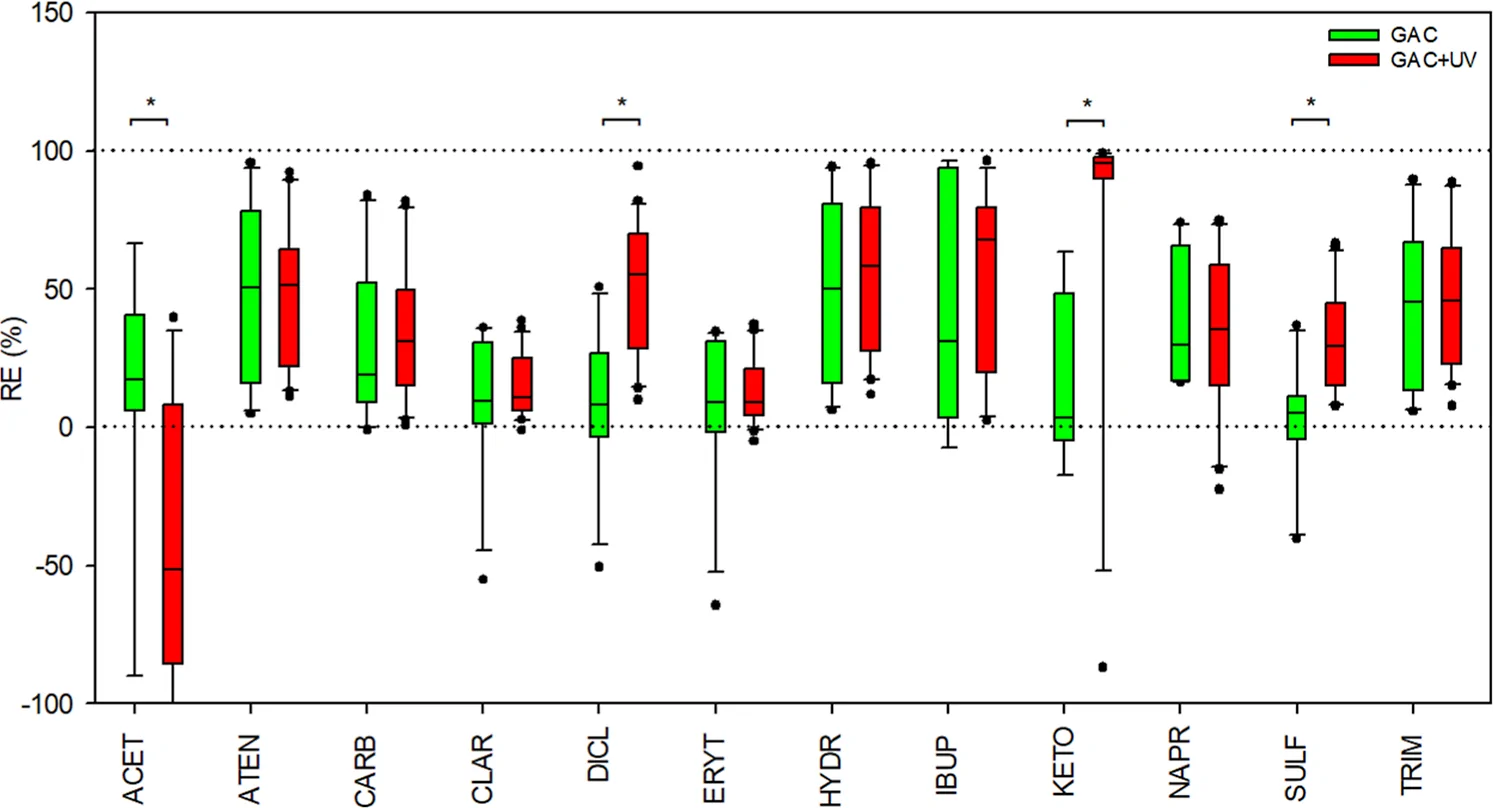
Comparison of removal efficiencies (RE) of individual pharmaceuticals from the wastewater treatment plant effluent by granular activated carbon (GAC; green) or GAC + ultraviolet radiation (UV; red) QTTs. Box plots summarize results from both the cold and warm seasons. The boxes define the 25th and 75th percentiles, with a line at the median; error bars indicate the 10th and 90th percentiles, and outlying points are shown. * denotes significant differences (*p* < 0.05) between GAC and GAC + UV treatments.

Interestingly, acetaminophen significantly (*p* < 0.05) increased in the GAC + UV-treated samples due to UV radiation (Figure 4 and Table 2). This may have resulted from the enrichment of parent acetaminophen if UV treatment deconjugated acetaminophen metabolites in the WWTP effluent. Interestingly, the conjugated forms (mainly acetaminophen-glucuronide and acetaminophen-sulfate) of this medicine excreted by humans following use can be found in rivers at similar or at greater levels than parent compounds^45,46^ rivers at similar or at greater levels than parent compounds.^45,46^

NF (in the UF + GAC + UV + NF setup) significantly (*p* < 0.05) decreased ΣPhACs by >88.6% to <499 ng/L. NF removed most PhACs in both seasons with an efficiency of >90%, except for acetaminophen, carbamazepine, hydrochlorothiazide, and sulfamethoxazole (Tables 2 and S9). These four PhACs remained in NF-treated wastewater at median concentrations of 2.2 and 1.3 ng/L, 69.1 and 66.4 ng/L, 219.0 and 143.4 ng/L, and 145.0 and 64.4 ng/L in the cold and warm seasons, respectively. Relatively lower removal efficiencies for acetaminophen, carbamazepine, and hydrochlorothiazide may have been influenced by their lower K_OW_ values (Table 1);^47^ however, membrane rejection can also be influenced by other parameters such as water solubility, size of the molecule, charge, or electrostatic interactions.^48^ Lower-than-expected NF-RE for carbamazepine (28%) and sulfamethoxazole (75%) were also reported by García-Vaquero et al. (2014).^49^ In addition, Xu et al. (2020) observed NF-RE of carbamazepine (77.6% and 90.7%) and sulfamethoxazole (>85% and 100%), in the warm and cold seasons, respectively.^50^ These authors attributed the higher rejection rates observed during the cold season to a temperature-induced reduction in membrane pore size, thereby enhancing the effectiveness of NF systems in removing larger PhACs. Notably, both studies applied NF for effluent treatment directly following conventional wastewater treatment.

Ozone (in the UF + O_3_ setup) and RO (in the UF + GAC + UV + RO setup) decreased the ΣPhACs concentration by > 99.4 and >99.7% to <27.4 and <12.6 ng/L, respectively (both seasons combined). In fact, most compounds were undetected in ozone- and RO-treated samples (<LOQ, Tables S10 and S11); thus, we used 1/2LOQ for non-detected compounds in RE calculations (Table 2). In several of the RO-treated samples, only residues (lower units of ng/L) of initially the most abundant PhACs were detected (diclofenac, hydrochlorothiazide, naproxen, and sulfamethoxazole). Relatively low mean removal efficiency for acetaminophen >92.9 and >78.3, in cold and warm seasons, and >93.8% for ibuprofen in the cold season (Table 2) may be related to generally low concentrations of these compounds within the WWTP effluent prior to QTT (concentrations near the LOQ, Table S5). After QTT, most compounds were < LOQ, and calculations using 1/2LOQ values resulted in numerically low removal efficiencies. This also applied to ozone treatment, where most compounds were removed to < LOQ. Interestingly, ketoprofen and ibuprofen appeared to be relatively resistant to ozone degradation. Ketoprofen decreased by >91.6% and >94.5% in the cold and warm seasons, respectively, and ibuprofen decreased by >81.4% in the cold season. Still, in the warm season, ibuprofen was reduced to < LOQ in all samples (Table S10). Previously, Nakada et al. (2007) reported the treatment of ketoprofen with ozone, with RE ranging from 52% to 93% across four sampling campaigns.^51^ Although sampling in this previous study was conducted in both the cold and warm seasons, there was no indication of a relationship between temperature variation and the removal of the compound. In the same study, ibuprofen exhibited highly variable RE values ranging from −156% to 75.7%. This variability was hypothesized to result from the extremely low concentrations of the pharmaceutical entering the ozonation process (near the LOQ), along with differences in reactivity between ibuprofen’s chemical structure and oxidation by ozone or hydroxyl radicals.^51^

The UWWTD requires an average RE for indicator PhACs to be > 80 or for plants to be 50,000 PE by 2045.^4^ Thus, we have noted an 80% RE threshold in Table 2 (numbers in italics) to illustrate the worst-case scenario—the case in which the secondary treatment would have zero RE, and fulfilling the 80% RE requirement would rely solely on QTTs. Of particular relevance to the indicator PhACs in the UWWTD, we examined carbamazepine, diclofenac, hydrochlorothiazide, and clarithromycin in the current study. Only UF + O_3_ and UF + GAC + UV + RO removed all of these indicator PhACs efficiently, while UF + GAC + UV + NF removed three of them, but hydrochlorothiazide with REs of only 61 and 66% during cold and warm seasons, respectively (Table 2). It is important to note that the goal in the UWWTD for >80% removal is complete wastewater treatment, including secondary treatment, which can significantly reduce PhACs in influent sewage. However, as indicated here and elsewhere, secondary treatment may not reach the desired 80% REs for currently prioritized PhACs and others in the future. For example, Molnarova et al. (2024) reported a mean secondary treatment removal of 45% for hydrochlorothiazide across 10 Czech WWTPs.^14^ In our present study, we identified REs for hydrochlorothiazide in WWTP effluent with 61 and 66% REs by UF + GAC + UV + NF; thus, adding QTT REs from our research to secondary treatment from the literature yields 78.5 and 81.3% overall REs for hydrochlorothiazide in the cold and warm seasons, respectively.

NF, RO, and ozonation proved to be the most effective QTTs; however, these technologies are among the most expensive of the studied QTTs and also pose other drawbacks. Modern NF and RO systems produce 15 to 25% retentate,^52,53^ which contains highly concentrated pollutants from wastewater, and its disposal, especially in land-locked countries, is problematic. Ozonation has the advantage that it does not produce any secondary waste streams; however, it produces potentially toxic transformation byproducts, including bromate, a probable human carcinogen.^54^ To minimize health and environmental risks from wastewater ozone treatment, bromate control strategies should be considered.^55^

None of the remaining QTTs (UF, GAC, and GAC + UV) reached a ΣPhACs RE of >80%. However, data from the literature suggest that >80% RE is possible for the majority of the PhACs examined here using GAC and GAC + UV (Table 2, third column for each QTTs). Much lower REs for GAC and GAC + UV in our research may have resulted from a lower mass of GAC applied, with correspondingly shorter breakthrough times. For example, Knytl et al. (2024) used 800 L of GAC with a breakthrough volume of 2000 m^3^, which covered their entire experimental period of 52 days with an RE of >80%,^56^ while we used only 50 L of similar quality GAC with a calculated breakthrough operating time of only ca. 5 days. We exchanged GAC only three times during the year-long study period. After placement of the new GAC (marked with an arrow in Figure S 11), REs for ΣPhACs during sampling periods were higher (58% for GAC and 72% for GAC + UV, respectively) compared to the mean REs of 28% and 45% for GAC and GAC + UV, respectively, over the year-long study. These results suggest that a significant part of our year-long study did not measure the adsorption performance of GAC, but only the behavior of spent carbon.

### 3.3 Exceedances of Water Quality Values

WWTP effluents are usually discharged directly to surface water receiving systems or may be beneficially reused for potable source water, agriculture (including aquaculture), industrial operations, or other purposes. Thus, it is important to understand the environmental hazards and risks presented by residual PhACs to aquatic organisms following the quaternary treatment approach. Currently, environmental assessment for PhACs most commonly relies on a deterministic approach and risk quotients, which assume a single exposure level to account for exposure. In contrast, probabilistic approaches, including PEHAs using EEDs, are advantageous because they can predict the likelihood of encountering specific concentrations of interest in the environment. In the present study, we constructed probabilistic EEDs for all measured PhACs in both the WWTP effluent and the quaternary treated effluent during both cold and warm seasons (Figures 5, 6, and S10), and then we performed PEHAs with the ecotoxicological data, i.e., PNEC for all compounds and PNEC-AR for antibiotics (Tables 3, 4, and S12). Finally, we compared the aquatic hazard of the studied compounds among WWTP effluents and different QTTs. As noted above, if a PhAC concentration was < LOQ, it was replaced with a 1/2LOQ value and thus considered a potential worst-case scenario. To perform regression analysis and PEHA, at least 80% of samples were above LOQ, and at least 6 samples for a particular QTT were available (not fulfilled for GAC in the cold season).

**Figure 5:**
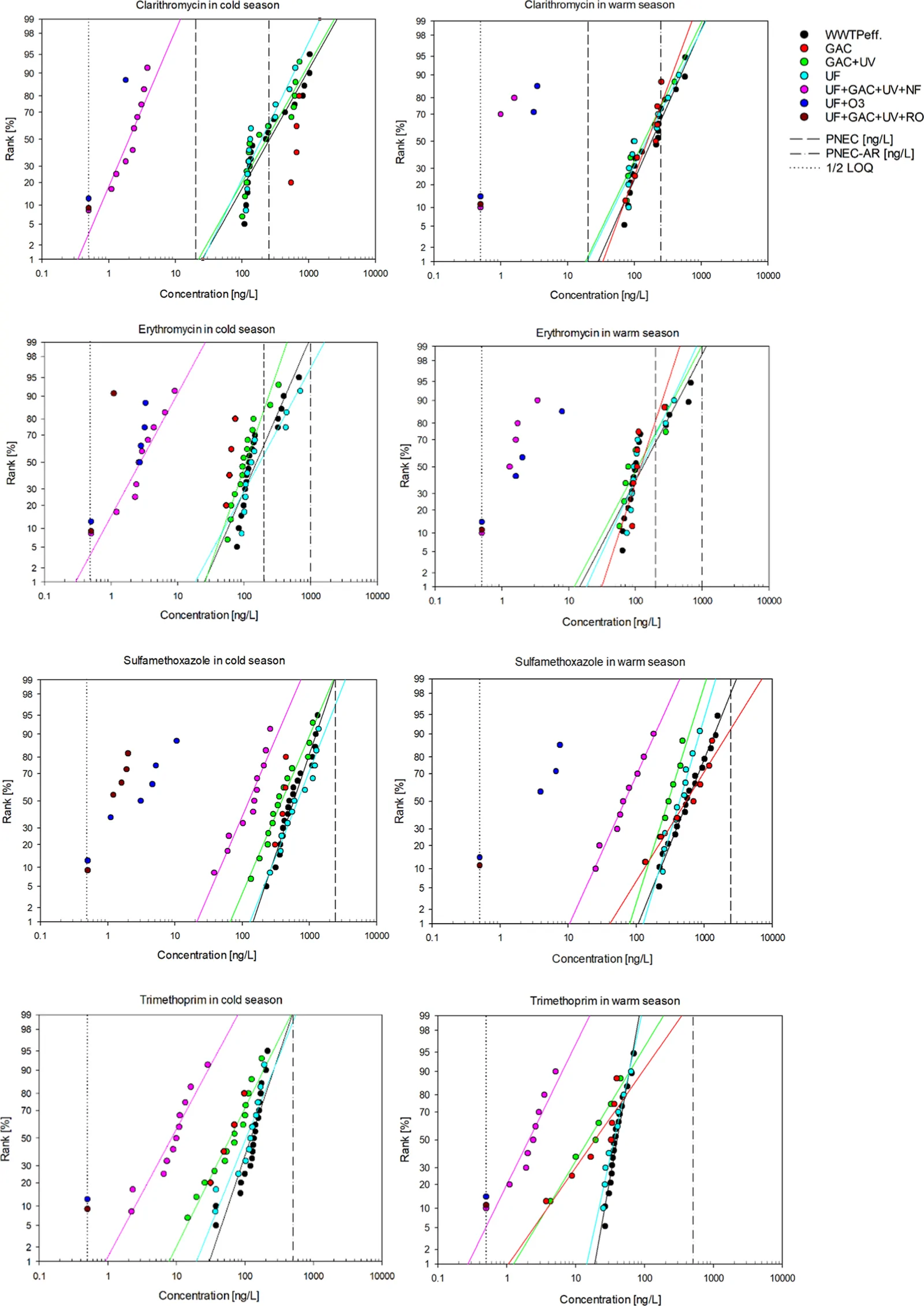
Environmental exposure distributions (EED) used for probabilistic environmental hazard assessments for the antibiotics clarithromycin, erythromycin, sulfamethoxazole, and trimethoprim during cold season (left column) and warm season (right column). Vertical dash lines represent the PNEC values, and the vertical dash-dotted line represents the PNEC-AR values (Table 1). Characterization of regression curves and calculation of predicted percent exceedance (PPE) values are shown in Table 3. The dotted line represents 1/2LOQ (0.5 ng/L). Samples with concentrations < LOQ are aggregated at a single point at 1/2LOQ without a regression curve, which is constructed from at least 6 samples and at least 80% of samples above LOQ.

**Figure 6:**
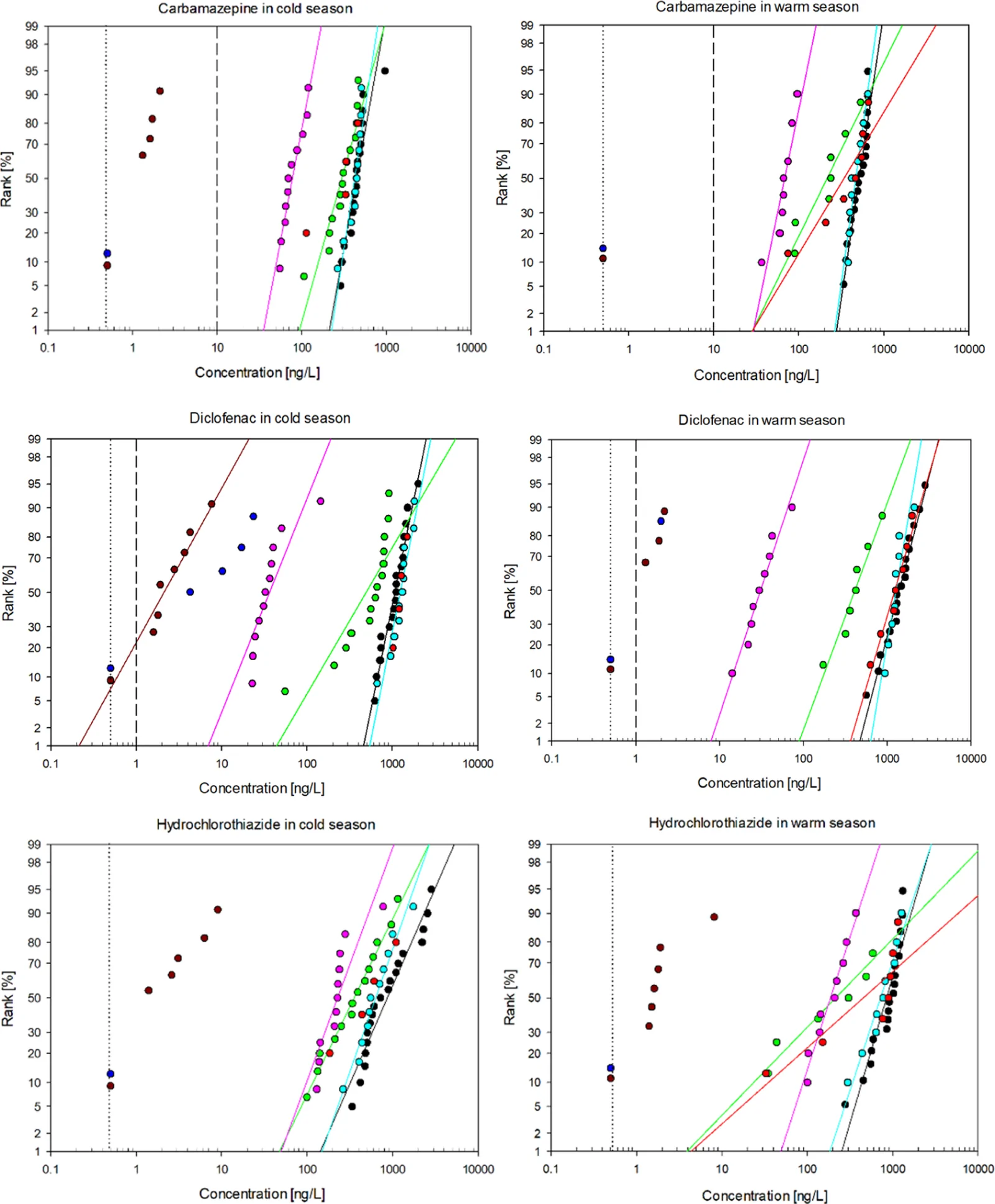
Environmental exposure distributions (EED) used for probabilistic environmental hazard assessments for the pharmaceuticals carbamazepine, diclofenac, and hydrochlorothiazide during cold season (left column) and warm season (right column). Vertical dash lines represent the PNEC values (Table 1). Characterization of regression curves and calculation of predicted percent exceedance (PPE) values are shown in Table 4. The dotted line represents 1/2LOQ (0.5 ng/L). Samples with concentrations < LOQ are aggregated at a single point at 1/2LOQ without a regression curve, which is constructed from at least 6 samples, and at least 80% of samples above LOQ. PhACs with PPE of 0% or without regression curves in EED graphs (acetaminophen, atenolol, ibuprofen, ketoprofen, and naproxen) are shown in Figure S10 and Table S12.

**Table 3:** Equations for the Regression Line and Values Corresponding to Various Centile Values for Antibiotics EEDs in Figure 5 of the Reported Determined Environmental Concentration in WWTPeff., and WWTPeff. Samples Treated with QTTs^a^

compound	season	technology	*n*	r2	slope	intercept	centile value (ng/L)									PPE of PNEC	PPE of PNEC-AR
							1	5	10	25	50	75	90	95	99	(%)	(%)
CLAR	cold	WWTPeff.	19	0.87	2.30	–5.54	25.1	49.7	71.5	131.4	258.2	507.7	932.9	1342.7	2658.4	99.5	51.3
		GAC + UV	14	0.83	2.28	–5.39	22.1	44.0	63.5	117.3	232.0	458.7	847.4	1223.5	2436.7	99.2	47.0
		UF	11	0.79	2.65	–6.07	25.8	46.7	64.0	108.4	194.8	349.8	592.7	812.5	1468.4	99.6	38.7
		UF + GAC + UV + NF	11	0.86	3.03	–0.91	0.3	0.6	0.8	1.2	2.0	3.3	5.3	7.0	11.7	38.7	0.0
	warm	WWTPeff.	18	0.93	2.91	–6.55	28.3	48.6	64.8	104.8	178.7	304.8	492.7	656.9	1126.6	99.7	33.6
		GAC	7	0.87	3.48	–7.63	33.3	52.3	66.5	99.3	155.1	242.3	361.9	460.2	722.1	99.9	23.5
		GAC + UV	7	0.88	2.66	–5.70	18.5	33.4	45.7	77.2	138.3	247.7	418.6	573.0	1032.5	98.7	24.7
		UF	9	0.87	2.62	–5.70	19.5	35.4	48.8	83.2	150.4	272.2	464.1	638.7	1162.6	98.9	28.2
		UF + GAC + UV + NF	9	0.97	4.41	0.08	0.3	0.4	0.5	0.7	1.0	1.4	1.9	2.3	3.2	0.0	0.0
ERYT	cold	WWTPeff.	19	0.83	2.98	–6.50	25.2	42.7	56.6	90.5	152.5	256.9	410.9	544.2	921.8	36.3	0.8
		GAC + UV	14	0.91	3.76	–7.62	25.5	38.7	48.3	70.0	105.8	159.9	231.8	289.5	439.3	14.9	0.0
		UF	11	0.79	2.40	–5.35	18.3	35.2	49.9	89.4	171.0	326.9	585.8	830.6	1598.7	43.5	3.3
		UF + GAC + UV + NF	11	0.91	2.38	–1.05	0.3	0.6	0.8	1.4	2.7	5.3	9.5	13.5	26.0	0.0	0.0
	warm	WWTPeff.	18	0.78	2.45	–5.16	14.4	27.3	38.4	68.0	128.3	242.0	428.3	602.9	1144.6	31.8	1.5
		GAC	7	0.59	3.99	–8.28	31.0	46.0	56.7	80.6	118.9	175.5	249.1	307.3	455.4	18.4	0.0
		GAC + UV	7	0.82	2.43	–4.94	11.9	22.8	32.1	57.1	108.1	204.8	364.0	513.5	979.4	25.8	0.9
		UF	9	0.73	2.82	–5.87	18.2	31.7	42.7	70.2	121.9	211.6	347.7	468.0	817.2	27.2	0.5
SULF	cold	WWTPeff.	19	0.95	3.83	–10.59	143.2	215.6	268.2	386.2	579.0	868.2	1250.1	1554.9	2341.3	0.9	0.0
		GAC + UV	14	0.96	3.03	–7.83	65.8	110.5	145.7	231.1	385.9	644.5	1022.4	1347.6	2262.4	0.8	0.0
		UF	11	0.95	3.28	–9.25	130.1	210.0	271.1	415.4	667.5	1072.4	1643.3	2121.5	3425.4	3.4	0.0
		UF + GAC + UV + NF	11	0.92	3.01	–6.30	20.9	35.3	46.6	74.2	124.5	208.7	332.2	438.8	739.7	0.0	0.0
	warm	WWTPeff.	18	0.98	3.22	–8.85	105.8	172.2	223.3	344.6	558.2	904.2	1395.6	1809.5	2945.7	2.1	0.0
		GAC	7	0.95	2.08	–5.67	40.3	85.6	128.0	250.7	529.0	1116.2	2185.6	3267.6	6947.9	8.6	0.1
		GAC + UV	7	0.95	4.11	–10.13	79.0	115.8	141.9	199.4	290.9	424.3	596.2	730.7	1070.2	0.0	0.0
		UF	9	0.96	4.39	–11.55	126.5	180.9	218.9	301.1	429.0	611.2	840.6	1017.2	1454.7	0.1	0.0
		UF + GAC + UV + NF	9	0.98	2.87	–5.24	10.4	17.9	24.0	39.1	67.1	115.2	187.5	251.0	433.5	0.0	0.0
TRIM	cold	WWTPeff.	19	0.81	3.85	–8.02	30.1	45.3	56.3	81.0	121.2	181.5	261.0	324.3	487.6	0.0	0.9
		GAC + UV	14	0.95	2.61	–4.64	7.7	14.1	19.4	33.2	60.3	109.3	186.7	257.3	469.6	0.0	0.8
		UF	11	0.86	3.21	–6.47	19.5	31.8	41.3	63.8	103.5	167.9	259.5	336.7	549.0	0.0	1.4
		UF + GAC + UV + NF	11	0.91	2.41	–2.23	0.9	1.8	2.5	4.4	8.4	16.1	28.7	40.7	78.0	0.0	0.0
	warm	WWTPeff.	18	0.95	7.21	–11.51	18.7	23.3	26.1	31.7	39.3	48.8	59.2	66.5	82.7	0.0	0.0
		GAC	7	0.83	1.85	–2.36	1.0	2.4	3.8	8.1	18.8	43.6	92.8	145.9	340.9	0.0	0.4
		GAC + UV	7	0.98	2.14	–2.52	1.2	2.6	3.8	7.3	15.2	31.5	60.5	89.6	186.7	0.0	0.1
		UF	9	0.95	5.83	–9.07	14.3	18.7	21.6	27.4	35.8	46.7	59.4	68.5	89.7	0.0	0.0
		UF + GAC + UV + NF	9	0.90	2.64	–0.83	0.3	0.5	0.7	1.1	2.1	3.7	6.3	8.7	15.7	0.0	0.0

a Additionally, predicted percent exceedance (PPE) is given for PNEC and PNEC-AR values (Table 1).

**Table 4:** Equations for the Regression Line and Values Corresponding to Various Centile Values for Pharmaceutical EEDs in Figure 6 of the Reported Determined Environmental Concentration in WWTPeff., and WWTPeff. Samples Treated with QTTs^a^

compound	season	technology	*n*	r2	slope	intercept	centile value (ng/L)									PPE of PNEC
							1	5	10	25	50	75	90	95	99	(%)
CARB	cold	WWTPeff.	19	0.85	7.18	–19.00	209.6	260.8	293.0	356.0	442.0	548.7	666.6	748.9	931.9	100.0
		GAC + UV	14	0.88	4.64	–11.46	93.5	131.2	157.1	212.4	297.0	415.1	561.3	672.3	943.1	100.0
		UF	11	0.82	8.54	–22.37	222.9	267.9	295.5	348.1	417.6	500.9	590.1	650.8	782.2	100.0
		UF + GAC + UV + NF	11	0.93	6.80	–12.83	35.0	44.1	49.9	61.3	77.0	96.8	118.8	134.4	169.3	100.0
	warm	WWTPeff.	18	0.92	8.67	–23.46	274.1	328.4	361.7	425.0	508.4	608.1	714.5	786.9	943.1	100.0
		GAC	7	0.83	2.15	–5.43	27.9	57.9	85.5	163.8	337.5	695.5	1333.2	1967.9	4085.6	100.0
		GAC + UV	7	0.91	2.64	–6.14	28.1	50.9	69.9	118.8	214.1	386.0	656.1	901.2	1634.6	100.0
		UF	9	0.91	9.44	–25.19	264.6	312.5	341.5	396.0	466.8	550.3	638.2	697.3	823.4	100.0
		UF + GAC + UV + NF	9	0.84	6.24	–11.44	28.8	37.1	42.4	53.0	68.0	87.2	109.1	124.8	160.5	100.0
DICL	cold	WWTPeff.	19	0.96	6.29	–19.06	455.7	584.7	667.9	834.0	1067.5	1366.4	1706.3	1948.9	2500.9	100.0
		GAC + UV	14	0.75	2.22	–5.96	43.7	88.6	129.3	243.0	489.8	987.2	1855.3	2706.3	5494.9	100.0
		UF	11	0.90	6.50	–20.09	542.0	690.2	785.0	973.5	1236.5	1570.5	1947.5	2215.2	2820.6	100.0
		UF + GAC + UV + NF	11	0.76	3.24	–5.06	7.0	11.3	14.6	22.5	36.4	58.8	90.5	117.2	190.3	100.0
		UF + GAC + UV + RO	10	0.96	2.33	–0.75	0.2	0.4	0.6	1.1	2.1	4.1	7.4	10.6	20.8	77.2
	warm	WWTPeff.	18	0.97	4.95	–15.55	471.5	647.5	766.8	1017.1	1392.1	1905.4	2527.5	2993.0	4110.0	100.0
		GAC	7	0.96	4.41	–13.63	365.2	521.2	630.1	865.1	1230.2	1749.5	2401.9	2903.5	4144.2	100.0
		GAC + UV	7	0.95	3.50	–9.15	88.7	138.9	176.3	262.9	409.6	638.4	951.6	1208.5	1891.9	100.0
		UF	9	0.86	7.67	–23.83	635.5	779.8	869.6	1043.4	1277.5	1564.2	1876.8	2093.0	2568.0	100.0
		UF + GAC + UV + NF	9	0.96	3.94	–5.85	7.9	11.7	14.5	20.7	30.7	45.5	65.0	80.3	119.7	100.0
HYDR	cold	WWTPeff.	19	0.93	2.96	–8.70	141.8	240.9	319.5	512.3	865.5	1462.5	2344.8	3110.3	5284.1	0.0
		GAC + UV	14	0.99	2.66	–6.78	47.4	85.5	117.1	198.1	355.2	636.9	1077.3	1475.4	2661.4	0.0
		UF	11	0.96	3.72	–10.41	149.6	228.2	285.8	416.2	632.0	959.6	1397.6	1750.2	2669.2	0.0
		UF + GAC + UV + NF	11	0.77	3.53	–8.30	49.5	77.3	98.0	145.7	226.3	351.6	522.6	662.6	1034.1	0.0
	warm	WWTPeff.	18	0.86	4.44	–13.00	253.7	361.3	436.2	597.7	848.1	1203.5	1649.0	1991.0	2835.5	0.0
		GAC	7	0.73	1.15	–3.06	4.3	16.9	34.9	117.1	450.1	1729.9	5811.3	12000.7	46770.9	0.8
		GAC + UV	7	0.95	1.33	–3.11	3.9	12.5	23.4	66.8	213.9	685.1	1953.4	3656.8	11855.5	0.1
		UF	9	0.94	3.93	–11.22	183.7	273.9	338.9	483.8	718.6	1067.2	1523.5	1885.2	2811.2	0.0
		UF + GAC + UV + NF	9	0.97	4.00	–9.08	48.8	72.3	89.1	126.3	186.3	274.6	389.4	479.9	710.4	0.0

a Additionally, predicted percent exceedance (PPE) is given for PNEC values (Table 1).

In the current study, we identified the predicted percent exceedance (PPE) of PNECs and PNECs-AR higher than zero for all studied antibiotics (clarithromycin, erythromycin, sulfamethoxazole, and trimethoprim; Figure 5 and Table 3) and carbamazepine, diclofenac, and hydrochlorothiazide (only PNECs; Figure 6 and Table 4). Remaining EEDs for PhACs with zero PPE for all technologies are shown in Figure S10 and Table S12. Obtained PPE values consider potential worst-case scenarios in developed countries, such as the discharge of either secondary or quaternary treated wastewaters to effluent-dominated and dependent surface waters. As noted above, the studied WWTP discharged a secondary-treated effluent of 91,000 m^3^/day to the recipient Svratka River (Czechia) with a base flow (Q_355_) of 250,000 m^3^/day in the studied period.^32^ The WWTP effluent thus contributed ∼27% of the instream flows of the Svratka River downstream of the WWTP discharge. Considering the only ∼1 dilution of the WWTP effluent, resulting river concentrations of carbamazepine and diclofenac still exceeded the PNEC value (PPE) by 100% in both seasons. In addition, the PPE for clarithromycin was 88 and 85% for PNEC and 9 and 1% for PNEC-AR during cold and warm seasons, respectively (calculated from regression equations in Tables 3 and 4).

UF + O_3_ and UF + GAC + UV + RO reached the desired PPE of 0% for the majority of PhACs (Table S12), including clarithromycin and erythromycin (Figure 5). Such removal of antibiotics, including WHO essential medicines, to levels below PNEC-AR values is necessary, given the identification of antibiotic discharges exceeding an Earth system boundary for novel entities.^57^ A number of timely research questions were recently identified for future study of antibiotics in aquatic systems.^58,59^ However, diclofenac remained in environmentally relevant concentrations exceeding a PNEC value in both UF + O_3_ and UF + GAC + UV + RO-treated WWTP effluent in both seasons, and ibuprofen also exceeded the PNEC value in UF + O_3_-treated WWTP effluent during the cold season (Figure 6 and Table S12). A greater likelihood of potential environmental impact of diclofenac and ibuprofen in QTT-treated wastewater during the cold season might be due to the combination of higher concentrations of ibuprofen in the initial WWTP effluent and/or a lower RE of diclofenac during the cold season (Table 2). Diclofenac exceeded a PNEC of 1 ng/L over 50% of the time, even when treated by the most effective QTTs UF + O_3_ and UF + GAC + UV + RO (Figure 6 and Table 4). In European surface waters, diclofenac has been identified among other PhACs as presenting relatively high potential environmental risk, which results from the detected environmental concentrations, frequency of occurrence, and chronic toxicity of diclofenac to non-target aquatic organisms, such as algae (phototrophic level), *Daphnia magna* (invertebrates), and fish (vertebrates).^5^ However, some other studies recommend higher PNEC values for diclofenac. Tousova et al. (2017) reported a NORMAN PNEC value of 10 ng/L for diclofenac, which was determined based on experimental data, existing environmental quality standards (EQSs), or in silico predictions.^60^ Additionally, Leverett et al. (2021) argued that the EQS value of 40 ng/L for diclofenac derived from a single mesocosm test result and an assessment factor may be inaccurate.^61^ They suggest an evidence-driven EQS of 126 ng/L for diclofenac, based on a probabilistic (species-sensitivity distribution) approach that accounted for available data at the time.^61^ When considering the EQS diclofenac PNEC value of 126 ng/L, wastewater treated with UF + O_3_, UF + GAC + UV + RO, or UF + GAC + UV + NF would meet this limit, but for GAC + UV-treated wastewater, the PNEC would be exceeded >90% of the time, and for GAC, UF, and the secondary treated wastewater effluent examined here, this EQS PNEC value would be exceeded 100% of the time.

## 4 Conclusions

Urban wastewater recycling can provide critical freshwater resources, but it requires varying levels of treatment based on fit-for-purpose reuse within a One Water context. Over a yearlong pilot study, six advanced QTTs were examined for comparative efficiency in removing selected pharmaceuticals from secondary-treated wastewater. RO and ozone treatments displayed the highest removal efficiencies; however, these additional treatments did not reduce the water quality hazards of all PhACs below the PNEC and PNEC-AR values. Our findings support the need for WWTPs to adopt effective treatment methods to comply with the EU directive on wastewater treatment and significantly reduce environmental concentrations of PhACs.

## Supplementary Material



## Notes

Effluent flow rate (Figures S1 and S8); temperature profile (Figure S4); occurrence of pharmaceuticals (Figures S5 and S7); quaternary treatment units illustrations (Figures S2 and S3); correlations of pharmaceuticals with temperature and flow rate (Figures S6 and S9); environmental exposure distributions (EEDs, Figure S10); removal efficiency by GAC and GAC + UV technologies (Figure S11); numbers of collected samples (Table S1); comparison of samplings (Table S2); MS/MS parameters of studied analytes (Table S3); recovery of analytes from distilled water (Table S4); concentrations of pharmaceuticals in cold vs warm period (Table S5) and after different technologies (Tables S6–S11); EED equations (Table S13); and summary of the removal effectiveness (Table S14) (PDF)
