# Supplementary material for: Comparative Reduction of Pharmaceutical Hazards in Wastewater among Pilot-Scale Quaternary Treatment Technologies across Seasons
Source: ACS Environ Au. 2026 Aug 1;6(5):864–78. doi: 10.1021/acsenvironau.6c00068 (PMC13602333; doi:10.1021/acsenvironau.6c00068)
Supplement: Supplementary file 1 [file vg6c00068_si_001.pdf]

Supporting information for paper:

Comparative reduction of pharmaceutical hazards in wastewater among pilot-scale quaternary treatment technologies across seasons

Bittner M.[1], Báborská L.[2], Bočková, K.[1], Ireinová, A. [1], Holba M.[2], Lev J. [2], Polášek D.[3], Bláhová, L. [1], Brooks B.W.[4], Bláha L.[1*]

[1]Masaryk University, Faculty of Science, RECETOX, Kamenice 5, 625 00 Brno, Czechia

[2]ASIO TECH, spol. s r.o., Kšírova 552/45, 619 00 Brno, Czechia

[3] AQUA PROCON, s.r.o., Palackého tř.12, 612 00 Brno, Czechia

[4] Department of Environmental Science, Center for Reservoir and Aquatic Systems Research, Baylor University, Waco, TX 76798, USA

** Corresponding author: ludek.blaha@recetox.muni.cz*

List of supporting figures

Figure S1. Flow rate of the WWTP effluent in the studied period (16 March, 2021 – 29 March 2022).

Figure S2. Pilot quaternary treatment units (C1–C5) located at a wastewater treatment plant in Brno-Modřice, Czechia. C2 and C5 utilize UF-treated wastewater from C3. Detailed setup of technologies for every container are shown in Figure S 2.

Figure S3. Technical drawing of the setup of technologies in containers 1, 2, 3, and 5.

Figure S4. Temperature of the WWTPeff. (i.e., inflow to the pilot QTT) during the studied period 16 March, 2021 – 29 March, 2022.

Figure S5. Occurrence patterns of pharmaceuticals in WWTP effluent samples over the year. Pharmaceuticals occurring in the highest concentrations show graph A with axis y ranging from 0–3,000 ng/L; medium concentrations show graph B with axis y ranging from 0–1,200 ng/L; lowest concentrations show graph C with axis y ranging from 0–250 ng/L.

Figure S6. Correlation of concentration of pharmaceuticals in WWTP effluent with the temperature of WWTP effluent during the year. ΣPhACs (the first graph), and individual PhACs in other graphs.

Figure S7. Occurence of PhACs in WWTPeff. in a cold or warm period of the year. ΣPhACs (the first graph), and individual PhACs in other graphs (ordered according to the increasing P value). The boxes define the 25th and 75th percentiles, with a line at the median; the error bars are the 10th and 90th percentiles; and outlying points are shown.

Figure S8. Flow rate of the WWTP effluent in cold and warm seasons of the studied period (16 March, 2021 – 29 March, 2022). Only the data for flow rates from the PhACs sampling days were used. The boxes define the 25th and 75th percentiles, with a line at the median; error bars are the 10th and 90th percentiles and outlying points.

Figure S9. Correlation ($p = 0.27$) of concentration of ΣPhACs in WWTPeff. with flowrate of the WWTPeff. during the year 2021.

Figure S10. Environmental exposure distributions (EED) for all PhACs in the cold season (left column) and warm season (right column). Vertical dash lines represent the PNEC value, and vertical dash-dotted lines represent the PNEC-AR value (shown in the last two columns). The dotted lines represent the ½LOQ value (2.5 ng/L for ibuprofen and 0.5 ng/L for remaining PhACs). Samples with concentrations <LOQ are aggregated at a single point at ½LOQ without a regression curve, which was constructed from at least six samples (GAC cold technology was not compliant because of only four samples), and at least 80 % of samples above LOQ (UF+GAC+UV+RO and UF+O_3 technologies were often not compliant because many samples were <LOQ).

Figure S11. Percentage removal of ΣPhACs from wastewater by GAC or GAC+UV technologies. The new GAC was created before the project began and was exchanged twice: on 2021-06-26 and 2021-10-22.

List of supporting tables

Table S1. Number of samples before quaternary treatment (WWTPeff.), and the number of samples collected after the individual QTT during the period from 16 March 2021 – 29 March, 2022 (exact sampling dates are stated in Tables S5–S11). WWTPeff. samples were taken at approximately two-to-six week intervals; corresponding QTT samples were taken less frequently because of the limited sample-processing capacity.

Table S2. Comparison of concentrations (ng/L) of PhACs in WWTPeff. sampled on 1 February, 2022, to PET bottle and a glass bottle.

Table S3. MS/MS parameters of studied PhACs.

Table S4. Recovery of PhACs from distilled water by SPE procedure and concentration of PhACs in blanks.

Table S5. Concentrations (ng/L) of PhACs in WWTPeff. in cold periods (periods 2021_03_16 – 2021_05_25 and 2021_11_02 – 2022_03_29) and warm period (2021_06_15 – 2021_10_26). Red values represent ½LOQ (non-detected compounds).

Table S6. Concentrations (ng/L) of PhACs in WWTPeff. treated by ultrafiltration (UF) sampled at cold periods of the year (periods 2021_04_26 – 2021_05_25 and 2021_11_02 – 2022_03_29) and warm period of the year (2021_06_08 – 2021_09_29). Red values represent ½LOQ (non-detected compounds).

Table S7. Concentrations (ng/L) of PhACs in WWTPeff. treated by Granular Activated Carbon (GAC) sampled at cold periods of the year (periods 2021_03_16 – 2021_04_13) and warm period of the year (2021_06_30 – 2021_10_12). Red values represent ½LOQ (non-detected compounds).

Table S8. Concentrations (ng/L) of PhACs in WWTPeff. treated by Granular Activated Carbon with UV irradiation (GAC + UV) sampled at cold periods of the year (periods 2021_03_16 – 2021_04_26 and 2021_11_02 – 2022_03_29) and warm period of the year (2021_06_30 – 2021_10_26). Red values represent ½LOQ (non-detected compounds).

Table S9. Concentrations (ng/L) of PhACs in WWTPeff. treated by Nanofiltration (NF, in the setup UF+GAC+UV+NF) sampled at cold periods of the year (periods 2021_03_30 – 2021_04_26 and 2021_11_02 – 2022_03_29) and warm period of the year (2021_05_25 – 2021_09_29). Red values represent ½LOQ (non-detected compounds).

Table S10. Concentrations (ng/L) of PhACs in WWTPeff. treated by Ozone (O_3, in the setup UF+O_3) sampled at cold periods of the year (periods 2021_11_16 – 2022_03_29) and warm period of the year (2021_06_30 – 2021_10_05). Red values represent ½LOQ (non-detected compounds).

Table S11. Concentrations (ng/L) of PhACs in WWTPeff. treated by Reverse osmosis (RO, in the setup UF+GAC+UV+RO) sampled at cold periods of the year (periods 2021_03_30 – 2021_04_26 and 2021_11_16 – 2022_03_29) and warm period of the year (2021_05_25 – 2021_10_12). Red values represent ½LOQ (non-detected compounds).

Table S12. Equations for regression line and values corresponding to various centile values for environmental exposure distribution (EEDs) of reported determined environmental concentration in WWTPeff., and WWTPeff. samples treated with quaternary treatment technologies. Additionally, predicted percent exceedance (PPE) is given for the predicted no-effect concentration (PNEC) and the predicted no-effect concentration for antibiotic resistance (PNEC-AR).

Table S13. Summary of the effectiveness of removal of PhACs by QTTs in both cold and warm seasons of the year, and corresponding data from the literature (in the third column for each QTT, literature is listed below the table).

Figure S1. Flow rate of the WWTP effluent in the studied period (16 March, 2021 – 29 March 2022).

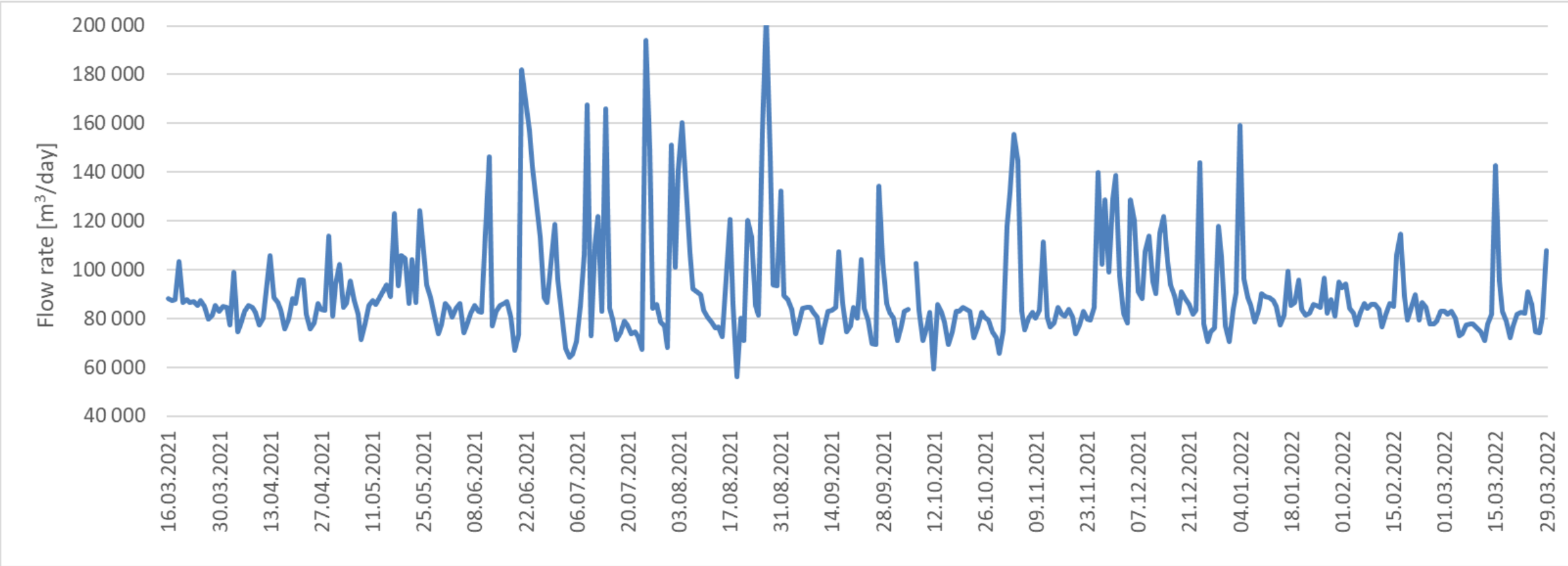

Figure S2. Pilot quaternary treatment units (C1–C5) located at a wastewater treatment plant in Brno-Modřice, Czechia. C2 and C5 utilize UF-treated wastewater from C3. Detailed setup of technologies for every container are shown in Figure S 2.

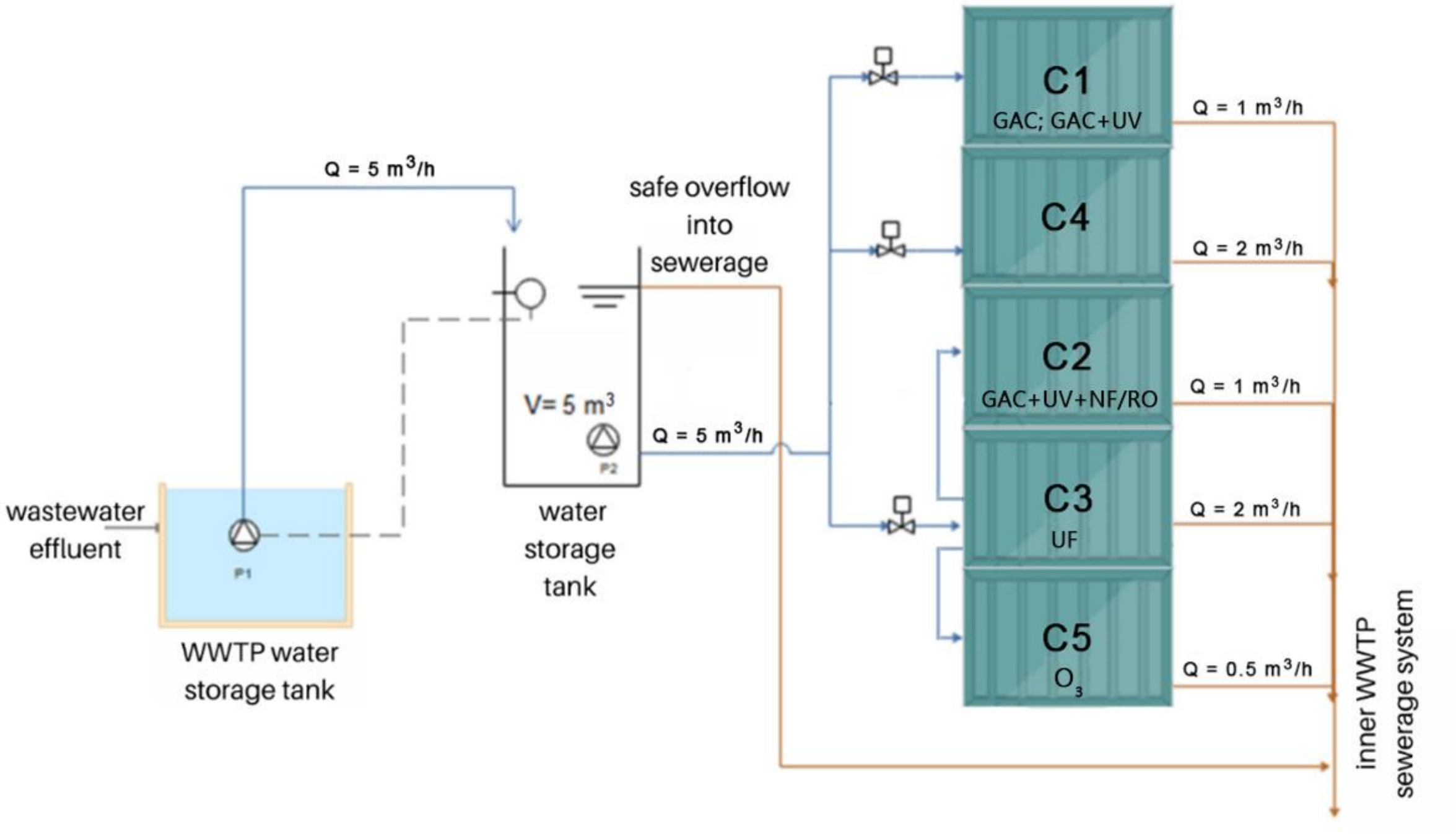

Figure S3. Technical drawing of the setup of technologies in containers 1, 2, 3, and 5. WWTPeff. samples were taken at point K1V1 or K3V1, GAC samples were taken at point K1V3, and GAC+UV samples were taken at point K1V4 in container 1. UF samples were taken at point K3V2 in container 3, and UF-treated water was directed to container 2, where UF+GAC+UV+RO samples were taken at point K2V3, and UF+GAC+UV+NF samples at point K2V6. UF-treated water was also directed to container 5, where UF+O_3 samples were taken at point K5K3V2.

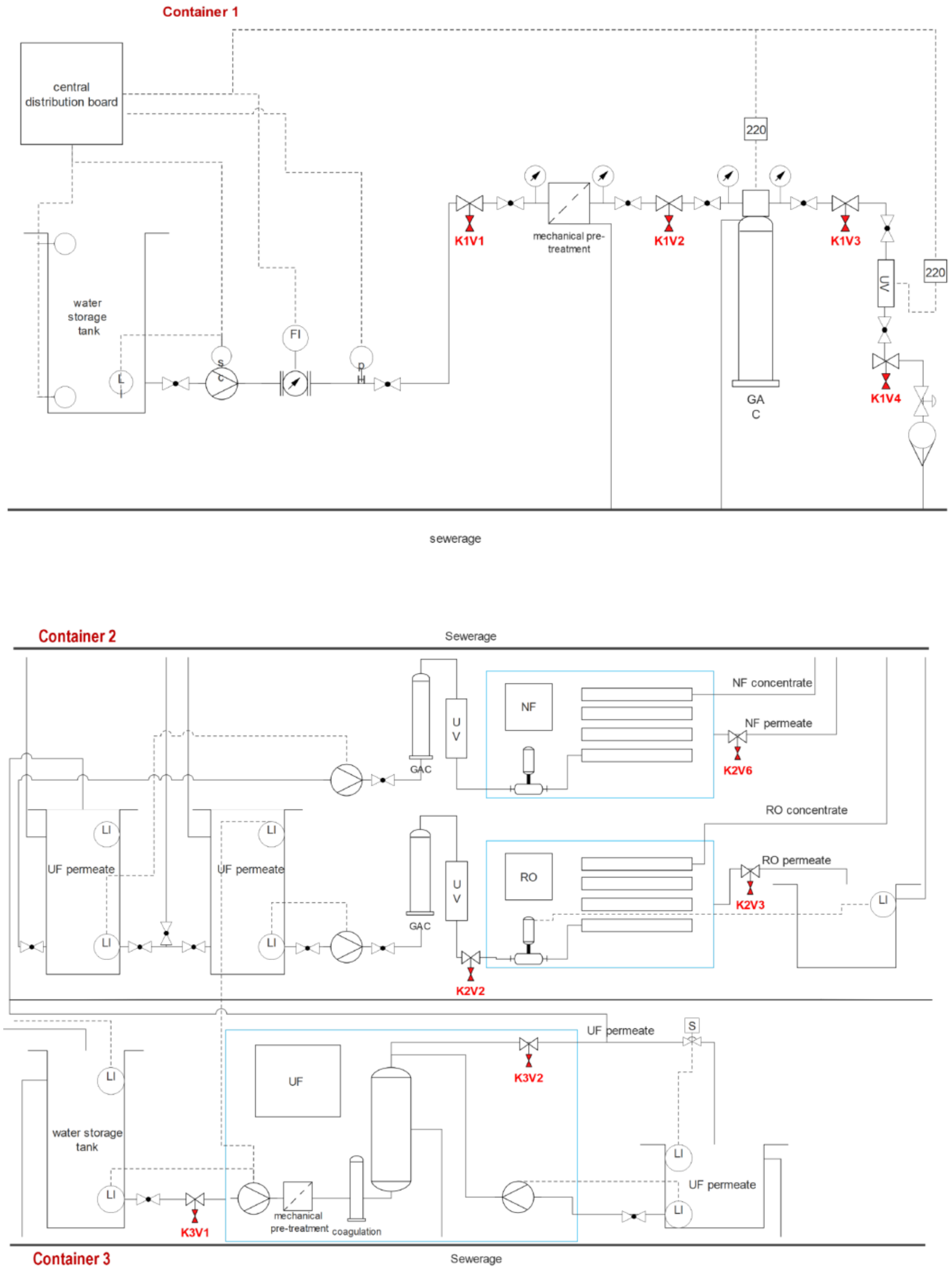

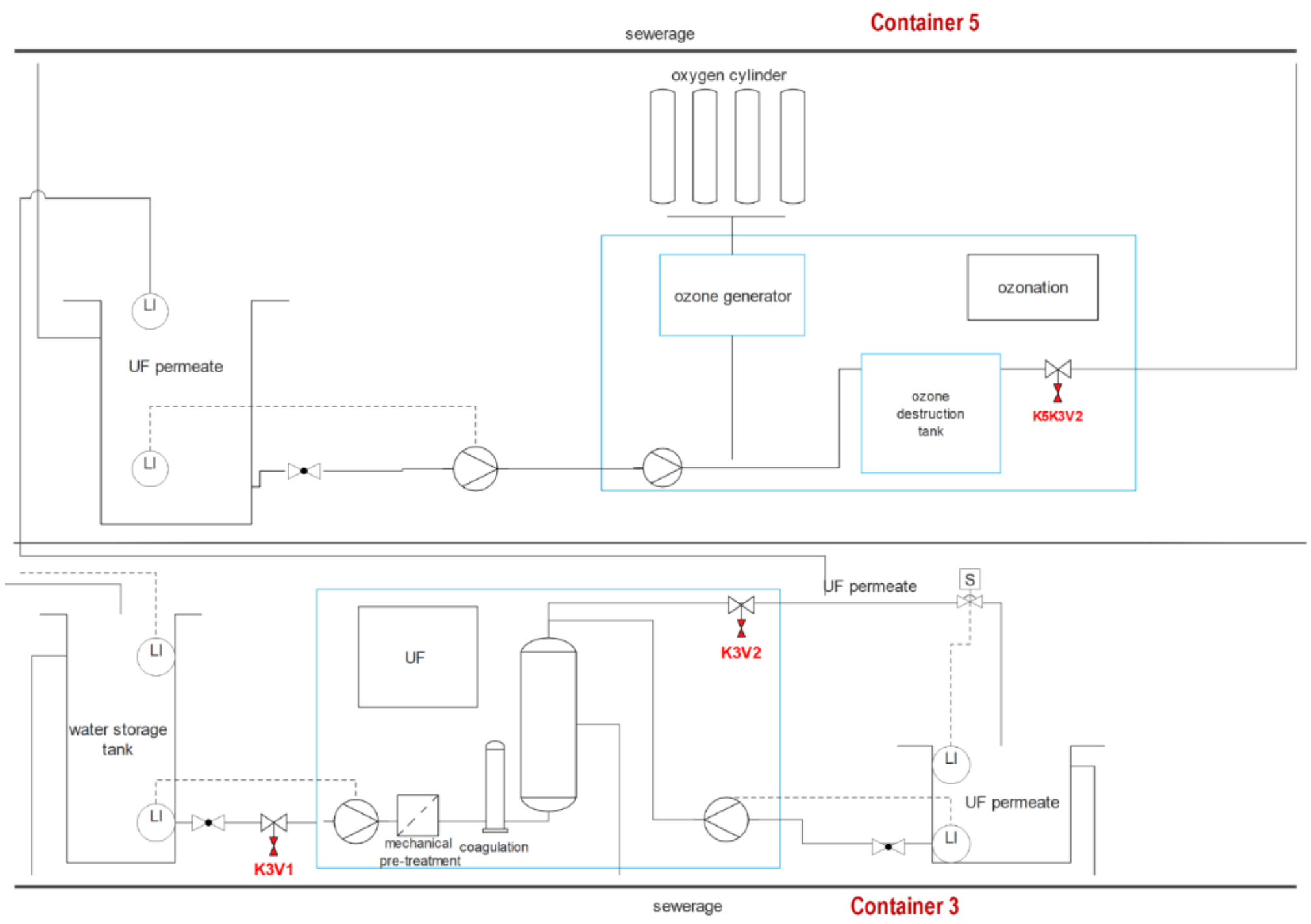
sewerage
Container 5
oxygen cylinder
ozone generator
ozonation
LI
UF permeate
LI
ozone destruction tank
K5K3V2
UF permeate
S
LI
UF
K3V2
water storage tank
LI
LI
UF permeate
LI
mechanical pre-treatment
coagulation
K3V1
sewerage
Container 3

Figure S4. Temperature of the WWTPeff. (i.e., inflow to the pilot QTT) during the studied period 16 March, 2021 – 29 March, 2022.

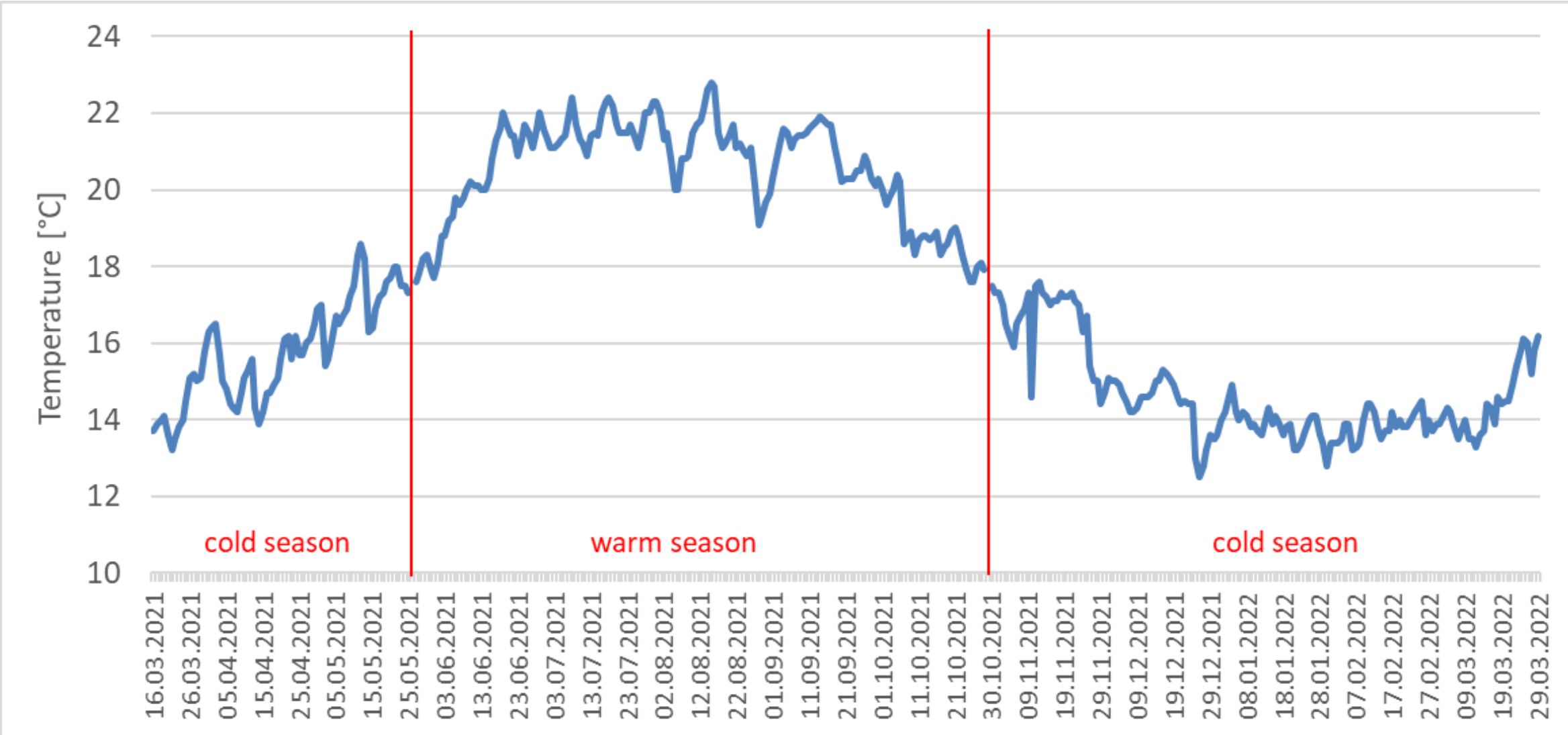

Figure S1. Occurrence patterns of pharmaceuticals in WWTP effluent samples over the year. Pharmaceuticals occurring in the highest concentrations show graph A with axis y ranging from 0–3,000 ng/L; medium concentrations show graph B with axis y ranging from 0–1,200 ng/L; lowest concentrations show graph C with axis y ranging from 0–250 ng/L.

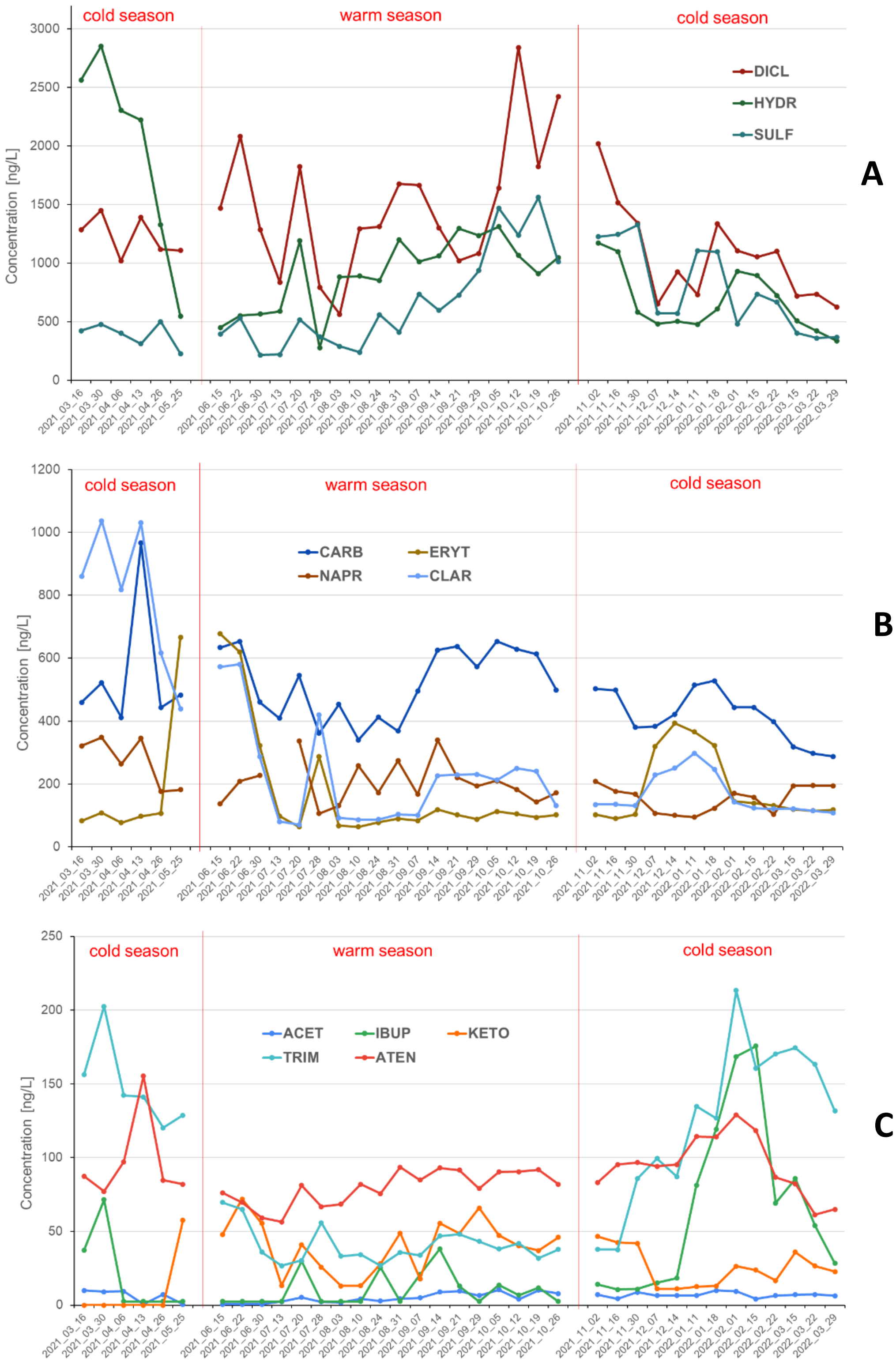

Figure S6. Correlation of concentration of pharmaceuticals in WWTP effluent with the temperature of WWTP effluent during the year. ΣPhACs (the first graph), and individual PhACs in other graphs.

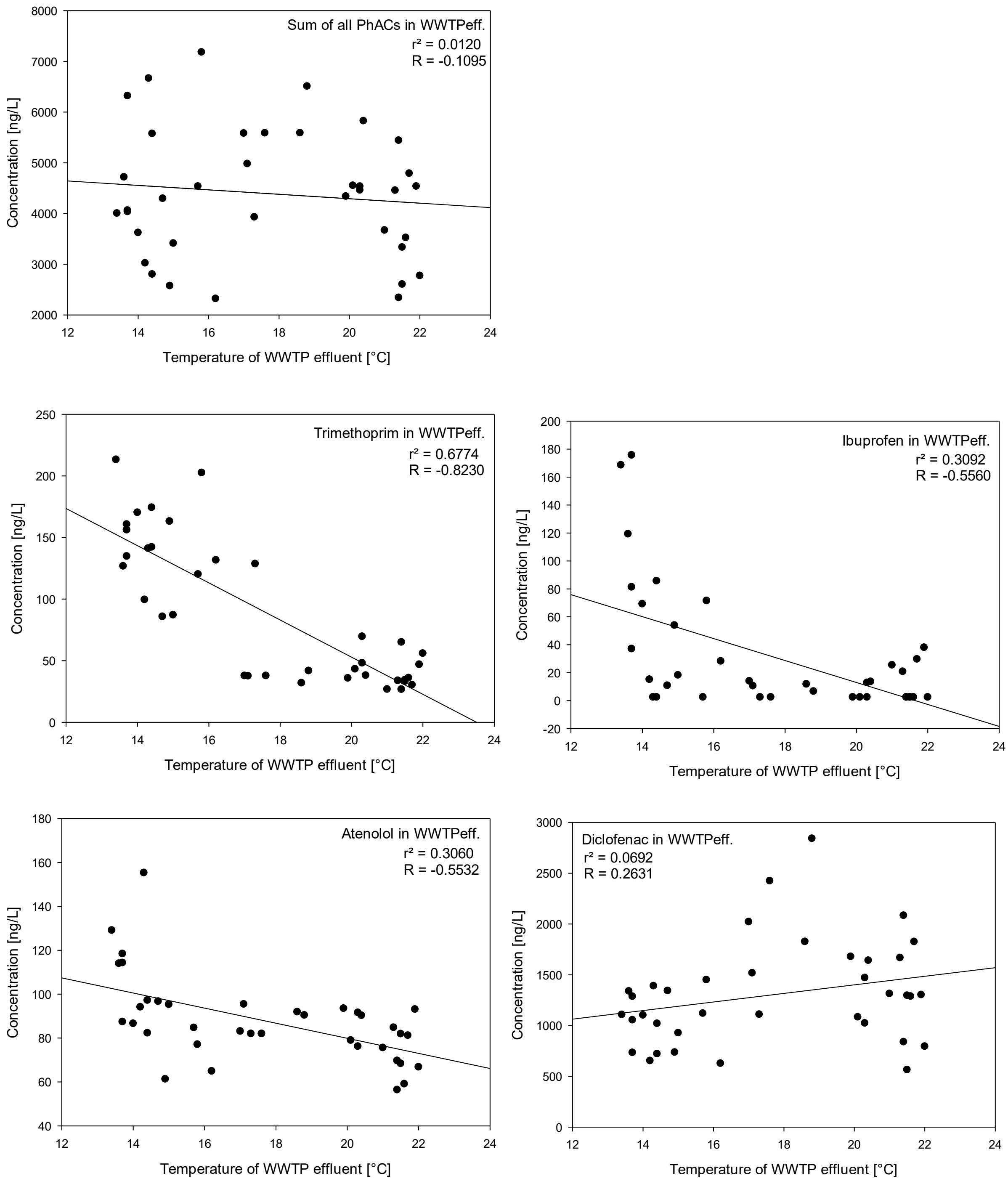

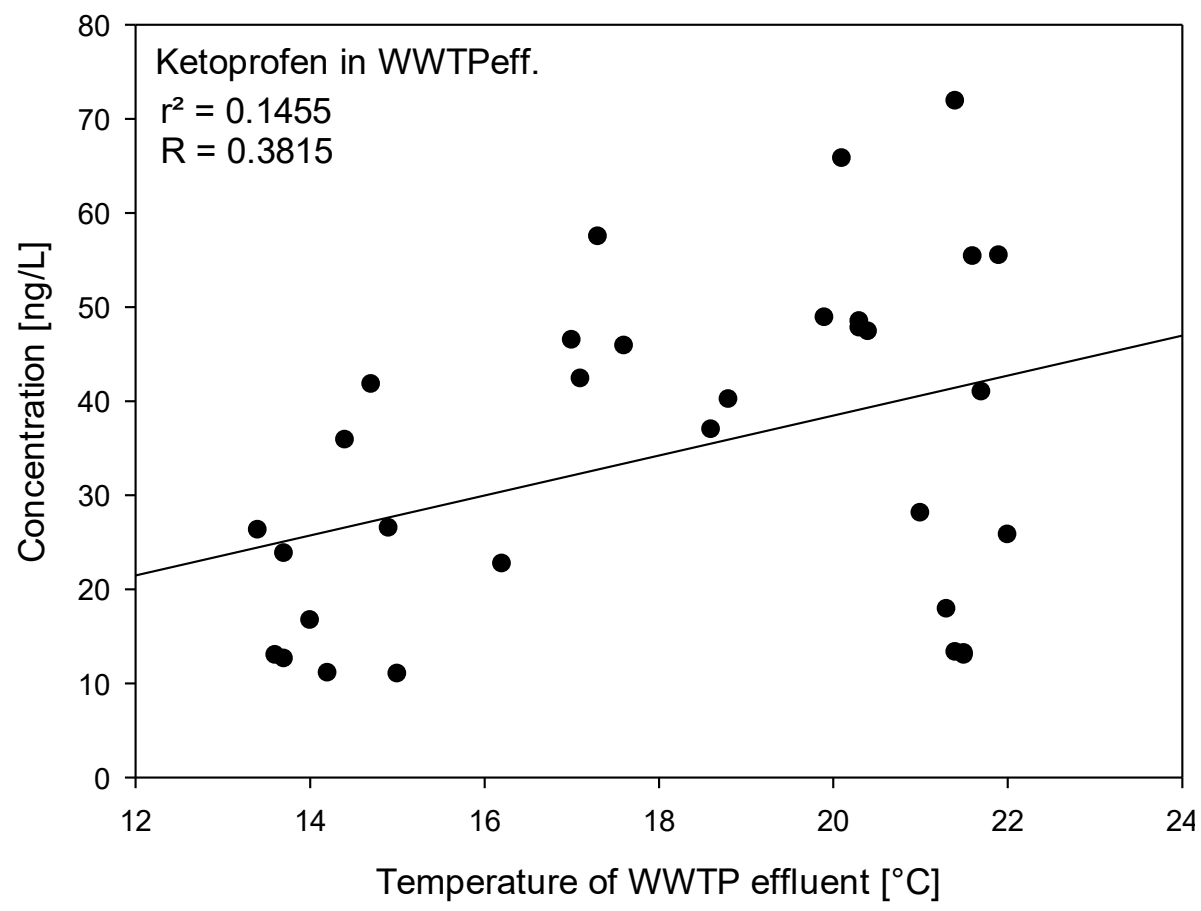
Ketoprofen in WWTPeff.
r² = 0.1455
R = 0.3815
Concentration [ng/L]
Temperature of WWTP effluent [°C]

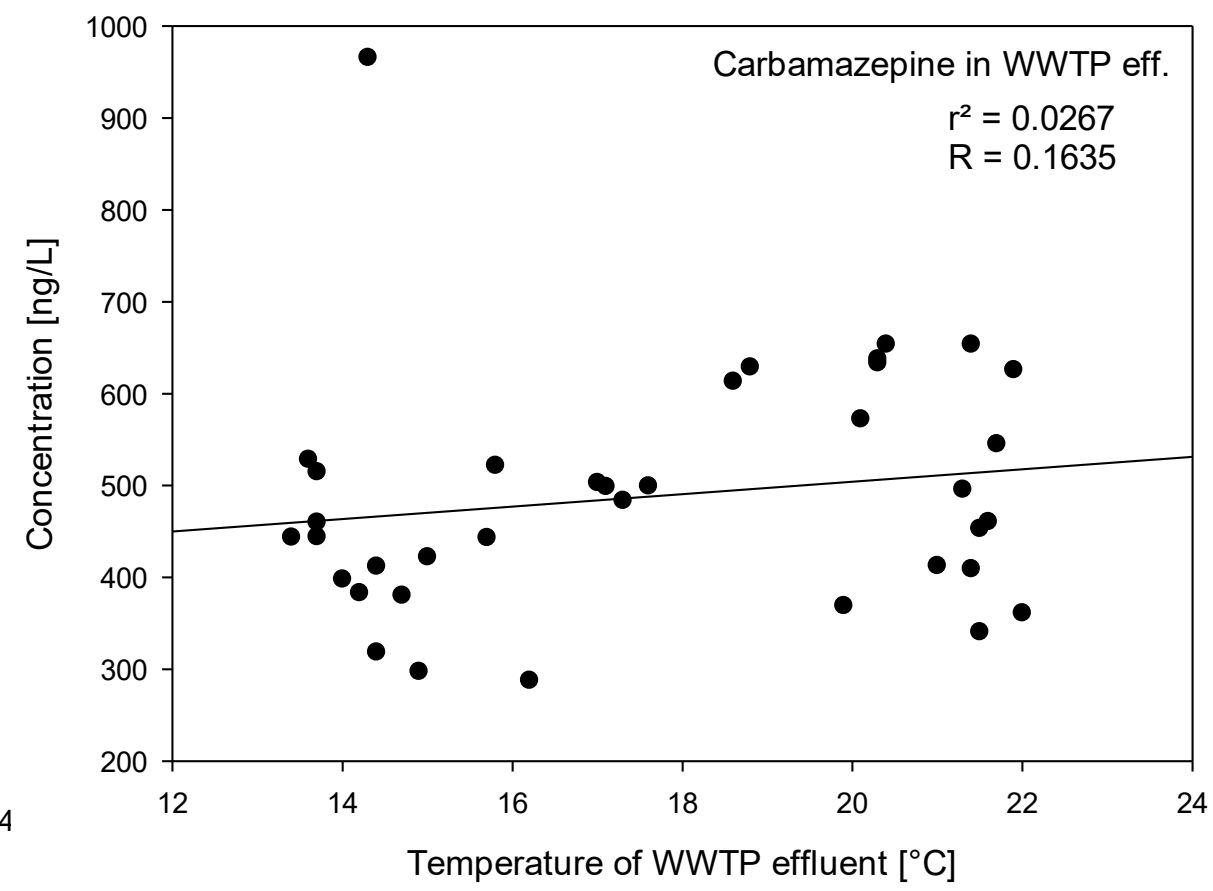
Carbamazepine in WWTP eff.
r² = 0.0267
R = 0.1635
Concentration [ng/L]
Temperature of WWTP effluent [°C]

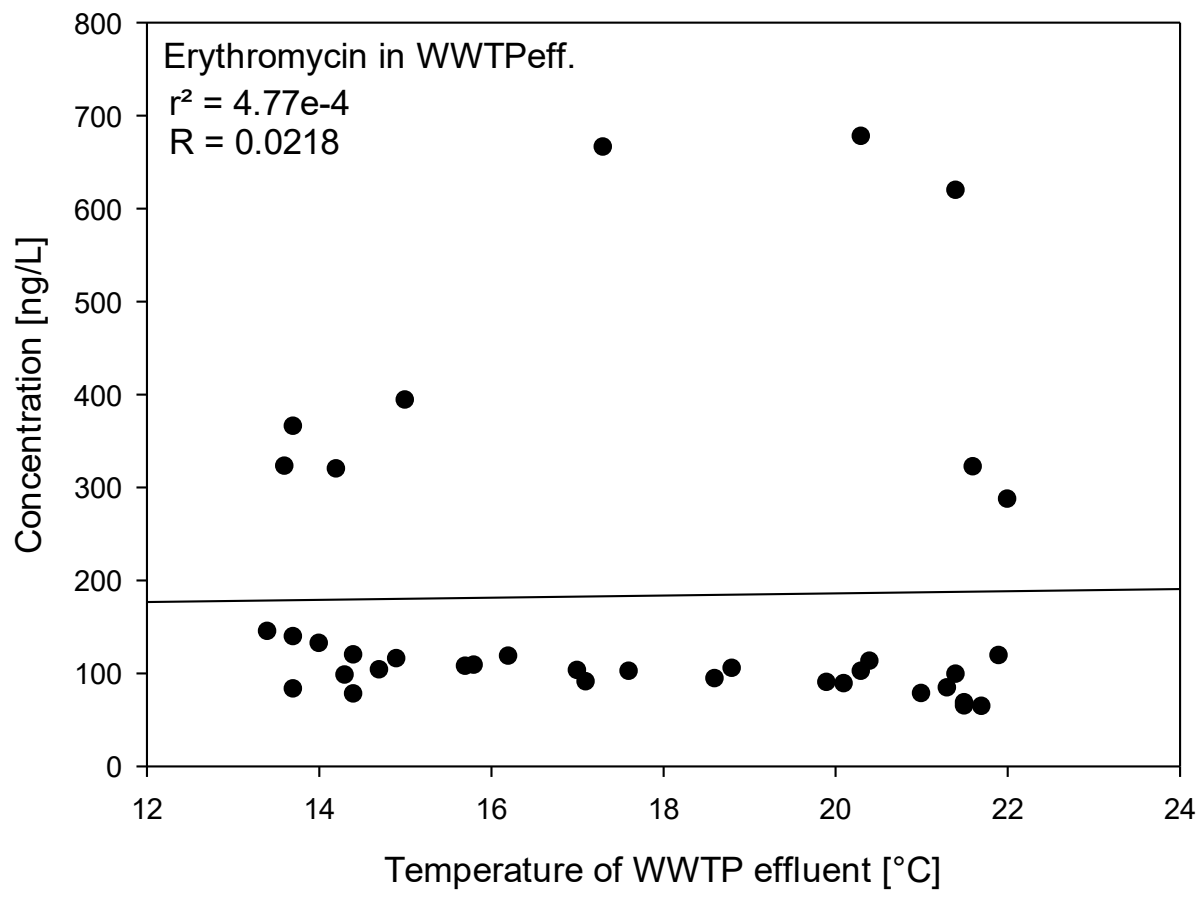
Erythromycin in WWTPeff.
r² = 4.77e-4
R = 0.0218
Concentration [ng/L]
Temperature of WWTP effluent [°C]

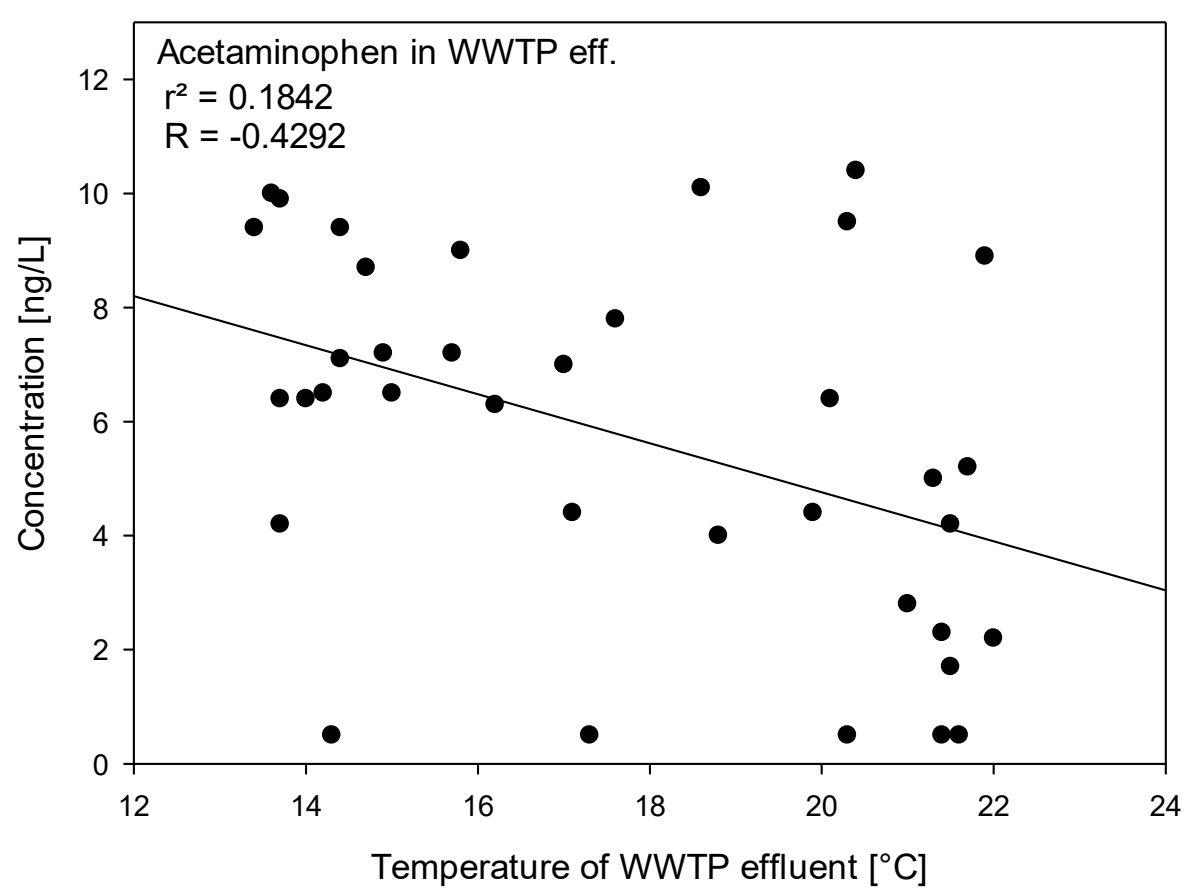
Acetaminophen in WWTP eff.
r² = 0.1842
R = -0.4292
Concentration [ng/L]
Temperature of WWTP effluent [°C]

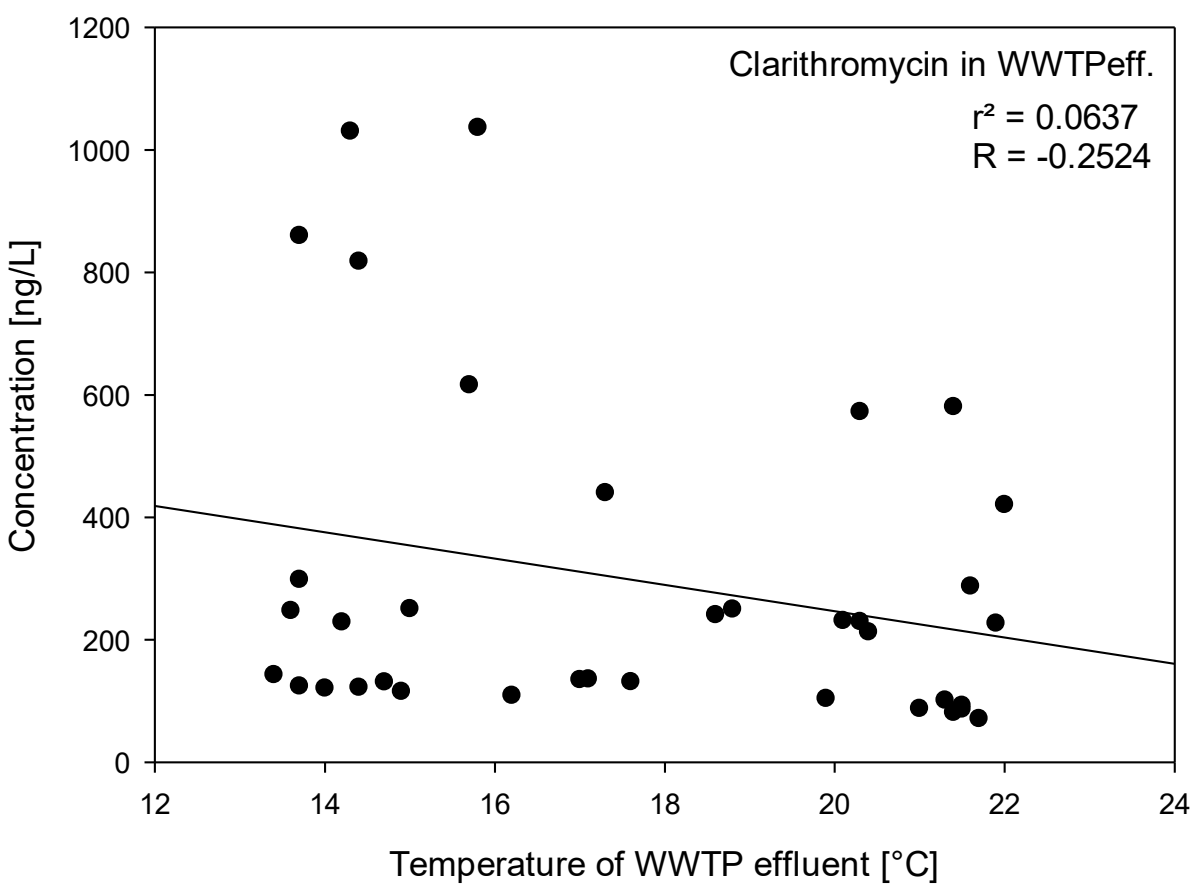
Clarithromycin in WWTPeff.
r² = 0.0637
R = -0.2524
Concentration [ng/L]
Temperature of WWTP effluent [°C]

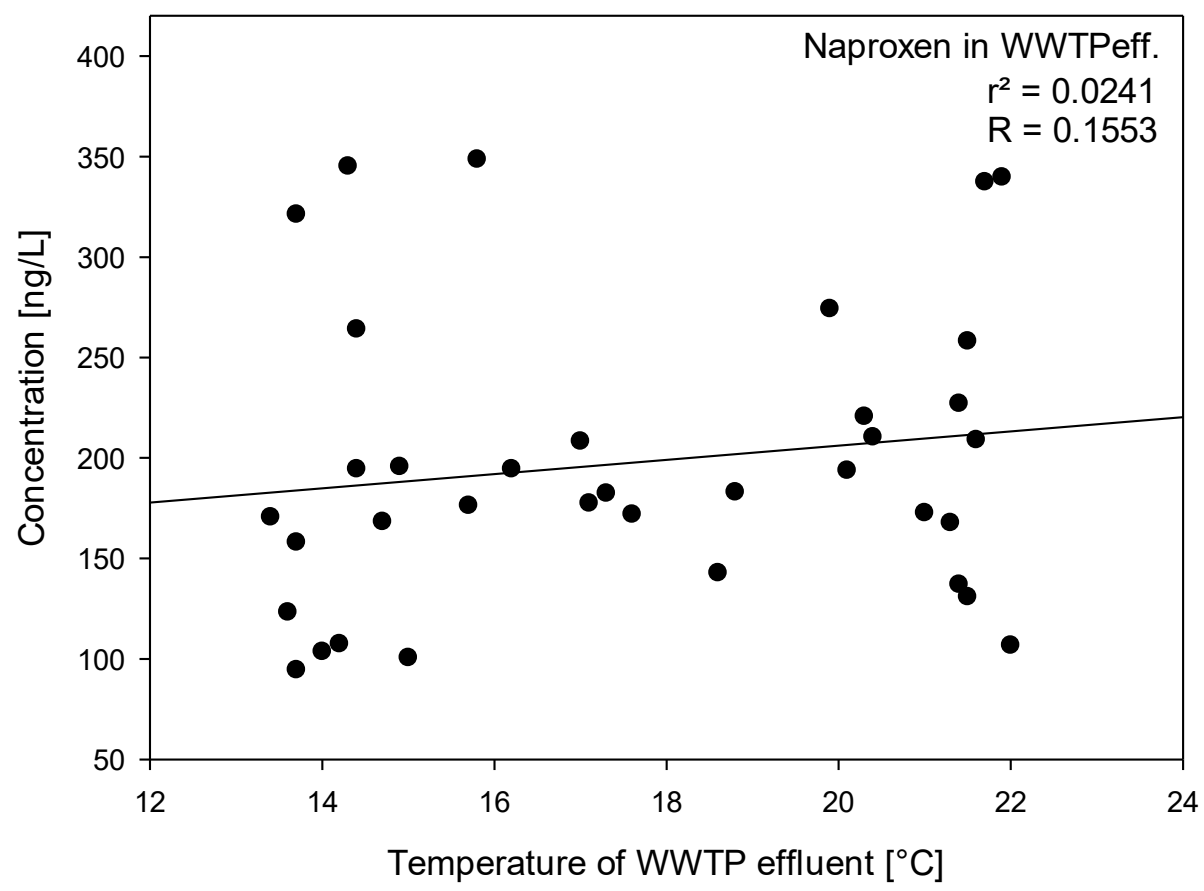
Naproxen in WWTPeff.
r² = 0.0241
R = 0.1553
Concentration [ng/L]
Temperature of WWTP effluent [°C]

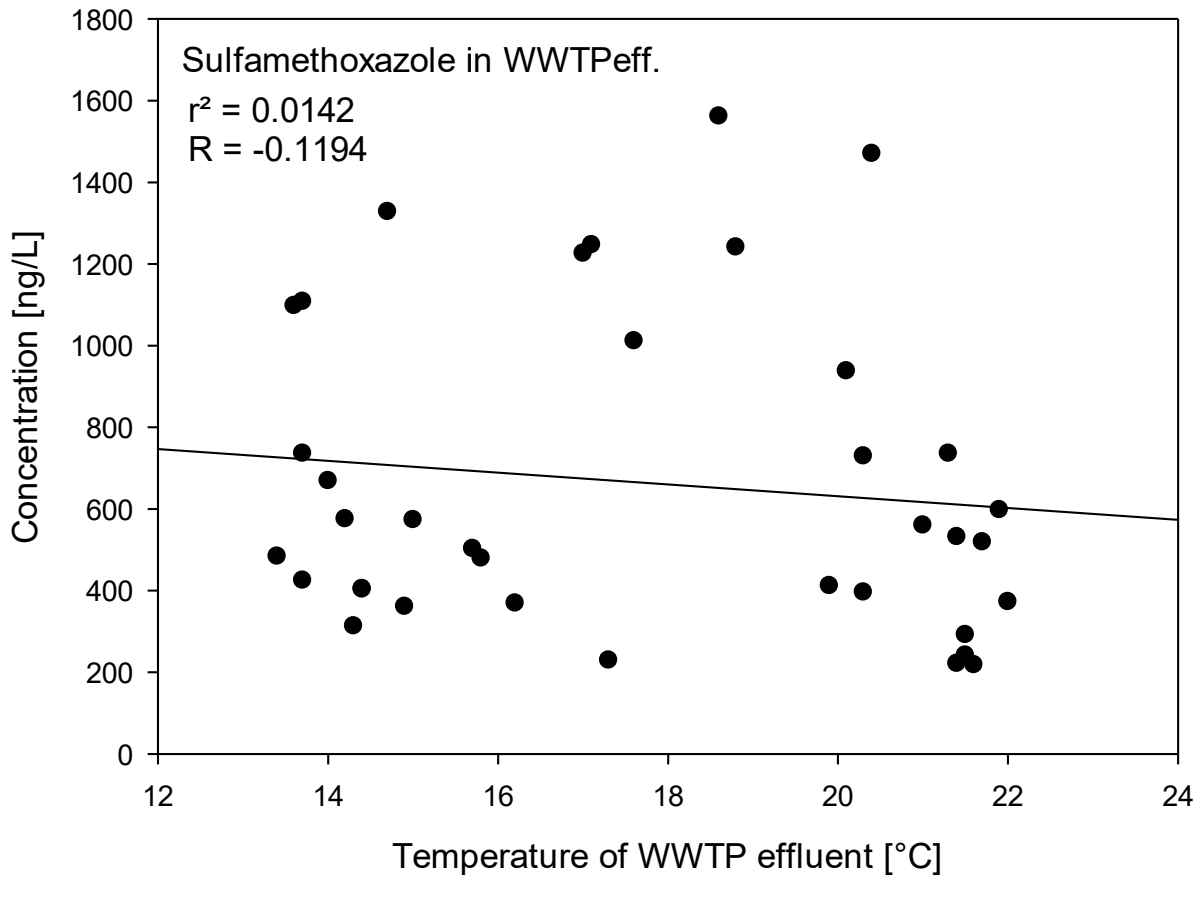
Sulfamethoxazole in WWTPeff.
r² = 0.0142
R = -0.1194
Concentration [ng/L]
Temperature of WWTP effluent [°C]

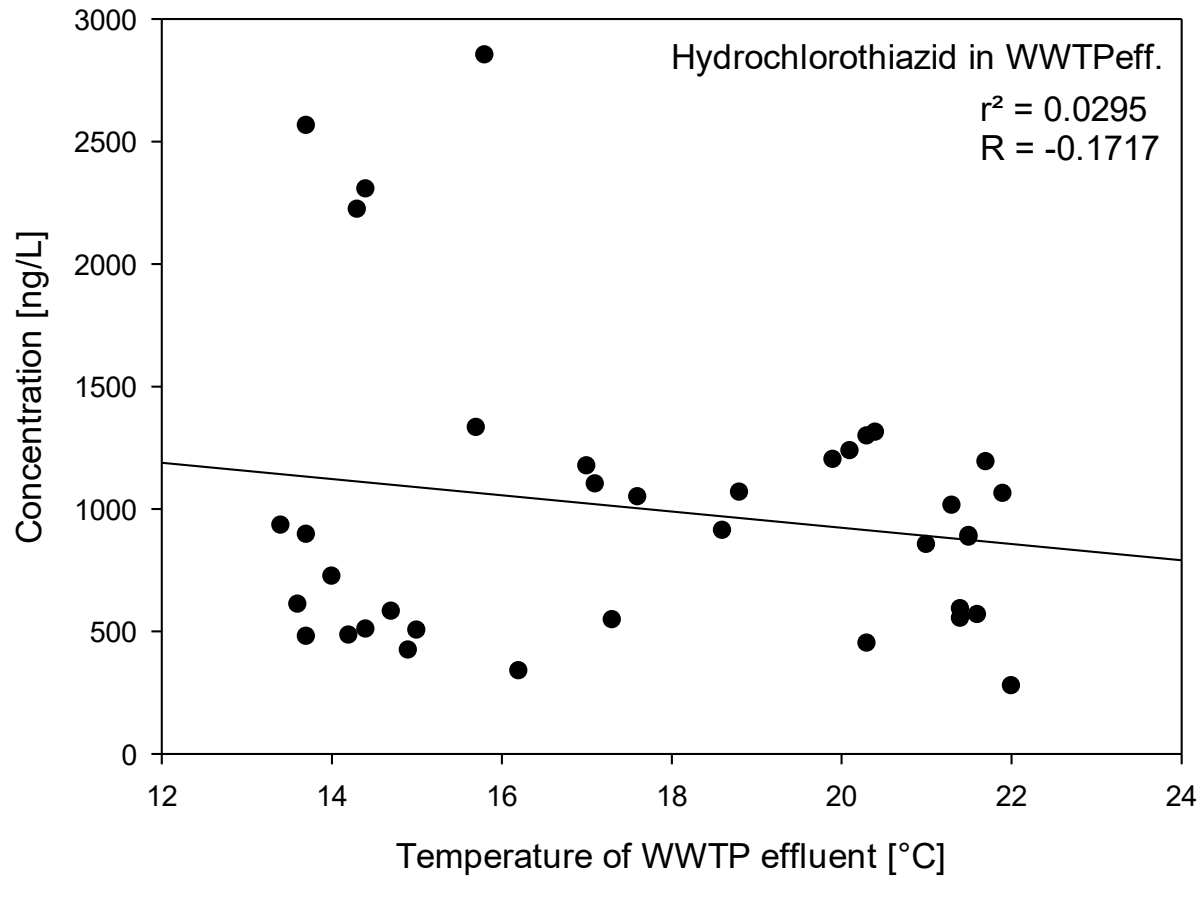
Hydrochlorothiazid in WWTPeff.
r² = 0.0295
R = -0.1717
Concentration [ng/L]
Temperature of WWTP effluent [°C]

Figure S7. Occurence of PhACs in WWTPeff. in a cold or warm period of the year. ΣPhACs (the first graph), and individual PhACs in other graphs (ordered according to the increasing P value). The boxes define the 25th and 75th percentiles, with a line at the median; the error bars are the 10th and 90th percentiles; and outlying points are shown.

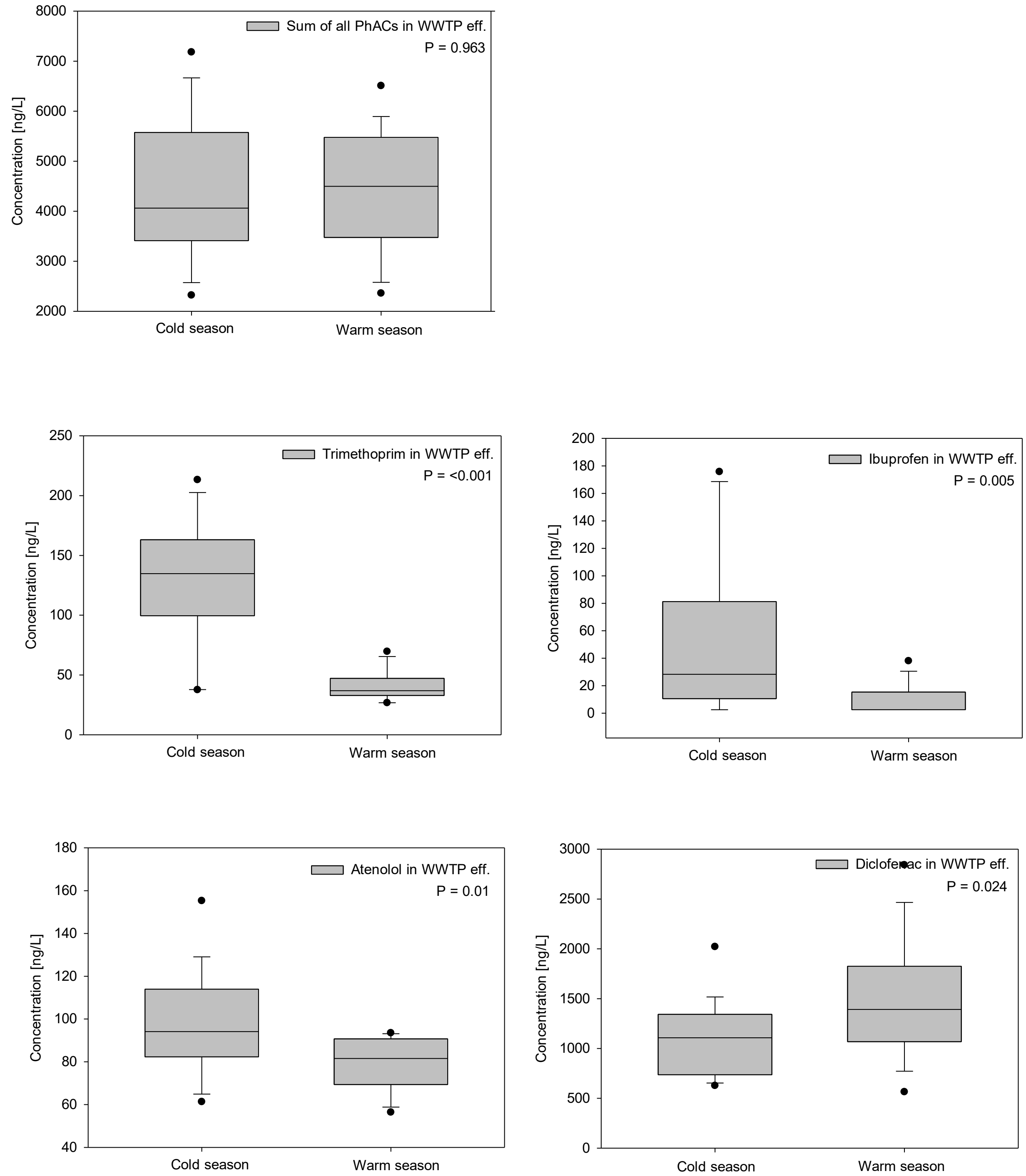

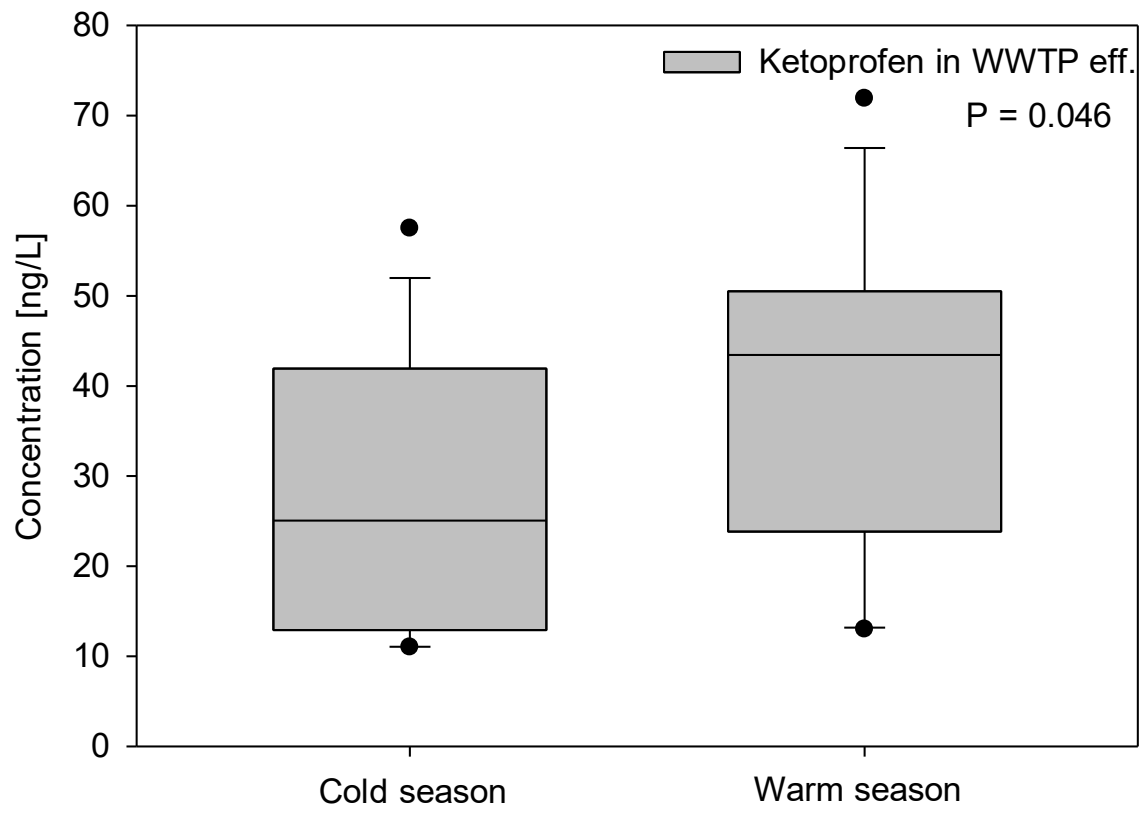
Ketoprofen in WWTP eff.
P = 0.046
Concentration [ng/L]
0
10
20
30
40
50
60
70
80
Cold season
Warm season

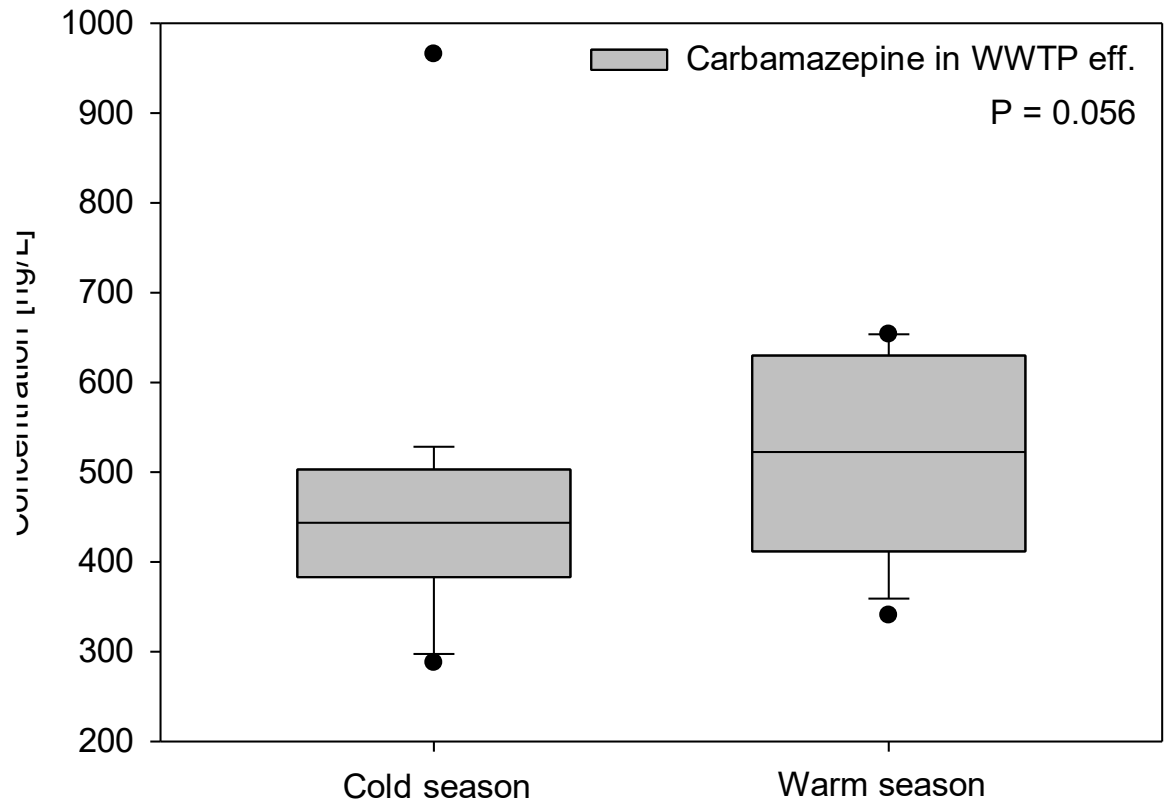
Carbamazepine in WWTP eff.
P = 0.056
200
300
400
500
600
700
800
900
1000
Cold season
Warm season

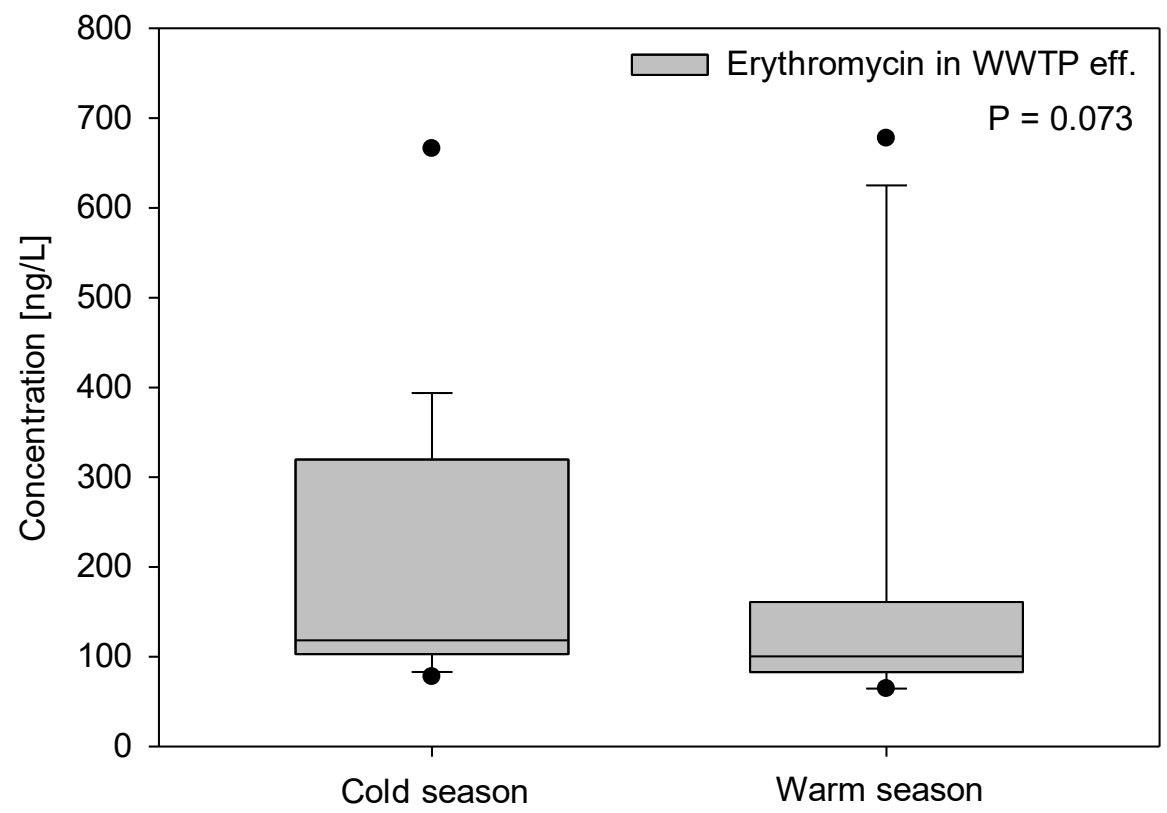
Erythromycin in WWTP eff.
P = 0.073
Concentration [ng/L]
0
100
200
300
400
500
600
700
800
Cold season
Warm season

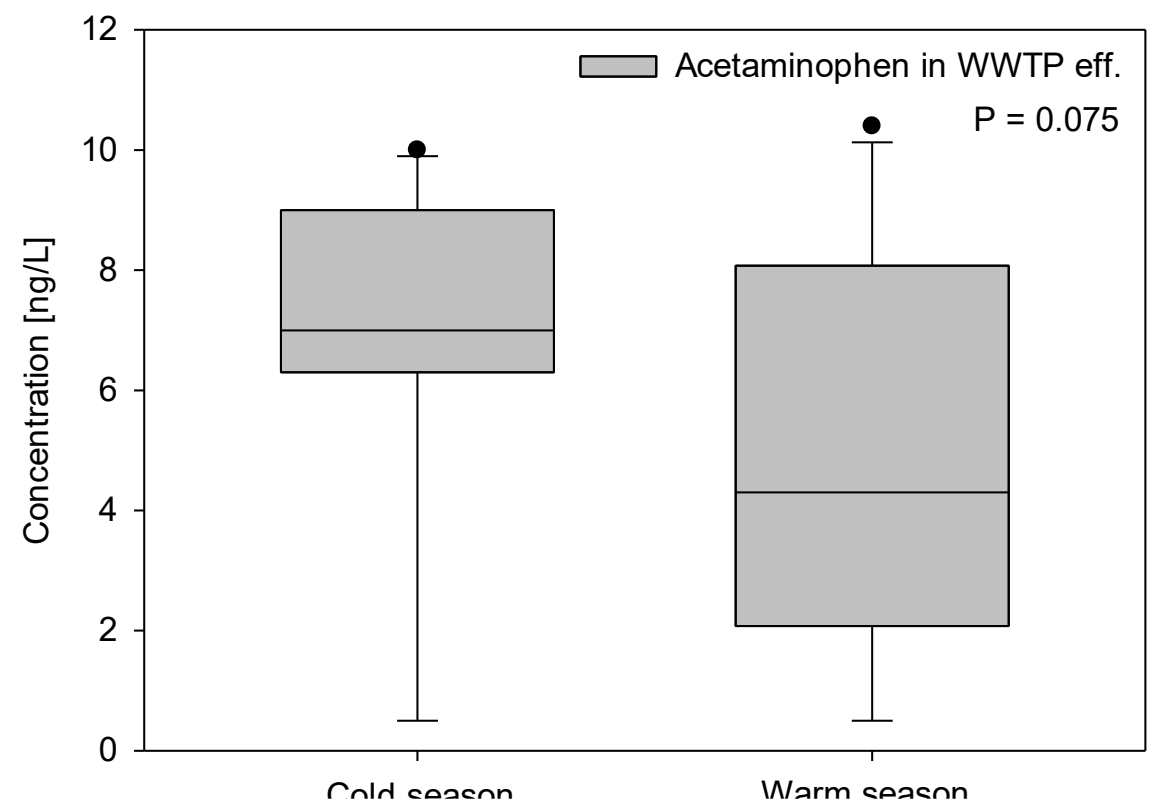
Acetaminophen in WWTP eff.
P = 0.075
Concentration [ng/L]
0
2
4
6
8
10
12

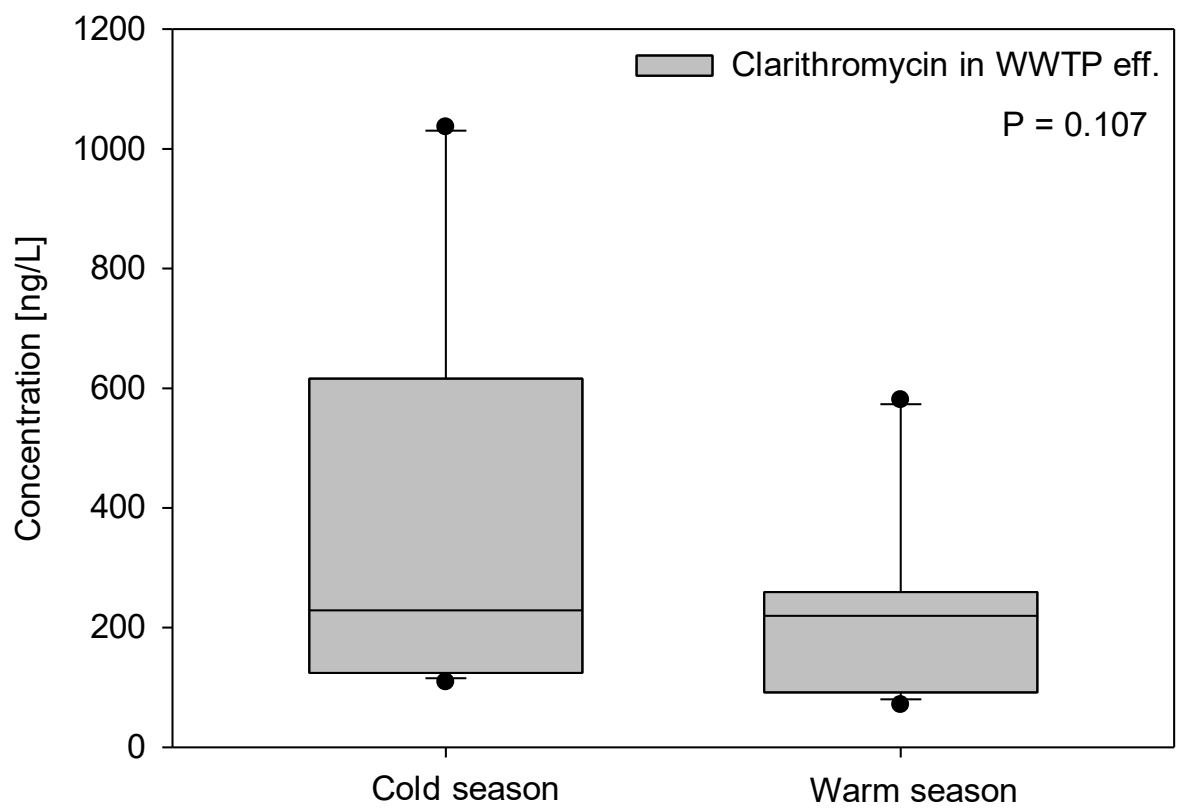
Clarithromycin in WWTP eff.
P = 0.107
Concentration [ng/L]
0
200
400
600
800
1000
1200
Cold season
Warm season

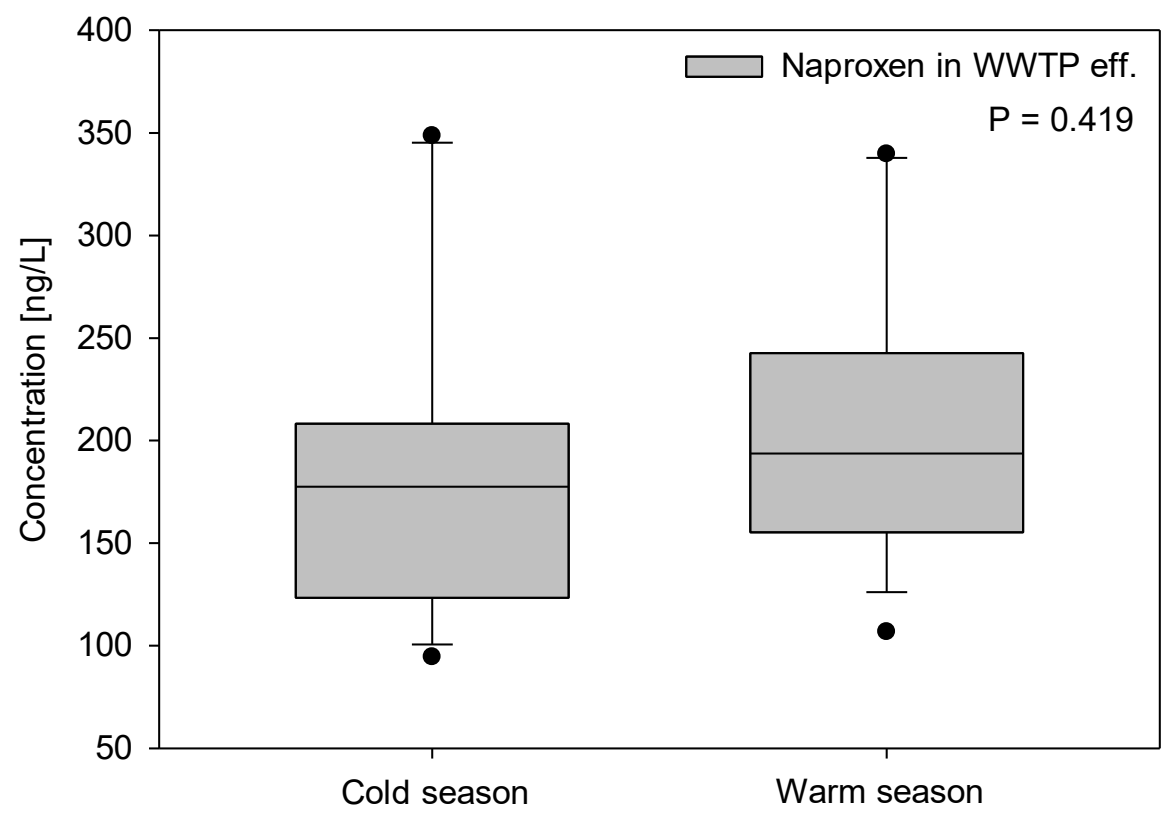
Naproxen in WWTP eff.
P = 0.419
Concentration [ng/L]
50
100
150
200
250
300
350
400
Cold season
Warm season

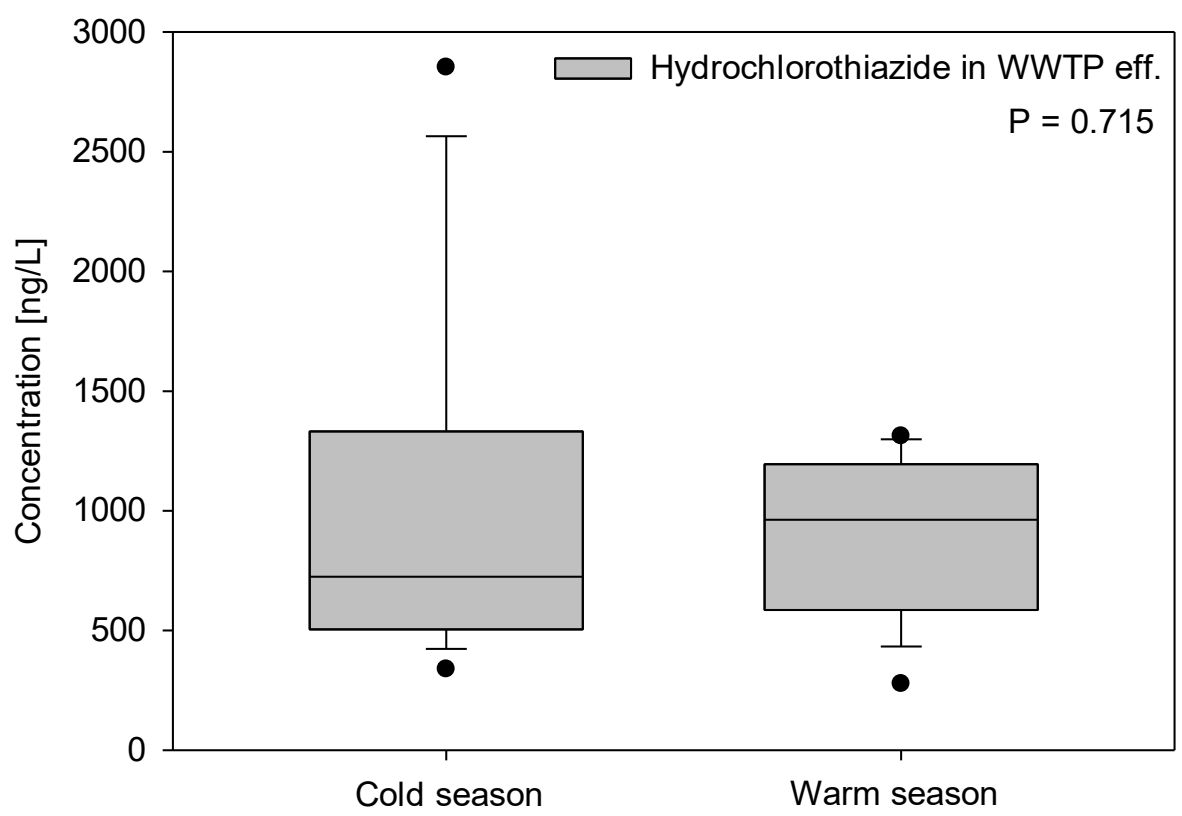
Hydrochlorothiazide in WWTP eff.
P = 0.715
Concentration [ng/L]
0
500
1000
1500
2000
2500
3000
Cold season
Warm season

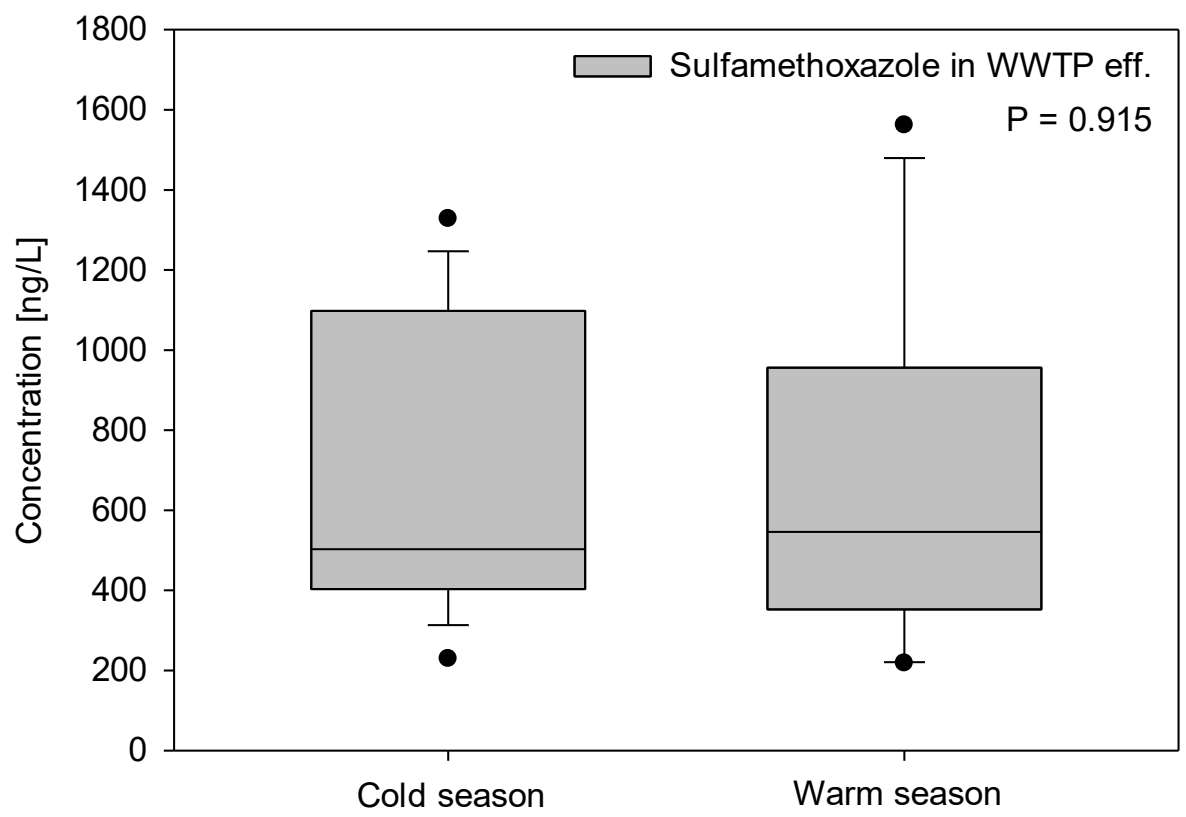
Sulfamethoxazole in WWTP eff.
P = 0.915
Concentration [ng/L]
0
200
400
600
800
1000
1200
1400
1600
1800
Cold season
Warm season

Figure S8. Flow rate of the WWTP effluent in cold and warm seasons of the studied period (16 March, 2021 – 29 March, 2022). Only the data for flow rates from the PhACs sampling days were used. The boxes define the 25th and 75th percentiles, with a line at the median; error bars are the 10th and 90th percentiles and outlying points.

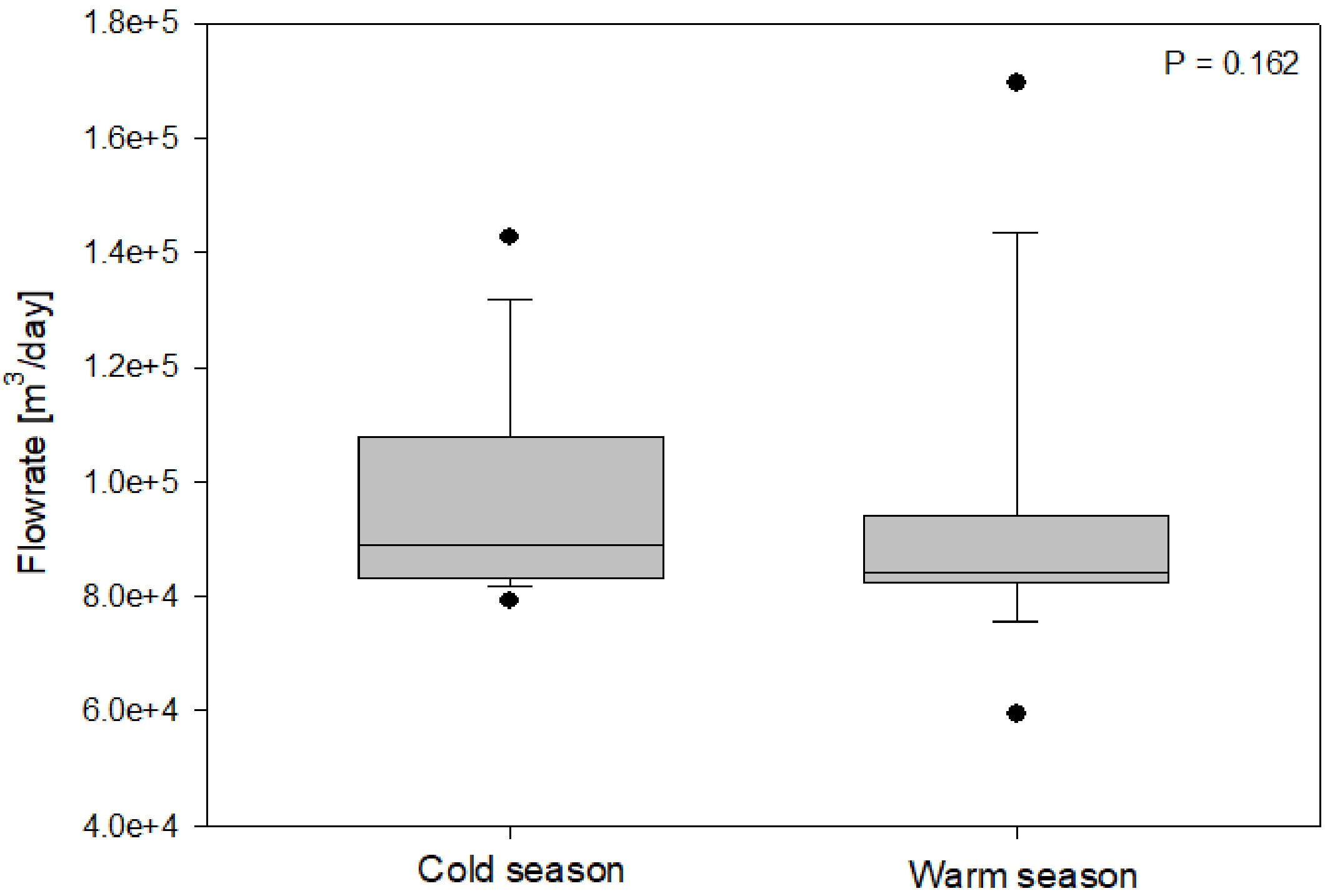

Figure S2. Correlation ($p = 0.27$) of concentration of ΣPhACs in WWTPeff. with flowrate of the WWTPeff. during the year 2021.

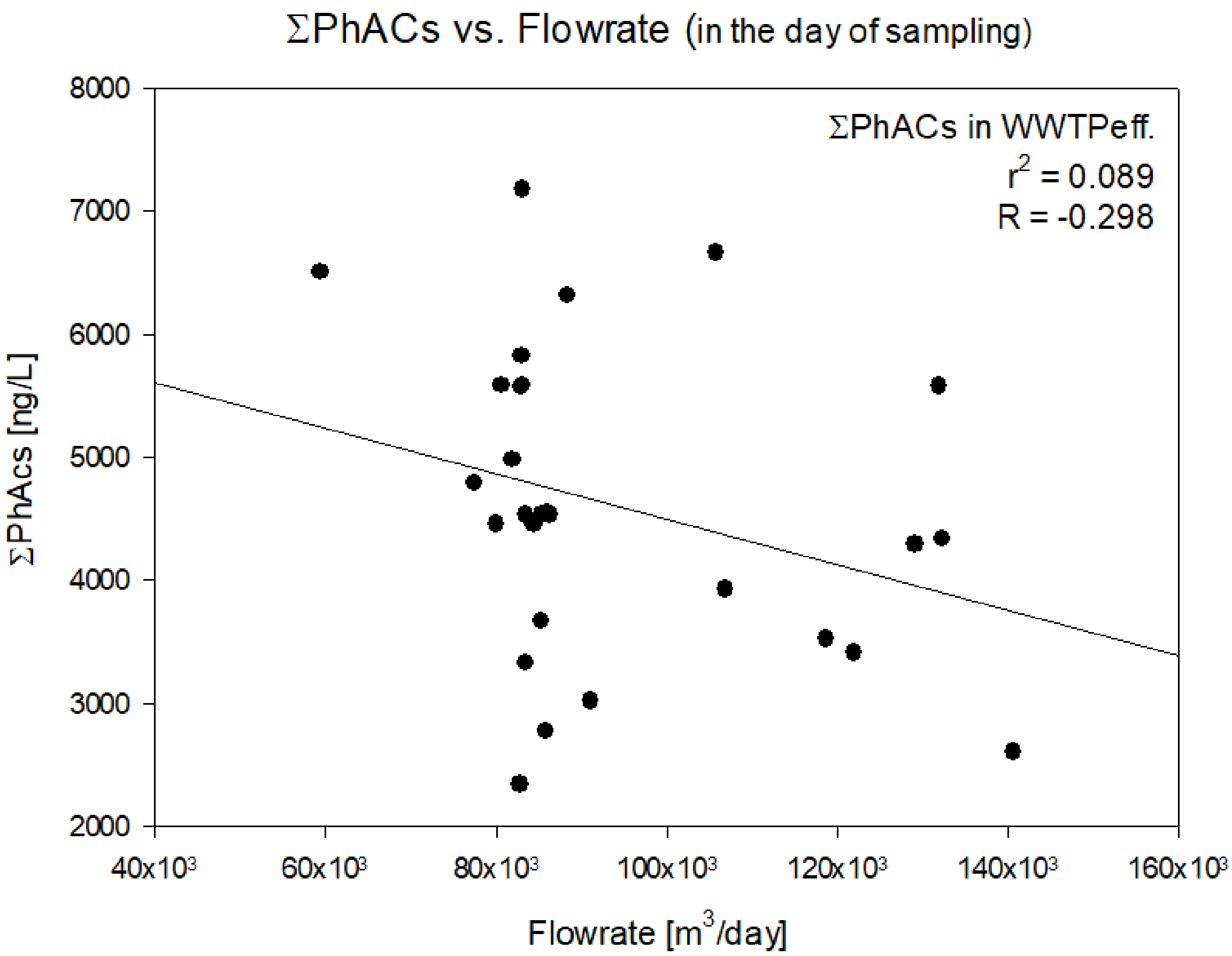

Figure S10. Environmental exposure distributions (EED) for all PhACs in the cold season (left column) and warm season (right column). Vertical dash lines represent the PNEC value, and vertical dash-dotted lines represent the PNEC-AR value (shown in the last two columns). The dotted lines represent the ½LOQ value (2.5 ng/L for ibuprofen and 0.5 ng/L for remaining PhACs). Samples with concentrations <LOQ are aggregated at a single point at ½LOQ without a regression curve, which was constructed from at least six samples (GAC cold technology was not compliant because of only four samples), and at least 80 % of samples above LOQ (UF+GAC+UV+RO and UF+O_3 technologies were often not compliant because many samples were <LOQ).

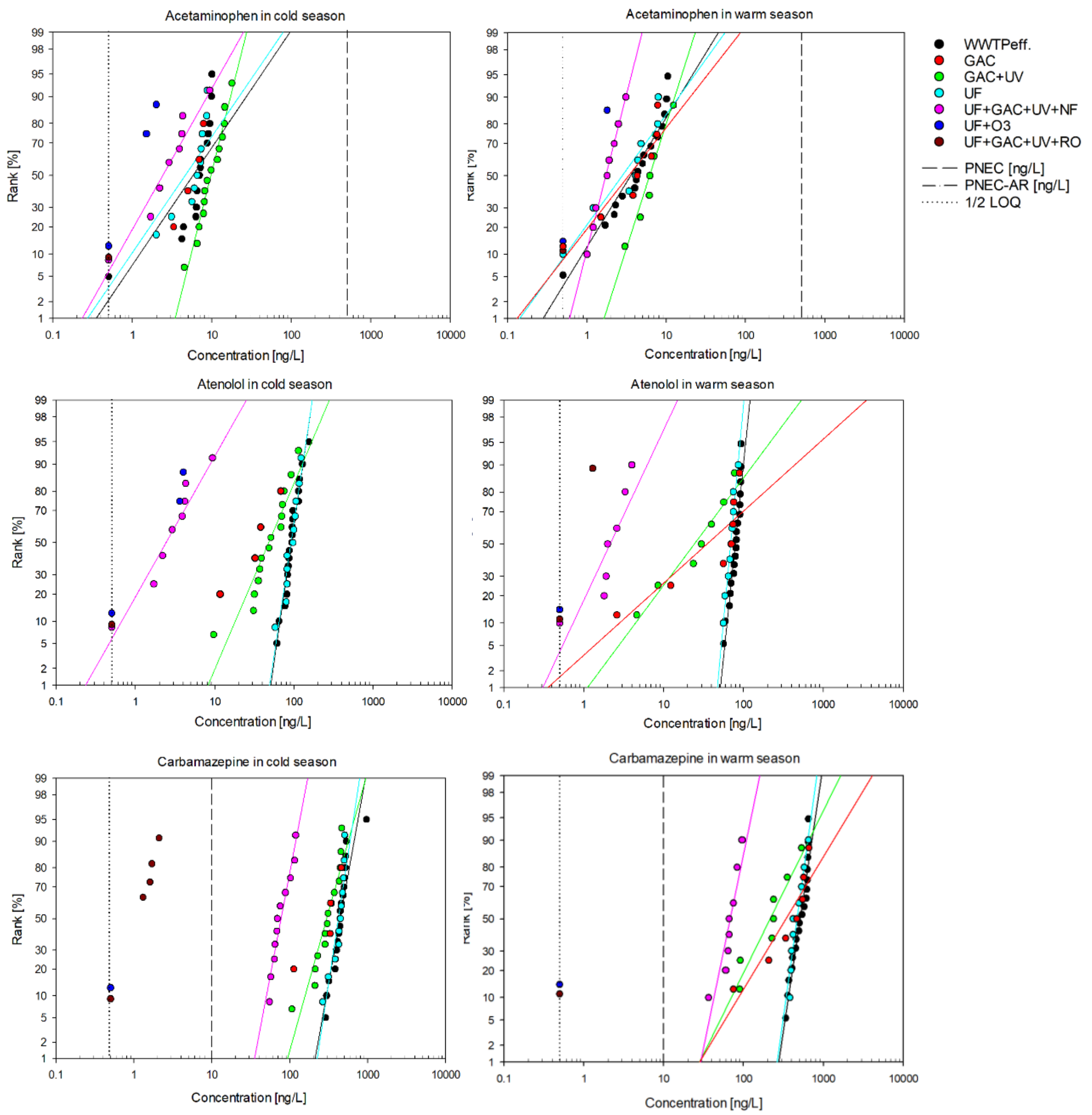

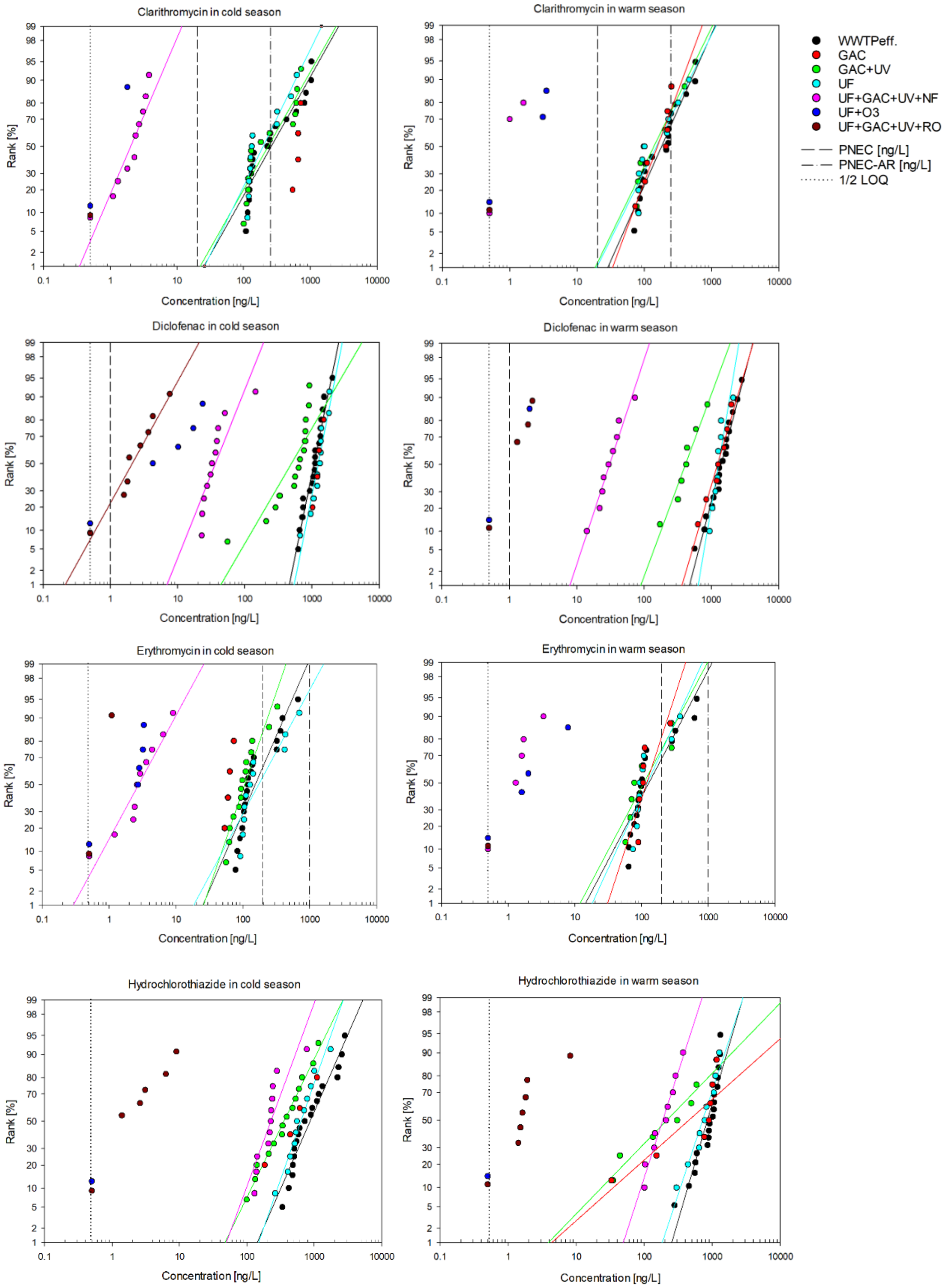
Clarithromycin in cold season
Clarithromycin in warm season
Diclofenac in cold season
Diclofenac in warm season
Erythromycin in cold season
Erythromycin in warm season
Hydrochlorothiazide in cold season
Hydrochlorothiazide in warm season
Rank [%]
Concentration [ng/L]
WWTPeff.
GAC
GAC+UV
UF
UF+GAC+UV+NF
UF+O3
UF+GAC+UV+RO
PNEC [ng/L]
PNEC-AR [ng/L]
1/2 LOQ

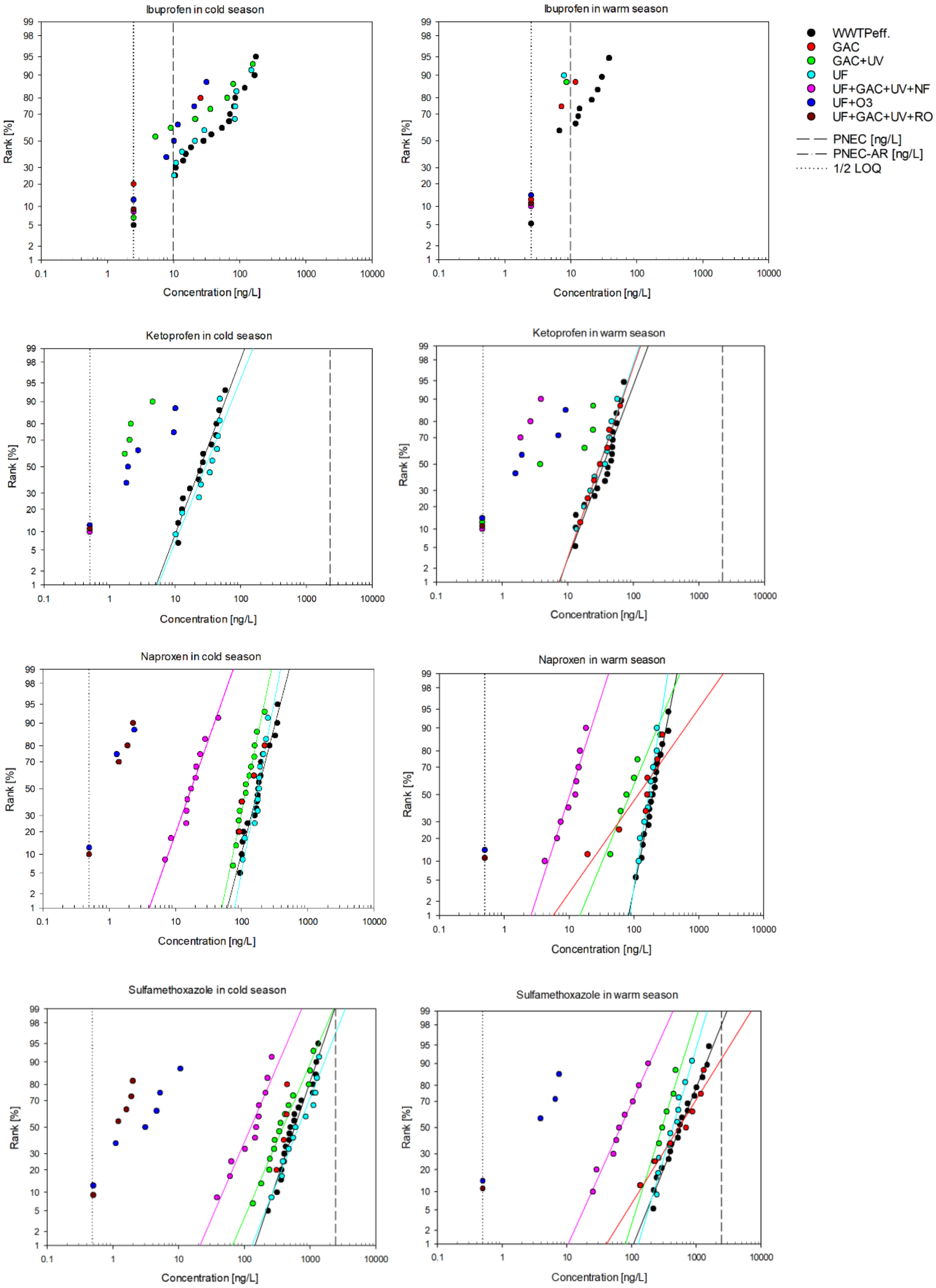
Ibuprofen in cold season
Ibuprofen in warm season
Ketoprofen in cold season
Ketoprofen in warm season
Naproxen in cold season
Naproxen in warm season
Sulfamethoxazole in cold season
Sulfamethoxazole in warm season
Rank [%]
Concentration [ng/L]
WWTPeff.
GAC
GAC+UV
UF
UF+GAC+UV+NF
UF+O3
UF+GAC+UV+RO
PNEC [ng/L]
PNEC-AR [ng/L]
1/2 LOQ

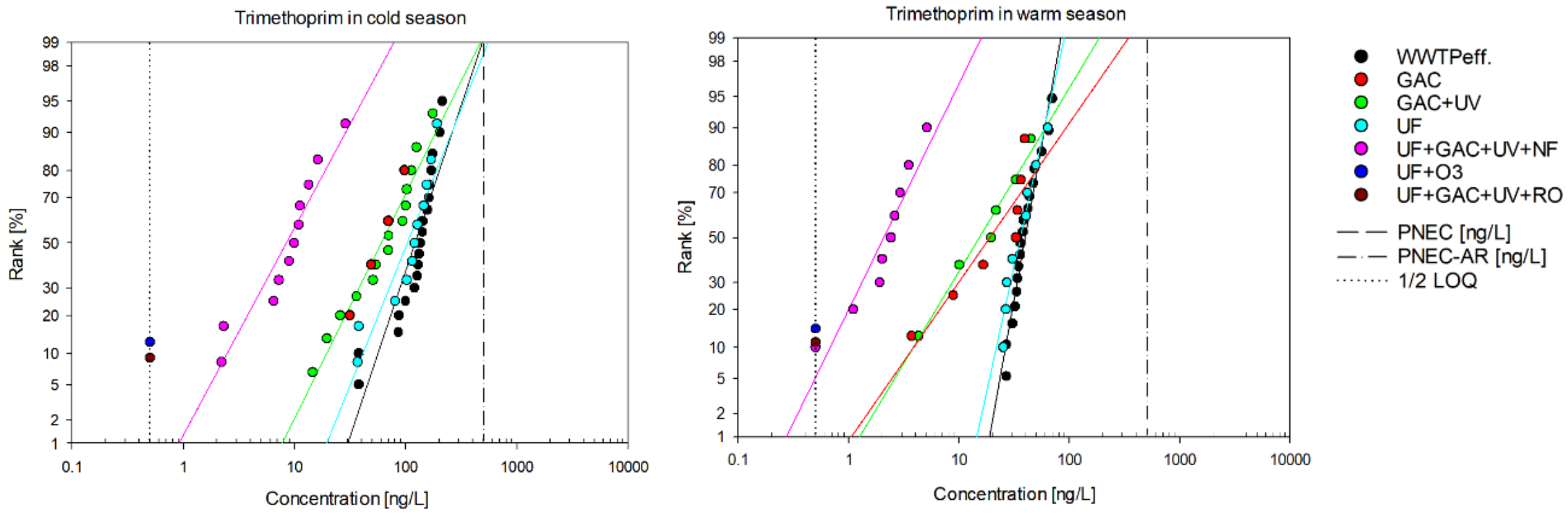
Trimethoprim in cold season
Rank [%]
Concentration [ng/L]
Trimethoprim in warm season
Rank [%]
Concentration [ng/L]
WWTPeff.
GAC
GAC+UV
UF
UF+GAC+UV+NF
UF+O3
UF+GAC+UV+RO
PNEC [ng/L]
PNEC-AR [ng/L]
1/2 LOQ

Figure S11. Percentage removal of ΣPhACs from wastewater by GAC or GAC+UV technologies. The new GAC was created before the project began and was exchanged twice: on 2021-06-26 and 2021-10-22.

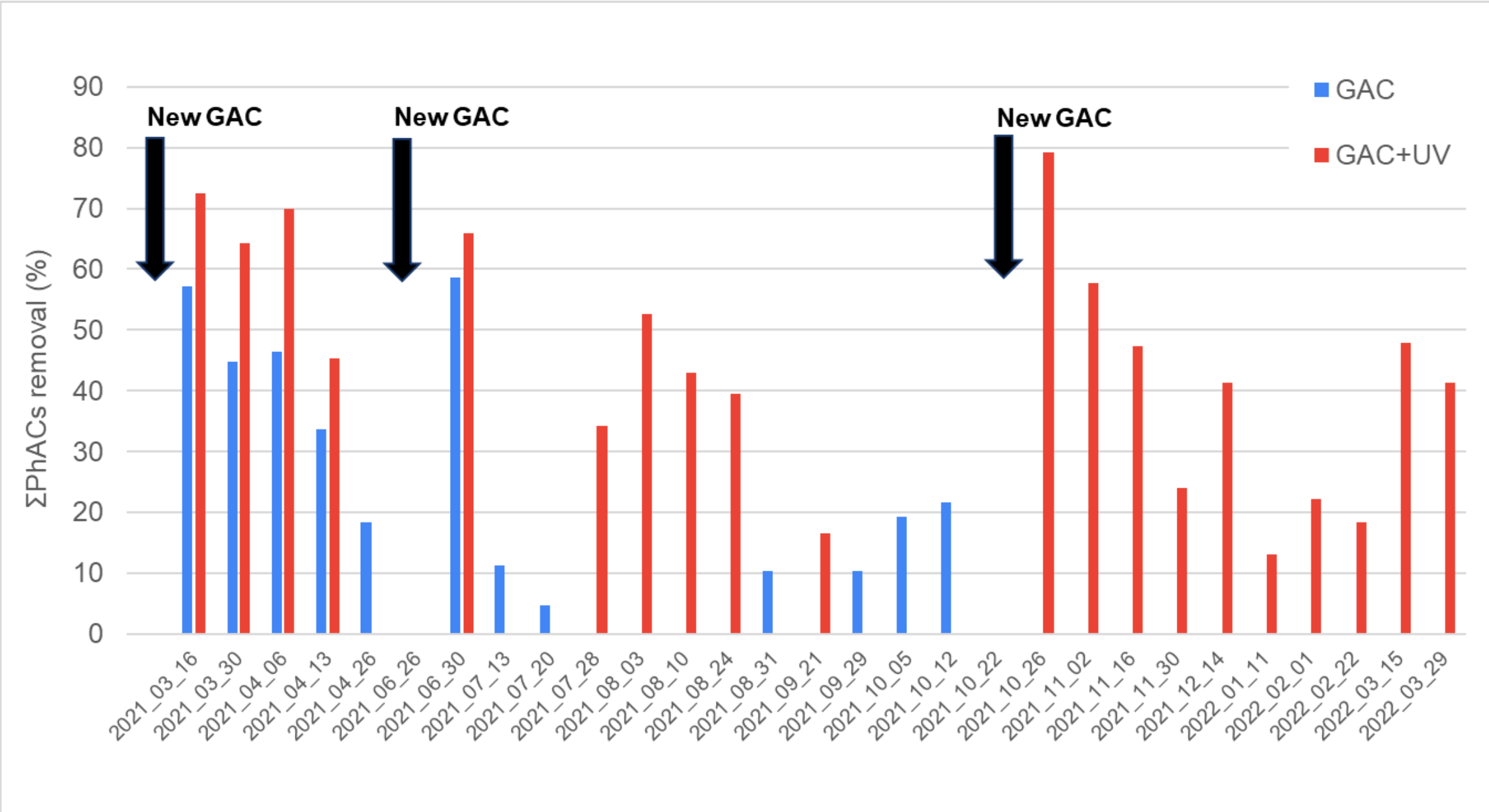

Table S1. Number of samples before quaternary treatment (WWTPeff.), and the number of samples collected after the individual QTT during the period from 16 March 2021 – 29 March, 2022 (exact sampling dates are stated in Tables S5–S11). WWTPeff. samples were taken at approximately two-to-six week intervals; corresponding QTT samples were taken less frequently because of the limited sample-processing capacity.

	WWTPeff.	UF	GAC	GAC+UV	UF+O_3	UF+GAC+UV+NF	UF+GAC+UV+RO
Cold season	19	11	4	14	6	11	11
Warm season	18	9	7	7	7	9	7

Table S2. Comparison of concentrations (ng/L) of PhACs in WWTPeff. sampled on 1 February, 2022, to PET bottle and a glass bottle.

	ACET	ATEN	CARB	CLAR	DICL	ERYT	HYDR	IBUP	KETO	NAPR	SULF	TRIM
PET	9.4	129.1	443.5	142.9	1107.2	144.8	932.9	168.6	26.3	170.6	483.8	213.3
Glass	9.9	131.8	431.3	125.8	1100.4	133.9	928.6	161.4	20.7	172.4	493.7	199.4

Table S3. MS/MS parameters of studied PhACs.

	Ionization mode	Precursor ion (*m/z*)	Capillary voltage (V)	Product ions (*m/z*)	Collision energy (eV)	LOQ (ng/L)
Acetaminophen	ESI+	152	30	110, 93	15, 25	1
Atenolol	ESI+	267	35	190, 145	20, 30	1
Carbamazepine	ESI+	237	30	194, 179	20, 30	1
Clarithromycin	ESI+	749	30	590, 158	15, 25	1
Diclofenac	ESI+	296	16	214	32	1
Erythromycin	ESI+	734	30	158, 116	32, 40	1
Hydrochlorothiazide	ESI-	296	10	205, 269	20, 20	1
Ibuprofen	ESI-	205	25	159, 161	10, 8	5
Ketoprofen	ESI-	253	25	209	5	1
Naproxen	ESI-	229	20	170, 185	15, 5	1
Sulfamethoxazole	ESI+	254	30	156, 92,108	16, 26, 20	1
Trimethoprim	ESI+	291	39	230, 123	24, 25	1
Sulfamethoxazole d4	ESI+	258	30	96, 112, 160	20, 26,16	
Acetaminophen d4	ESI+	156	30	114, 97	15, 25	
Atenolol d7	ESI+	274	35	190, 145	20, 30	
Carbamazepine 13C6	ESI+	243	30	185, 200	35, 20	
Ibuprofen d3	ESI-	208	10	164	5	

Table S4. Recovery of PhACs from distilled water by SPE procedure and concentration of PhACs in blanks.

	Recovery of spiked samples (%)	RSD (%)	Median of blank concentrations (ng/L)	Internal standards used for recovery
Acetaminophen	104	8	<1	d4 Acetaminophen
Atenolol	106	12	<1	d7 Atenolol
Carbamazepine	111	13	<1	13C6 Carbamazepine
Clofibric acid	115	14	<1	d3 ibuprofen
Diclofenac	78	23	<1	13C6 Carbamazepine
Hydrochlorothiazide	94	26	<1	d3 ibuprofen
Ibuprofen	102	7	<5	d3 ibuprofen
Ketoprofen	93	14	1.7	d3 ibuprofen
Naproxen	89	9	<1	d3 ibuprofen
Sulfamethoxazole	107	4	<1	d4 Sulfamethoxazole
Clarithromycin	73	19	<1	d4 Sulfamethoxazole
Erythromycin	81	12	1	d4 Sulfamethoxazole
Trimethoprim	103	6	<1	d4 Sulfamethoxazole

Table S5. Concentrations (ng/L) of PhACs in WWTPeff. in cold periods (periods 2021_03_16 – 2021_05_25 and 2021_11_02 – 2022_03_29) and warm period (2021_06_15 – 2021_10_26). Red values represent ½LOQ (non-detected compounds).

Sampling date	ACET	ATEN	CARB	CLAR	DICL	ERYT	HYDR	IBUP	KETO	NAPR	SULF	TRIM	ΣPhACs
2021_03_16	9.9	87.4	459.8	860.0	1286.5	83.0	2565.0	37.1	-	321.3	424.9	156.2	6291.1
2021_03_30	9.0	77.1	521.6	1036.6	1451.3	108.4	2853.4	71.5	-	348.6	479.2	202.6	7159.3
2021_04_06	9.4	97.2	412.0	818.1	1020.1	77.5	2305.9	2.5	-	264.1	403.2	142.2	5552.2
2021_04_13	0.5	155.3	965.8	1030.4	1390.5	97.9	2223.5	2.5	-	345.2	313.3	141.2	6666.1
2021_04_26	7.2	84.7	443.0	616.1	1120.1	107.4	1331.1	2.5	-	176.3	502.7	120.2	4511.3
2021_05_25	0.5	82.0	483.4	439.7	1109.5	666.1	546.6	2.5	57.5	182.4	229.3	128.7	3928.2
2021_11_02	7.0	83.1	503.0	134.6	2021.8	102.8	1175.0	14.0	46.5	208.3	1226.3	37.8	5560.2
2021_11_16	4.4	95.4	498.7	135.7	1518.0	90.6	1101.0	10.6	42.4	177.5	1246.8	37.6	4958.7
2021_11_30	8.7	96.7	380.2	130.9	1342.7	103.6	581.6	10.8	41.8	168.3	1328.5	85.8	4279.6
2021_12_07	6.5	94.1	383.1	228.8	653.2	319.6	483.8	15.2	11.1	107.5	575.2	99.5	2977.6
2021_12_14	6.5	95.3	422.0	250.5	928.0	393.8	504.2	18.3	11.0	100.6	573.5	87.1	3390.8
2022_01_11	6.4	114.3	514.6	298.1	733.3	365.7	479.0	81.3	12.6	94.5	1108.3	134.8	3942.9
2022_01_18	10.0	114.0	528.1	247.4	1338.2	322.5	610.1	119.3	13.0	123.3	1097.9	126.8	4650.6
2022_02_01	9.4	129.1	443.5	142.9	1107.2	144.8	932.9	168.6	26.3	170.6	483.8	213.3	3972.4
2022_02_15	4.2	118.4	444.0	124.1	1055.0	139.1	895.2	175.7	23.8	158.0	735.9	160.7	4034.1
2022_02_22	6.4	86.6	397.9	120.7	1102.3	131.9	724.3	69.2	16.7	103.6	668.6	170.3	3598.5
2022_03_15	7.1	82.3	318.5	122.1	721.3	119.5	508.3	85.7	35.9	194.5	404.7	174.4	2774.3
2022_03_22	7.2	61.3	297.4	115.2	736.0	115.5	422.8	53.9	26.5	195.6	360.9	163.2	2555.5
2022_03_29	6.3	64.9	287.8	108.8	627.4	118.3	338.0	28.3	22.7	194.5	369.0	131.7	2297.7
2021_06_15	0.5	76.2	633.5	572.6	1471.3	677.4	450.7	2.5	47.8	137.0	395.9	69.6	4535.0
2021_06_22	0.5	69.7	653.8	580.8	2084.6	619.4	552.7	2.5	71.9	209.0	531.7	65.0	5441.6
2021_06_30	0.5	59.1	460.5	287.4	1287.3	321.8	567.5	2.5	55.4	227.2	218.0	36.0	3523.2
2021_07_13	2.3	56.4	409.2	81.2	838.3	98.8	591.2	2.5	13.3		221.2	26.7	2341.1
2021_07_20	5.2	81.2	545.1	71.1	1825.7	64.3	1191.9	29.7	41.0	337.3	518.9	30.3	4741.7
2021_07_28	2.2	66.8	361.2	420.4	795.0	287.2	277.4	2.5	25.8	106.8	372.6	55.9	2773.8
2021_08_03	1.7	68.4	453.0	92.8	564.5	68.0	883.6	2.5	13.0	130.9	291.8	33.2	2603.4
2021_08_10	4.2	81.9	340.5	86.5	1296.0	64.6	890.4	2.5	13.2	258.2	241.5	34.3	3313.8
2021_08_24	2.8	75.6	412.6	87.5	1313.6	78.1	853.9	25.5	28.1	172.7	560.3	26.8	3637.5

2021_08_31	4.4	93.5	368.9	103.8	1679.1	90.0	1201.2	2.5	48.9	274.3	411.7	35.8	4314.1
2021_09_07	5.0	84.8	495.6	101.1	1667.4	84.2	1013.8	20.8	17.9	167.7	735.9	33.8	4428.0
2021_09_14	8.9	93.1	626.0	226.9	1303.1	118.8	1062.3	38.0	55.5	339.7	597.8	46.9	4517.0
2021_09_21	9.5	91.6	637.6	229.3	1022.9	102.0	1296.6	12.9	48.5	220.7	729.4	48.1	4449.1
2021_09_29	6.4	79.0	572.4	231.2	1083.7	88.6	1236.9	2.5	65.8	193.7	937.9	43.2	4541.3
2021_10_05	10.4	90.3	653.7	212.7	1641.0	112.7	1312.3	13.6	47.4	210.4	1470.3	38.1	5812.9
2021_10_12	4.0	90.4	629.0	249.7	2841.9	105.0	1066.9	6.7	40.2	183.0	1241.6	41.8	6500.2
2021_10_19	10.1	91.9	613.4	240.5	1827.1	94.1	911.5	11.8	37.0	142.8	1562.2	31.9	5574.3
2021_10_26	7.8	82.0	499.4	131.3	2424.4	102.0	1048.9	2.5	45.9	171.9	1011.7	37.8	5565.6

Table S6. Concentrations (ng/L) of PhACs in WWTPeff. treated by ultrafiltration (UF) sampled at cold periods of the year (periods 2021_04_26 – 2021_05_25 and 2021_11_02 – 2022_03_29) and warm period of the year (2021_06_08 – 2021_09_29). Red values represent ½LOQ (non-detected compounds).

Sampling date	ACET	ATEN	CARB	CLAR	DICL	ERYT	HYDR	IBUP	KETO	NAPR	SULF	TRIM	ΣPhACs
2021_04_26	7.6	82.6	459.7	627.4	1306.4	105.6	1759.6	2.5	-	250.1	552.2	128.2	5281.9
2021_05_25	0.5	80.3	486.5	516.3	1351.3	697.4	555.0	2.5	44.6	183.8	259.0	120.4	4297.6
2021_11_02	7.3	82.3	503.0	132.2	1793.4	104.2	789.1	13.4	48.0	176.5	1201.5	37.7	4888.6
2021_11_16	8.7	97.8	473.7	136.1	1821.8	91.4	891.2	10.9	43.1	184.3	1271.6	37.0	5067.6
2021_11_30	8.6	99.5	380.8	125.6	1351.1	100.2	515.7	10.1	47.1	189.6	1374.8	80.1	4283.2
2021_12_14	6.5	103.6	428.1	314.9	1202.6	431.5	441.2	29.2	12.8	175.3	595.4	101.9	3843.0
2022_01_11	5.6	116.9	496.7	318.7	1373.8	420.1	538.2	84.6	10.1	111.3	1124.8	144.6	4745.4
2022_02_01	2.0	123.1	445.9	127.2	662.6	141.7	706.2	150.7	24.5	104.3	472.6	190.7	3151.5
2022_02_22	3.1	105.9	425.6	119.1	1204.6	143.0	1001.3	89.4	33.5	158.0	858.1	169.1	4310.7
2022_03_15	7.2	82.0	311.7	114.5	1056.5	111.4	408.3	85.3	36.7	212.7	373.6	154.7	2954.6
2022_03_29	6.0	57.8	263.8	120.0	951.4	127.1	264.4	21.1	22.9	233.7	384.4	114.9	2567.5
2021_06_08	4.8	64.5	653.9	100.7	2114.7	108.1	1057.1	2.5	22.1	197.4	528.7	34.1	4888.6
2021_06_15	4.4	67.5	491.4	83.6	1391.9	92.4	1114.6	2.5	43.1	182.6	382.2	30.3	3886.5
2021_06_30	0.5	56.2	420.3	319.3	1236.6	377.5	436.6	2.5	56.7	224.4	263.1	40.2	3433.9
2021_07_13	1.2	58.8	399.9	82.1	1259.6	94.1	649.4	2.5	13.7	144.7	245.0	26.5	2977.5
2021_07_20	4.1	74.9	419.1	93.9	1389.9	85.9	642.5	2.5	17.7	124.0	534.8	27.0	3416.3
2021_07_28	0.5	69.8	395.0	464.0	942.4	281.7	296.6	2.5	25.7	118.1	397.9	63.3	3057.5
2021_08_24	3.4	74.6	383.9	82.5	1266.3	74.7	771.3	2.5	37.1	176.7	506.1	25.0	3404.1
2021_09_21	7.8	86.5	576.2	227.8	1148.3	104.1	1273.7	7.9	46.4	225.3	675.6	49.6	4429.2
2021_09_29	7.9	72.6	532.2	218.4	1038.1	89.0	816.7	2.5	39.8	163.4	860.1	41.4	3882.1

Table S7. Concentrations (ng/L) of PhACs in WWTPeff. treated by Granular Activated Carbon (GAC) sampled at cold periods of the year (periods 2021_03_16 – 2021_04_13) and warm period of the year (2021_06_30 – 2021_10_12). Red values represent ½LOQ (non-detected compounds).

Sampling date	ACET	ATEN	CARB	CLAR	DICL	ERYT	HYDR	IBUP	KETO	NAPR	SULF	TRIM	ΣPhACs
2021_03_16	3.3	11.7	112.2	657.3	1206.4	59.7	185.3	25.6	-	100.9	308.7	31.4	2702.5
2021_03_30	7.8	38.1	330.0	717.9	1501.2	73.5	609.2	2.5	-	153.2	445.6	69.7	3948.7
2021_04_06	5.0	32.3	333.0	542.1	1024.9	53.4	442.1	2.5	-	92.5	396.0	48.8	2972.6
2021_04_13	6.9	68.3	459.8	657.0	1279.6	64.0	1102.4	2.5	-	223.2	440.4	97.6	4401.7
2021_06_30	0.5	2.6	74.5	221.7	633.9	269.8	32.9	2.5	20.1	58.7	137.5	3.7	1458.4
2021_07_13	1.5	12.2	206.5	73.4	1263.4	89.8	151.7	2.5	15.6	19.2	231.0	8.8	2075.6
2021_07_20	4.3	56.1	467.7	110.4	1979.7	105.8	765.7	2.5	42.9	274.0	690.6	16.5	4516.2
2021_08_31	3.8	89.0	335.6	102.4	1545.3	92.6	1011.3	2.5	25.1	229.6	395.7	33.7	3866.6
2021_09_29	6.5	70.6	549.4	220.6	839.8	89.8	1159.2	2.5	63.4	160.7	870.3	39.1	4071.9
2021_10_05	7.8	74.3	659.4	210.2	1201.6	112.0	887.4	11.7	30.9	159.9	1305.5	32.7	4693.4
2021_10_12	7.6	75.8	570.4	253.2	1724.9	106.9	941.7	7.2	40.0	151.9	1175.7	36.2	5091.5

Table S8. Concentrations (ng/L) of PhACs in WWTPeff. treated by Granular Activated Carbon with UV irradiation (GAC + UV) sampled at cold periods of the year (periods 2021_03_16 – 2021_04_26 and 2021_11_02 – 2022_03_29) and warm period of the year (2021_06_30 – 2021_10_26). Red values represent ½LOQ (non-detected compounds).

Sampling date	ACET	ATEN	CARB	CLAR	DICL	ERYT	HYDR	IBUP	KETO	NAPR	SULF	TRIM	ΣPhACs
2021_03_16	6.5	9.7	106.0	549.4	564.1	55.9	133.7	21.2	-	89.6	180.5	25.7	1742.3
2021_03_30	13.5	37.2	302.6	729.1	331.8	72.2	597.7	2.5	-	155.6	245.2	70.3	2557.7
2021_04_06	14.5	31.8	283.2	618.5	55.2	62.7	339.6	2.5	-	82.6	134.5	50.9	1676.0
2021_04_13	14.3	70.4	453.1	631.4	634.0	63.3	1152.1	2.5	-	222.3	288.3	99.8	3631.5
2021_04_26	12.3	68.8	440.7	597.7	802.6	94.1	975.0	2.5	-	170.3	414.8	101.9	3680.7
2021_11_02	6.9	30.8	285.4	117.0	904.2	93.5	253.6	2.5	2.0	90.8	553.5	14.5	2354.7
2021_11_16	8.1	51.4	340.1	126.3	541.5	87.6	335.3	2.5	2.1	99.7	999.5	19.5	2613.6
2021_11_30	8.7	71.6	310.1	125.2	813.4	97.7	480.8	2.5	4.5	156.9	1126.4	53.9	3251.7
2021_12_14	4.5	39.0	210.1	181.4	760.3	246.4	99.5	5.3	0.5	74.5	338.3	35.9	1995.7
2022_01_11	9.8	92.5	463.2	245.3	661.0	327.3	393.3	79.4	0.5	115.8	958.3	112.1	3458.5
2022_02_01	17.8	114.6	431.4	129.5	791.6	137.6	661.8	156.9	0.5	130.1	354.2	175.0	3101.0
2022_02_22	11.7	75.3	371.0	117.5	915.0	133.7	531.6	64.9	0.5	115.4	471.0	125.1	2932.7
2022_03_15	8.0	48.5	228.9	110.8	208.9	110.0	211.2	36.0	1.7	93.6	283.0	94.4	1435.0
2022_03_29	7.8	35.8	213.0	100.7	287.0	112.2	140.7	9.1	0.5	138.7	239.6	70.1	1355.2
2021_06_30	6.2	4.6	91.0	221.5	362.2	281.7	34.7	2.5	0.5	59.0	135.4	4.3	1203.6
2021_07_28	7.0	29.6	239.7	399.4	320.2	283.2	134.7	2.5	0.5	76.6	297.0	32.7	1823.1
2021_08_03	6.1	23.5	229.0	76.8	172.8	57.6	303.7	2.5	24.3	62.5	265.6	10.0	1234.4
2021_08_10	3.0	39.8	240.0	87.4	591.9	67.8	489.5	2.5	18.1	100.3	223.1	19.4	1882.8
2021_08_24	7.3	56.8	353.8	81.4	425.2	70.9	586.9	2.5	24.5	113.5	476.2	21.6	2220.6
2021_09_21	12.3	77.2	537.8	204.5	876.6	100.3	1144.0	8.6	3.8	254.3	443.6	44.4	3707.4
2021_10_26	4.7	8.5	90.5	97.9	437.8	77.8	43.9	2.5	0.5	43.0	349.8	4.3	1161.2

Table S9. Concentrations (ng/L) of PhACs in WWTPeff. treated by Nanofiltration (NF, in the setup UF+GAC+UV+NF) sampled at cold periods of the year (periods 2021_03_30 – 2021_04_26 and 2021_11_02 – 2022_03_29) and warm period of the year (2021_05_25 – 2021_09_29). Red values represent ½LOQ (non-detected compounds).

Sampling date	ACET	ATEN	CARB	CLAR	DICL	ERYT	HYDR	IBUP	KETO	NAPR	SULF	TRIM	ΣPhACs
2021_03_30	9.4	3.6	115.9	2.3	50.3	1.2	776.3	2.5	-	20.6	102.4	16.1	1100.6
2021_04_26	0.5	0.5	55.1	2.3	32.5	2.6	130.2	2.5	-	28.1	60.3	6.5	321.1
2021_11_02	2.2	3.2	68.5	2.4	38.6	2.4	228.1	2.5	0.5	14.8	209.0	2.2	574.4
2021_11_16	3.9	4.6	69.1	1.8	36.6	2.3	229.2	2.5	0.5	14.6	225.3	2.3	592.7
2021_11_30	1.7	4.7	63.5	0.5	27.3	0.5	240.0	2.5	0.5	20.2	260.5	7.2	629.1
2021_12_14	1.7	24.4	102.5	3.4	23.3	3.6	219.7	2.5	0.5	23.6	165.1	13.4	583.7
2022_01_11	2.9	4.4	75.3	1.3	22.8	2.9	210.3	2.5	0.5	7.0	151.4	8.9	490.2
2022_02_08	0.5	4.9	64.4	3.1	24.7	6.4	244.6	2.5	0.5	8.6	166.3	9.9	536.4
2022_02_22	4.2	3.8	119.0	3.8	144.5	9.0	279.8	2.5	0.5	15.1	145.8	10.9	738.9
2022_03_15	2.2	1.4	56.7	1.1	31.1	2.4	138.9	2.5	0.5	17.3	38.3	11.1	303.5
2022_03_29	4.3	2.1	87.6	2.7	40.0	4.4	143.1	2.5	0.5	44.3	63.1	28.5	423.1
2021_05_25	1.9	1.9	66.1	1.6	22.0	3.4	208.7	2.5	0.5	12.5	25.3	5.1	351.5
2021_06_15	1.3	1.8	83.1	1.0	73.4	1.3	143.4	2.5	0.5	9.7	52.5	2.4	372.9
2021_06_30	2.5	1.9	60.1	1.6	34.7	1.6	102.5	2.5	2.7	18.2	28.8	1.1	258.2
2021_07_13	1.2	2.0	64.5	0.5	25.2	1.3	220.6	2.5	0.5	12.9	58.2	1.9	391.3
2021_07_28	0.5	2.6	66.4	0.5	29.9	0.5	140.1	2.5	0.5	14.0	78.5	3.5	339.5
2021_08_03	1.3	2.6	75.4	0.5	42.6	0.5	264.9	2.5	1.9	14.7	64.4	2.0	473.3
2021_08_24	1.8	3.3	75.4	0.5	24.1	0.5	288.3	2.5	0.5	7.4	104.1	2.6	511.0
2021_09_07	3.1	4.0	96.4	0.5	39.6	1.7	371.2	2.5	3.9	6.5	181.2	2.9	713.5
2021_09_29	2.2	0.5	36.4	0.5	14.1	0.5	100.4	2.5	0.5	4.2	129.8	0.5	292.1

Table S10. Concentrations (ng/L) of PhACs in WWTPeff. treated by Ozone (O_3, in the setup UF+O_3) sampled at cold periods of the year (periods 2021_11_16 – 2022_03_29) and warm period of the year (2021_06_30 – 2021_10_05). Red values represent ½LOQ (non-detected compounds).

Sampling date	ACET	ATEN	CARB	CLAR	DICL	ERYT	HYDR	IBUP	KETO	NAPR	SULF	TRIM	ΣPhACs
2021_06_30	1.8	0.5	0.5	3.1	0.5	2.0	0.5	2.5	0.5	0.5	6.6	0.5	19.5
2021_07_20	0.5	0.5	0.5	0.5	0.5	1.6	0.5	2.5	1.6	0.5	0.5	0.5	10.2
2021_08_17	0.5	0.5	0.5	0.5	0.5	0.5	0.5	2.5	2.0	0.5	0.5	0.5	9.5
2021_09_21	0.5	0.5	0.5	3.5	2.0	7.9	0.5	2.5	9.3	0.5	3.9	0.5	32.1
2021_09_29	0.5	0.5	0.5	0.5	0.5	0.5	0.5	2.5	0.5	0.5	0.5	0.5	8.0
2021_10_05	0.5	0.5	0.5	0.5	0.5	2.0	0.5	2.5	7.2	0.5	7.6	0.5	23.3
2021_11_16	0.5	0.5	0.5	0.5	4.3	0.5	0.5	2.5	9.5	0.5	5.2	0.5	25.5
2021_12_14	0.5	0.5	0.5	0.5	0.5	0.5	0.5	2.5	0.5	2.4	0.5	0.5	9.9
2022_01_11	0.5	0.5	0.5	1.8	0.5	3.3	0.5	10.1	0.5	0.5	1.1	0.5	20.3
2022_02_01	0.5	0.5	0.5	0.5	0.5	2.7	0.5	31.4	1.8	0.5	0.5	0.5	40.4
2022_02_22	0.5	0.5	0.5	0.5	10.2	0.5	0.5	11.6	2.7	0.5	3.1	0.5	31.6
2022_03_15	1.5	3.6	0.5	0.5	23.5	2.8	0.5	20.5	10.0	1.3	4.6	0.5	69.8
2022_03_29	2.0	4.0	0.5	0.5	17.2	3.2	0.5	7.8	1.9	0.5	10.6	0.5	49.2

Table S11. Concentrations (ng/L) of PhACs in WWTPeff. treated by Reverse osmosis (RO, in the setup UF+GAC+UV+RO) sampled at cold periods of the year (periods 2021_03_30 – 2021_04_26 and 2021_11_16 – 2022_03_29) and warm period of the year (2021_05_25 – 2021_10_12). Red values represent ½LOQ (non-detected compounds).

Sampling date	ACET	ATEN	CARB	CLAR	DICL	ERYT	HYDR	IBUP	KETO	NAPR	SULF	TRIM	ΣPhACs
2021_03_30	0.5	0.5	2.1	0.5	3.7	0.5	6.3	2.5	-	0.5	2.0	0.5	19.6
2021_04_26	0.5	0.5	0.5	0.5	0.5	0.5	9.0	2.5	-	0.5	0.5	0.5	16.0
2021_11_02	0.5	0.5	0.5	0.5	2.2	0.5	1.8	2.5	0.5	0.5	0.5	0.5	11.0
2021_11_16	0.5	0.5	0.5	0.5	1.8	0.5	3.1	2.5	0.5	0.5	1.9	0.5	13.3
2021_11_30	0.5	0.5	0.5	0.5	1.9	0.5	1.4	2.5	0.5	0.5	0.5	0.5	10.3
2021_12_14	0.5	1.2	1.7	0.5	2.8	0.5	2.6	2.5	0.5		2.0	0.5	15.3
2022_01_11	0.5	0.5	1.3	0.5	1.8	0.5	0.5	2.5	0.5	0.5	1.2	0.5	10.8
2022_02_01	0.5	0.5	0.5	0.5	0.5	0.5	0.5	2.5	0.5	0.5	1.6	0.5	9.1
2022_02_22	0.5	0.5	1.6	0.5	7.6	0.5	0.5	2.5	0.5	2.3	0.5	0.5	18.0
2022_03_15	0.5	0.5	0.5	0.5	4.3	1.1	0.5	2.5	0.5	1.9	0.5	0.5	13.8
2022_03_29	0.5	0.5	0.5	0.5	1.6	0.5	0.5	2.5	0.5	1.4	0.5	0.5	10.0
2021_05_25	0.5	0.5	0.5	0.5	0.5	0.5	0.5	2.5	0.5	0.5	0.5	0.5	8.0
2021_06_15	0.5	0.5	0.5	0.5	0.5	0.5	1.4	2.5	0.5	0.5	0.5	0.5	8.9
2021_06_30	0.5	0.5	0.5	0.5	0.5	0.5	1.9	2.5	0.5	0.5	0.5	0.5	9.4
2021_07_13	0.5	0.5	0.5	0.5	0.5	0.5	1.5	2.5	0.5	0.5	0.5	0.5	9.0
2021_07_28	0.5	0.5	0.5	0.5	0.5	0.5	1.6	2.5	0.5	0.5	0.5	0.5	9.1
2021_08_24	0.5	1.3	0.5	0.5	1.3	0.5	8.1	2.5	0.5	0.5	0.5	0.5	17.2
2021_09_29	0.5	0.5	0.5	0.5	1.9	0.5	0.5	2.5	0.5	0.5	0.5	0.5	9.4

Table S12. Equations for regression line and values corresponding to various centile values for environmental exposure distribution (EEDs) of reported determined environmental concentration in WWTPeff., and WWTPeff. samples treated with quaternary treatment technologies. Additionally, predicted percent exceedance (PPE) is given for the predicted no-effect concentration (PNEC) and the predicted no-effect concentration for antibiotic resistance (PNEC-AR).

n – number of samples; NE – No exceedance of PNEC or PNEC-AR value

Acetaminophen

Season	Technology	n	r2	Slope	Intercept	Centile Value (ng/L)									PNEC: 500 ng/L
						1	5	10	25	50	75	90	95	99	PPE of PNEC (%)
Cold	WWTPeff.	19	0.63	1.91	-1.45	0.3	0.8	1.2	2.6	5.8	13.0	27.0	41.9	95.4	0.0
	GAC	4	insufficient number of samples for EED												NE
	GAC+UV	14	0.97	5.15	-5.08	3.4	4.6	5.5	7.2	9.7	13.1	17.1	20.2	27.3	0.0
	UF	11	0.71	1.89	-1.25	0.3	0.6	1.0	2.0	4.6	10.5	22.0	34.3	78.9	0.0
	UF+GAC+UV+NF	11	0.94	2.29	-0.88	0.2	0.5	0.7	1.2	2.4	4.8	8.8	12.6	25.1	0.0
	UF+O3	7	>20 % samples below LOQ												NE
	UF+GAC+UV+RO	10	>20 % samples below LOQ												NE
Warm	WWTPeff.	18	0.95	2.11	-1.16	0.3	0.6	0.9	1.7	3.5	7.4	14.3	21.2	44.6	0.0
	GAC	7	0.84	1.65	-0.87	0.1	0.3	0.6	1.3	3.3	8.6	20.0	33.1	85.6	0.0
	GAC+UV	7	0.92	4.06	-3.20	1.6	2.4	3.0	4.2	6.2	9.0	12.7	15.7	23.0	0.0
	UF	9	0.92	1.80	-0.80	0.1	0.3	0.5	1.2	2.8	6.6	14.2	22.6	54.0	0.0
	UF+GAC+UV+NF	9	0.99	5.08	-1.20	0.6	0.8	1.0	1.3	1.7	2.3	3.1	3.6	4.9	0.0
	UF+O3	6	>20 % samples below LOQ												NE
	UF+GAC+UV+RO	8	>20 % samples below LOQ												NE

Atenolol

Season	Technology	n	r2	Slope	Intercept	Centile Value (ng/L)									PNEC: 100,000 ng/L
						1	5	10	25	50	75	90	95	99	PPE of PNEC (%)
Cold	WWTPeff.	19	0.95	8.85	-17.44	51.0	60.9	66.9	78.4	93.4	111.3	130.4	143.3	171.1	0.0
	GAC	4	insufficient number of samples for EED												NE
	GAC+UV	14	0.88	3.03	-5.09	8.2	13.7	18.1	28.7	48.0	80.1	127.1	167.5	281.2	0.0
	UF	11	0.91	8.65	-16.99	49.5	59.4	65.4	76.8	92.0	110.0	129.3	142.5	170.8	0.0
	UF+GAC+UV+NF	11	0.94	2.29	-0.88	0.2	0.5	0.7	1.2	2.4	4.8	8.8	12.6	25.1	0.0
	UF+O3	7	>20 % samples below LOQ												NE
	UF+GAC+UV+RO	10	>20 % samples below LOQ												NE
Warm	WWTPeff.	18	0.92	12.56	-23.82	51.4	58.2	62.2	69.5	78.7	89.0	99.5	106.4	120.5	0.0
	GAC	7	0.73	1.17	-1.80	0.4	1.4	2.8	9.2	34.9	131.9	436.7	893.8	3426.2	0.0
	GAC+UV	7	0.94	1.74	-2.40	1.1	2.7	4.4	9.9	24.1	58.9	131.6	212.9	524.7	0.0
	UF	9	0.97	14.06	-25.85	47.1	52.7	55.9	61.7	69.0	77.0	85.1	90.3	100.9	0.0
	UF+GAC+UV+NF	9	0.76	2.77	-0.91	0.3	0.5	0.7	1.2	2.1	3.7	6.2	8.4	14.8	0.0
	UF+O3	6	>20 % samples below LOQ												NE
	UF+GAC+UV+RO	8	>20 % samples below LOQ												NE

Carbamazepine

Season	Technology	n	r2	Slope	Intercept	Centile Value (ng/L)									PNEC: 10 ng/L
						1	5	10	25	50	75	90	95	99	PPE of PNEC (%)
Cold	WWTPeff.	19	0.85	7.18	-19.00	209.6	260.8	293.0	356.0	442.0	548.7	666.6	748.9	931.9	100.0
	GAC	4	insufficient number of samples for EED												
	GAC+UV	14	0.88	4.64	-11.46	93.5	131.2	157.1	212.4	297.0	415.1	561.3	672.3	943.1	100.0
	UF	11	0.82	8.54	-22.37	222.9	267.9	295.5	348.1	417.6	500.9	590.1	650.8	782.2	100.0
	UF+GAC+UV+NF	11	0.93	6.80	-12.83	35.0	44.1	49.9	61.3	77.0	96.8	118.8	134.4	169.3	100.0
	UF+O3	7	>20 % samples below LOQ												NE
	UF+GAC+UV+RO	10	>20 % samples below LOQ												NE
Warm	WWTPeff.	18	0.92	8.67	-23.46	274.1	328.4	361.7	425.0	508.4	608.1	714.5	786.9	943.1	100.0
	GAC	7	0.83	2.15	-5.43	27.9	57.9	85.5	163.8	337.5	695.5	1333.2	1967.9	4085.6	100.0
	GAC+UV	7	0.91	2.64	-6.14	28.1	50.9	69.9	118.8	214.1	386.0	656.1	901.2	1634.6	100.0
	UF	9	0.91	9.44	-25.19	264.6	312.5	341.5	396.0	466.8	550.3	638.2	697.3	823.4	100.0
	UF+GAC+UV+NF	9	0.84	6.24	-11.44	28.8	37.1	42.4	53.0	68.0	87.2	109.1	124.8	160.5	100.0
	UF+O3	6	>20 % samples below LOQ												NE
	UF+GAC+UV+RO	8	>20 % samples below LOQ												NE

Clarithromycin

Season	Technology	n	r2	Slope	Intercept	Centile Value (ng/L)									PNEC: 20 ng/L	PNEC-AR: 250 ng/L
						1	5	10	25	50	75	90	95	99	PPE of PNEC (%)	PPE of PNEC-AR (%)
Cold	WWTPeff.	19	0.87	2.30	-5.54	25.1	49.7	71.5	131.4	258.2	507.7	932.9	1342.7	2658.4	99.5	51.3
	GAC	4	insufficient number of samples for EED													
	GAC+UV	14	0.83	2.28	-5.39	22.1	44.0	63.5	117.3	232.0	458.7	847.4	1223.5	2436.7	99.2	47.0
	UF	11	0.79	2.65	-6.07	25.8	46.7	64.0	108.4	194.8	349.8	592.7	812.5	1468.4	99.6	38.7
	UF+GAC+UV+NF	11	0.86	3.03	-0.91	0.3	0.6	0.8	1.2	2.0	3.3	5.3	7.0	11.7	38.7	0.0
	UF+O3	7	>20 % samples below LOQ												NE	NE
	UF+GAC+UV+RO	10	>20 % samples below LOQ												NE	NE
Warm	WWTPeff.	18	0.93	2.91	-6.55	28.3	48.6	64.8	104.8	178.7	304.8	492.7	656.9	1126.6	99.7	33.6
	GAC	7	0.87	3.48	-7.63	33.3	52.3	66.5	99.3	155.1	242.3	361.9	460.2	722.1	99.9	23.5
	GAC+UV	7	0.88	2.66	-5.70	18.5	33.4	45.7	77.2	138.3	247.7	418.6	573.0	1032.5	98.7	24.7
	UF	9	0.87	2.62	-5.70	19.5	35.4	48.8	83.2	150.4	272.2	464.1	638.7	1162.6	98.9	28.2
	UF+GAC+UV+NF	9	>20 % samples below LOQ												NE	NE
	UF+O3	6	>20 % samples below LOQ												NE	NE
	UF+GAC+UV+RO	8	>20 % samples below LOQ												NE	NE

Diclofenac

Season	Technology	n	r2	Slope	Intercept	Centile Value (ng/L)									PNEC: 10 ng/L
						1	5	10	25	50	75	90	95	99	PPE of PNEC (%)
Cold	WWTPeff.	19	0.96	6.29	-19.06	455.7	584.7	667.9	834.0	1067.5	1366.4	1706.3	1948.9	2500.9	100.0
	GAC	4	insufficient number of samples for EED												
	GAC+UV	14	0.75	2.22	-5.96	43.7	88.6	129.3	243.0	489.8	987.2	1855.3	2706.3	5494.9	100.0
	UF	11	0.90	6.50	-20.09	542.0	690.2	785.0	973.5	1236.5	1570.5	1947.5	2215.2	2820.6	100.0
	UF+GAC+UV+NF	11	0.76	3.24	-5.06	7.0	11.3	14.6	22.5	36.4	58.8	90.5	117.2	190.3	100.0
	UF+O3	7	>20 % samples below LOQ												
	UF+GAC+UV+RO	10	0.96	2.33	-0.75	0.2	0.4	0.6	1.1	2.1	4.1	7.4	10.6	20.8	77.2
Warm	WWTPeff.	18	0.97	4.95	-15.55	471.5	647.5	766.8	1017.1	1392.1	1905.4	2527.5	2993.0	4110.0	100.0
	GAC	7	0.96	4.41	-13.63	365.2	521.2	630.1	865.1	1230.2	1749.5	2401.9	2903.5	4144.2	100.0
	GAC+UV	7	0.95	3.50	-9.15	88.7	138.9	176.3	262.9	409.6	638.4	951.6	1208.5	1891.9	100.0
	UF	9	0.86	7.67	-23.83	635.5	779.8	869.6	1043.4	1277.5	1564.2	1876.8	2093.0	2568.0	100.0
	UF+GAC+UV+NF	9	0.96	3.94	-5.85	7.9	11.7	14.5	20.7	30.7	45.5	65.0	80.3	119.7	100.0
	UF+O3	6	>20 % samples below LOQ												
	UF+GAC+UV+RO	8	>20 % samples below LOQ												

Erythromycin

Season	Technology	n	r2	Slope	Intercept	Centile Value (ng/L)									PNEC: 200 ng/L	PNEC-AR: 1000 ng/L
						1	5	10	25	50	75	90	95	99	PPE of PNEC (%)	PPE of PNEC-AR (%)
Cold	WWTPeff.	19	0.83	2.98	-6.50	25.2	42.7	56.6	90.5	152.5	256.9	410.9	544.2	921.8	36.3	0.8
	GAC	4	insufficient number of samples for EED												NE	NE
	GAC+UV	14	0.91	3.76	-7.62	25.5	38.7	48.3	70.0	105.8	159.9	231.8	289.5	439.3	14.9	0.0
	UF	11	0.79	2.40	-5.35	18.3	35.2	49.9	89.4	171.0	326.9	585.8	830.6	1598.7	43.5	3.3
	UF+GAC+UV+NF	11	0.91	2.38	-1.05	0.3	0.6	0.8	1.4	2.7	5.3	9.5	13.5	26.0	0.0	0.0
	UF+O3	7	>20 % samples below LOQ												NE	NE
	UF+GAC+UV+RO	10	>20 % samples below LOQ												NE	NE
Warm	WWTPeff.	18	0.78	2.45	-5.16	14.4	27.3	38.4	68.0	128.3	242.0	428.3	602.9	1144.6	31.8	1.5
	GAC	7	0.59	3.99	-8.28	31.0	46.0	56.7	80.6	118.9	175.5	249.1	307.3	455.4	18.4	0.0
	GAC+UV	7	0.82	2.43	-4.94	11.9	22.8	32.1	57.1	108.1	204.8	364.0	513.5	979.4	25.8	0.9
	UF	9	0.73	2.82	-5.87	18.2	31.7	42.7	70.2	121.9	211.6	347.7	468.0	817.2	27.2	0.5
	UF+GAC+UV+NF	9	>20 % samples below LOQ												NE	NE
	UF+O3	6	>20 % samples below LOQ												NE	NE
	UF+GAC+UV+RO	8	>20 % samples below LOQ												NE	NE

Hydrochlorothiazide

Season	Technology	n	r2	Slope	Intercept	Centile Value (ng/L)									PNEC: 56,200 ng/L
						1	5	10	25	50	75	90	95	99	PPE of PNEC (%)
Cold	WWTPeff.	19	0.93	2.96	-8.70	141.8	240.9	319.5	512.3	865.5	1462.5	2344.8	3110.3	5284.1	0.0
	GAC	4	insufficient number of samples for EED												NE
	GAC+UV	14	0.99	2.66	-6.78	47.4	85.5	117.1	198.1	355.2	636.9	1077.3	1475.4	2661.4	0.0
	UF	11	0.96	3.72	-10.41	149.6	228.2	285.8	416.2	632.0	959.6	1397.6	1750.2	2669.2	0.0
	UF+GAC+UV+NF	11	0.77	3.53	-8.30	49.5	77.3	98.0	145.7	226.3	351.6	522.6	662.6	1034.1	0.0
	UF+O3	7	>20 % samples below LOQ												NE
	UF+GAC+UV+RO	10	>20 % samples below LOQ												NE
Warm	WWTPeff.	18	0.86	4.44	-13.00	253.7	361.3	436.2	597.7	848.1	1203.5	1649.0	1991.0	2835.5	0.0
	GAC	7	0.73	1.15	-3.06	4.3	16.9	34.9	117.1	450.1	1729.9	5811.3	12000.7	46770.9	0.8
	GAC+UV	7	0.95	1.33	-3.11	3.9	12.5	23.4	66.8	213.9	685.1	1953.4	3656.8	11855.5	0.1
	UF	9	0.94	3.93	-11.22	183.7	273.9	338.9	483.8	718.6	1067.2	1523.5	1885.2	2811.2	0.0
	UF+GAC+UV+NF	9	0.97	4.00	-9.08	48.8	72.3	89.1	126.3	186.3	274.6	389.4	479.9	710.4	0.0
	UF+O3	6	>20 % samples below LOQ												NE
	UF+GAC+UV+RO	8	>20 % samples below LOQ												NE

Ibuprofen

Season	Technology	n	r2	Slope	Intercept	Centile Value (ng/L) 1	5	10	25	50	75	90	95	99	PNEC: 10 ng/L PPE of PNEC (%)
Cold	WWTPeff.	19	>20 % samples below LOQ												
	GAC	4	insufficient number of samples for EED												
	GAC+UV	14	>20 % samples below LOQ												
	UF	11	>20 % samples below LOQ												
	UF+GAC+UV+NF	11	>20 % samples below LOQ												NE
	UF+O3	7	>20 % samples below LOQ												
	UF+GAC+UV+RO	10	>20 % samples below LOQ												NE
Warm	WWTPeff.	18	>20 % samples below LOQ												
	GAC	7	>20 % samples below LOQ												
	GAC+UV	7	>20 % samples below LOQ												NE
	UF	9	>20 % samples below LOQ												NE
	UF+GAC+UV+NF	9	>20 % samples below LOQ												NE
	UF+O3	6	>20 % samples below LOQ												NE
	UF+GAC+UV+RO	8	>20 % samples below LOQ												NE

Ketoprofen

Season	Technology	n	r2	Slope	Intercept	Centile Value (ng/L) 1	5	10	25	50	75	90	95	99	PNEC: 1,240 ng/L PPE of PNEC (%)
Cold	WWTPeff.	14	0.96	3.45	-4.76	5.1	8.0	10.2	15.3	24.0	37.7	56.6	72.1	113.7	0.0
	GAC	0	no samples												
	GAC+UV	9	>20 % samples below LOQ												
	UF	10	0.87	3.22	-4.69	5.4	8.9	11.5	17.8	28.8	46.6	72.0	93.3	152.0	0.0
	UF+GAC+UV+NF	9	>20 % samples below LOQ												NE
	UF+O3	7	>20 % samples below LOQ												NE
	UF+GAC+UV+RO	8	>20 % samples below LOQ												NE
Warm	WWTPeff.	18	0.89	3.42	-5.29	7.3	11.6	14.8	22.3	35.1	55.2	83.1	106.1	167.8	0.0
	GAC	7	0.99	3.78	-5.62	7.5	11.3	14.1	20.4	30.8	46.5	67.4	84.1	127.4	0.0
	GAC+UV	7	>20 % samples below LOQ												NE
	UF	9	0.96	3.83	-5.69	7.5	11.4	14.1	20.4	30.5	45.8	66.0	82.1	123.6	0.0
	UF+GAC+UV+NF	9	>20 % samples below LOQ												NE
	UF+O3	6	>20 % samples below LOQ												NE
	UF+GAC+UV+RO	8	>20 % samples below LOQ												NE

Naproxen

Season	Technology	n	r2	Slope	Intercept	Centile Value (ng/L) 1	5	10	25	50	75	90	95	99	PNEC: 36,700 ng/L PPE of PNEC (%)
Cold	WWTPeff.	19	0.95	4.97	-11.19	60.6	83.1	98.3	130.2	177.9	243.1	322.0	381.0	522.3	0.0
	GAC	4	insufficient number of samples for EED												NE
	GAC+UV	14	0.97	6.20	-12.86	49.8	64.2	73.5	92.0	118.2	151.9	190.2	217.7	280.4	0.0
	UF	11	0.90	6.72	-15.06	78.6	99.2	112.4	138.4	174.4	219.7	270.5	306.4	387.0	0.0
	UF+GAC+UV+NF	11	0.95	3.67	-4.55	4.0	6.2	7.7	11.3	17.3	26.4	38.6	48.5	74.4	0.0
	UF+O3	7	>20 % samples below LOQ												NE
	UF+GAC+UV+RO	10	>20 % samples below LOQ												NE
Warm	WWTPeff.	17	0.98	6.25	-14.30	82.8	106.4	121.7	152.2	195.2	250.2	313.0	357.9	460.1	0.0
	GAC	7	0.82	1.77	-3.66	5.6	13.7	21.9	48.2	115.7	277.6	610.3	977.9	2368.3	0.0
	GAC+UV	7	0.92	3.01	-5.83	14.6	24.5	32.4	51.5	86.1	144.2	229.2	302.5	509.1	0.0
	UF	9	0.90	6.72	-15.06	78.6	99.2	112.4	138.4	174.4	219.7	270.5	306.4	387.0	0.0
	UF+GAC+UV+NF	9	0.95	3.67	-4.55	4.0	6.2	7.7	11.3	17.3	26.4	38.6	48.5	74.4	0.0
	UF+O3	6	>20 % samples below LOQ												NE
	UF+GAC+UV+RO	8	>20 % samples below LOQ												NE

Sulfamethoxazole

Season	Technology	n	r2	Slope	Intercept	Centile Value (ng/L) 1	5	10	25	50	75	90	95	99	PNEC: 2,400 ng/L PPE of PNEC (%)	PNEC-AR: 16,000 ng/L PPE of PNEC-AR (%)
Cold	WWTPeff.	19	0.95	3.83	-10.59	143.2	215.6	268.2	386.2	579.0	868.2	1250.1	1554.9	2341.3	0.9	0.0
	GAC	4	insufficient number of samples for EED												NE	NE
	GAC+UV	14	0.96	3.03	-7.83	65.8	110.5	145.7	231.1	385.9	644.5	1022.4	1347.6	2262.4	0.8	0.0
	UF	11	0.95	3.28	-9.25	130.1	210.0	271.1	415.4	667.5	1072.4	1643.3	2121.5	3425.4	3.4	0.0
	UF+GAC+UV+NF	11	0.92	3.01	-6.30	20.9	35.3	46.6	74.2	124.5	208.7	332.2	438.8	739.7	0.0	0.0
	UF+O3	7	>20 % samples below LOQ												NE	NE
	UF+GAC+UV+RO	10	>20 % samples below LOQ												NE	NE
Warm	WWTPeff.	18	0.98	3.22	-8.85	105.8	172.2	223.3	344.6	558.2	904.2	1395.6	1809.5	2945.7	2.1	0.0
	GAC	7	0.95	2.08	-5.67	40.3	85.6	128.0	250.7	529.0	1116.2	2185.6	3267.6	6947.9	8.6	0.1
	GAC+UV	7	0.95	4.11	-10.13	79.0	115.8	141.9	199.4	290.9	424.3	596.2	730.7	1070.2	0.0	0.0
	UF	9	0.96	4.39	-11.55	126.5	180.9	218.9	301.1	429.0	611.2	840.6	1017.2	1454.7	0.1	0.0
	UF+GAC+UV+NF	9	0.98	2.87	-5.24	10.4	17.9	24.0	39.1	67.1	115.2	187.5	251.0	433.5	0.0	0.0
	UF+O3	6	>20 % samples below LOQ												NE	NE
	UF+GAC+UV+RO	8	>20 % samples below LOQ												NE	NE

Trimethoprim

Season	Technology	n	r2	Slope	Intercept	Centile Value (ng/L)									PNEC: 15,700 ng/L	PNEC-AR: 500 ng/L
						1	5	10	25	50	75	90	95	99	PPE of PNEC (%)	PPE of PNEC-AR (%)
Cold	WWTPeff.	19	0.81	3.85	-8.02	30.1	45.3	56.3	81.0	121.2	181.5	261.0	324.3	487.6	0.0	0.9
	GAC	4	insufficient number of samples for EED												NE	NE
	GAC+UV	14	0.95	2.61	-4.64	7.7	14.1	19.4	33.2	60.3	109.3	186.7	257.3	469.6	0.0	0.8
	UF	11	0.86	3.21	-6.47	19.5	31.8	41.3	63.8	103.5	167.9	259.5	336.7	549.0	0.0	1.4
	UF+GAC+UV+NF	11	0.91	2.41	-2.23	0.9	1.8	2.5	4.4	8.4	16.1	28.7	40.7	78.0	0.0	0.0
	UF+O3	7	>20 % samples below LOQ												NE	NE
	UF+GAC+UV+RO	10	>20 % samples below LOQ												NE	NE
Warm	WWTPeff.	18	0.95	7.21	-11.51	18.7	23.3	26.1	31.7	39.3	48.8	59.2	66.5	82.7	0.0	0.0
	GAC	7	0.83	1.85	-2.36	1.0	2.4	3.8	8.1	18.8	43.6	92.8	145.9	340.9	0.0	0.4
	GAC+UV	7	0.98	2.14	-2.52	1.2	2.6	3.8	7.3	15.2	31.5	60.5	89.6	186.7	0.0	0.1
	UF	9	0.95	5.83	-9.07	14.3	18.7	21.6	27.4	35.8	46.7	59.4	68.5	89.7	0.0	0.0
	UF+GAC+UV+NF	9	0.90	2.64	-0.83	0.3	0.5	0.7	1.1	2.1	3.7	6.3	8.7	15.7	0.0	0.0
	UF+O3	6	>20 % samples below LOQ												NE	NE
	UF+GAC+UV+RO	8	>20 % samples below LOQ												NE	NE

Table S13. Summary of the effectiveness of removal of PhACs by QTTs in both cold and warm seasons of the year, and corresponding data from the literature (in the third column for each QTT, literature is listed below the table).

	UF			GAC			GAC+UV			UF+GAC+UV+NF			UF+O_3			UF+GAC+UV+RO		
	cold	warm	lit.[1-4]	cold	warm	lit.[5-8]	cold	warm	lit.[9]	cold	warm	lit.[10-12]	cold	warm	lit.[7,13-17]	cold	warm	lit.[1,2,4,11,18,19]
**ACET**	-	-	20-50	-	-	ND	-50.0	-29.5	ND	61.6	-	>80	92.2	92.2	>80	92.9	78.3	>80
**ATEN**	-	-	<20	56.0	16.9	>80	45.5	53.5	ND	96.1	96.2	50-80	99.5	99.4	>80	99.4	99.2	>80
**CARB**	-	7.0	<20	36.7	9.2	50-80	29.7	31.6	>80	83.3	83.4	>80	99.9	99.9	>80	99.9	99.9	>80
**CLAR**	-	-	ND	30.7	-	>80	11.2	8.9	>80	99.1	99.5	>80	99.6	99.2	>80	99.6	99.8	>80
**DICL**	-	-	20-50	-	-	>80	54.3	63.7	>80	97.1	97.0	>80	99.7	99.9	>80	99.8	99.9	>80
**ERYT**	-	-	ND	31.1	-	>80	9.8	5.4	ND	98.0	99.4	>80	99.1	98.6	>80	99.6	99.5	>80
**HYDR**	-	-	<20	78.7	24.1	>80	58.4	48.2	ND	61.8	66.2	ND	99.9	99.9	>80	99.8	99.7	>80
**IBUP**	-	-	<20	-	-	>80	67.8	N/A	ND	91.2	N/A	>80	81.4	81.6	50-80	93.8	N/A	>80
**KETO**	-	-	20-50	N/A	-	ND	95.7	52.5	ND	98.6	96.2	ND	91.6	94.5	50-80	98.4	98.2	>80
**NAPR**	-	-	<20	56.1	17.0	ND	32.1	43.3	ND	91.1	92.5	>80	99.5	99.8	>80	99.7	99.7	>80
**SULF**	-	-	20-50	-	-	>80	29.8	17.6	>80	82.9	78.9	>80	99.6	99.6	>80	99.9	99.9	>80
**TRIM**	-	-	20-50	65.6	13.8	>80	46.3	42.5	>80	93.6	93.7	>80	99.6	98.6	>80	99.6	98.6	>80
**ΣPhACs**	-	-	ND	36.4	19.5	-	41.7	47.3	-	86.6	87.6	-	98.9	99.6	-	99.7	99.7	-

<u>Literature to Table S 13</u>

1. Bhattacharjee, S. et al. Fate of emerging contaminants in an advanced SBR wastewater treatment and reuse facility incorporating UF, RO, and UV processes. Water Res 267, 122518 (2024).

2. Ferreiro, C. et al. Contaminants of Emerging Concern Removal in an Effluent of Wastewater Treatment Plant under Biological and Continuous Mode Ultrafiltration Treatment. Sustainability 12, 725 (2020).

3. Chon, K., Cho, J. & Shon, H. K. A pilot-scale hybrid municipal wastewater reclamation system using combined coagulation and disk filtration, ultrafiltration, and reverse osmosis: Removal of nutrients and micropollutants, and characterization of membrane foulants. Bioresour Technol 141, 109–116 (2013).

4. Urtiaga, A. M., Pérez, G., Ibáñez, R. & Ortiz, I. Removal of pharmaceuticals from a WWTP secondary effluent by ultrafiltration/reverse osmosis followed by electrochemical oxidation of the RO concentrate. Desalination 331, 26–34 (2013).

5. Grover, D. P., Zhou, J. L., Frickers, P. E. & Readman, J. W. Improved removal of estrogenic and pharmaceutical compounds in sewage effluent by full scale granular activated carbon: Impact on receiving river water. J Hazard Mater 185, 1005–1011 (2011).

6. Kårelid, V., Larsson, G. & Björlenius, B. Pilot-scale removal of pharmaceuticals in municipal wastewater: Comparison of granular and powdered activated carbon treatment at three wastewater treatment plants. J Environ Manage 193, 491–502 (2017).

7. Yang, X., Flowers, R. C., Weinberg, H. S., & Singer, P. C. (2011). Occurrence and removal of pharmaceuticals and personal care products (PPCPs) in an advanced wastewater reclamation plant. Water Research, 45(16), 5218–5228. https://doi.org/10.1016/j.watres.2011.07.026

8. Knytl, V. et al. Evaluation and comparison of UV/H 2 O 2 and adsorption on active carbon as a tertiary wastewater treatment for pharmaceutical removal within a small WWTP: a pilot study. Environ Sci (Camb) 10, 144–156 (2024).

9. Angeles, L. F. et al. Assessing pharmaceutical removal and reduction in toxicity provided by advanced wastewater treatment systems. Environ Sci (Camb) 6, 62–77 (2020).

10. García-Vaquero, N., Lee, E., Jiménez Castañeda, R., Cho, J., & López-Ramírez, J. A. (2014). Comparison of drinking water pollutant removal using a nanofiltration pilot plant powered by renewable energy and a conventional treatment facility. Desalination, 347, 94–102. https://doi.org/10.1016/j.desal.2014.05.036

11. Kim, S. D., Cho, J., Kim, I. S., Vanderford, B. J. & Snyder, S. A. Occurrence and removal of pharmaceuticals and endocrine disruptors in South Korean surface, drinking, and waste waters. Water Res 41, 1013–1021 (2007).

12. Xu, R. et al. Enhanced micropollutants removal by nanofiltration and their environmental risks in wastewater reclamation: A pilot-scale study. Science of The Total Environment 744, 140954 (2020).

13. Bourgin, M. et al. Evaluation of a full-scale wastewater treatment plant upgraded with ozonation and biological post-treatments: Abatement of micropollutants, formation of transformation products and oxidation byproducts. Water Res 129, 486–498 (2018).

14. Krakkó, D. et al. Application of (V)UV/O3 technology for post-treatment of biologically treated wastewater: A pilot-scale study. Chemosphere 275, 130080 (2021).

15. Lopez, F. J. et al. Removal efficiency for emerging contaminants in a WWTP from Madrid (Spain) after secondary and tertiary treatment and environmental impact on the Manzanares River. Science of The Total Environment 812, 152567 (2022).

16. Nakada, N. et al. Removal of selected pharmaceuticals and personal care products (PPCPs) and endocrine-disrupting chemicals (EDCs) during sand filtration and ozonation at a municipal sewage treatment plant. Water Res 41, 4373–4382 (2007).

17. Reungoat, J. et al. Removal of micropollutants and reduction of biological activity in a full scale reclamation plant using ozonation and activated carbon filtration. Water Res 44, 625–637 (2010).

18. Rodriguez-Mozaz, S. et al. Pharmaceuticals and pesticides in reclaimed water: Efficiency assessment of a microfiltration–reverse osmosis (MF–RO) pilot plant. J Hazard Mater 282, 165–173 (2015).

19. Sahar, E. et al. The use of RO to remove emerging micropollutants following CAS/UF or MBR treatment of municipal wastewater. Desalination 273, 142–147 (2011).
